# Usual Care and Informed Consent in Clinical Trials of Oxygen Management in Extremely Premature Infants

**DOI:** 10.1371/journal.pone.0155005

**Published:** 2016-05-18

**Authors:** Irene Cortés-Puch, Robert A. Wesley, Michael A. Carome, Robert L. Danner, Sidney M. Wolfe, Charles Natanson

**Affiliations:** 1 Critical Care Medicine Department, Clinical Center, National Institutes of Health, 9000 Rockville Pike, Bldg. 10, Room 2C145, Bethesda, Maryland 20892, United States of America; 2 Biostatistics and Clinical Epidemiology Service, Clinical Center, National Institutes of Health, 9000 Rockville Pike, Bldg. 10, Room 2C145, Bethesda, Maryland 20892, United States of America; 3 Health Research Group, Public Citizen, 1600 20th Street NW, Washington, D.C. 20009, United States of America; University of Valencia, SPAIN

## Abstract

**Objective:**

The adequacy of informed consent in the Surfactant, Positive Pressure, and Pulse Oximetry Randomized Trial (SUPPORT) has been questioned. SUPPORT investigators and publishing editors, heads of government study funding agencies, and many ethicists have argued that informed consent was adequate because the two oxygen saturation target ranges studied fell within a range commonly recommended in guidelines. We sought to determine whether each oxygen target as studied in SUPPORT and four similar randomized controlled trials (RCTs) was consistent with usual care.

**Design/Participants/Setting:**

PubMed, EMBASE, Web of Science, and Scopus were searched for English articles back to 1990 providing information on usual care oxygen management in extremely premature infants. Data were extracted on intended and achieved oxygen saturation levels as determined by pulse oximetry. Twenty-two SUPPORT consent forms were examined for statements about oxygen interventions.

**Results:**

While the high oxygen saturation target range (91 to 95%) was consistent with usual care, the low range (85 to 89%) was not used outside of the SUPPORT trial according to surveys and clinical studies of usual care. During usual care, similar lower limits (< 88%) were universally paired with higher upper limits (≥ 92%) and providers skewed achieved oxygen saturations toward the upper-end of these intended ranges. Blinded targeting of a low narrow range resulted in significantly lower achieved oxygen saturations and a doubling of time spent below the lower limit of the intended range compared to usual care practices. The SUPPORT consent forms suggested that the low oxygen saturation arm was a widely practiced subset of usual care.

**Conclusions:**

SUPPORT does not exemplify comparative effectiveness research studying practices or therapies in common use. Descriptions of major differences between the interventions studied and commonly practiced usual care, as well as potential risks associated with these differences, are essential elements of adequate informed consent.

## Introduction

The Surfactant, Positive Pressure, and Pulse Oximetry Randomized Trial (SUPPORT) (Clinical Trials number NCT00233324) [1–3] sought to identify an optimal target range of oxygen saturation (SpO_2_) in extremely premature infants. Infants were randomized to a high (91–95%) or low (85–89%) SpO_2_ target range. The primary outcome was a composite of severe retinopathy of prematurity (ROP) and death before discharge from the hospital.

In a letter to the SUPPORT coordinating center in 2013, the U.S. Office for Human Research Protections (OHRP) found that the informed consent procedure failed to describe “reasonably foreseeable risks and discomforts,” including the risks of blindness and death [4] (see consent forms in S2 Text). Strong criticism of SUPPORT appeared in the lay press and major scientific journals [5–7]. SUPPORT investigators [8], the editor of the journal that published the SUPPORT results [9], many bioethicists [10], and heads of government study funding agencies [11] defended the consent procedure, arguing that SUPPORT represented comparative effectiveness research and that additional risks could not have been foreseen because all interventions were within usual care.

It has been argued that informed consent can be simplified or may not even be necessary for randomized trials in which the interventions being compared: 1) are part of “usual care”; 2) have been used long enough to assume that their associated risks are comparable; and 3) involve patients who would be unlikely to prefer one of the interventions over any other [12]. Accordingly, it has been suggested that SUPPORT should have been eligible for a waiver of informed consent because the investigated oxygen saturation target ranges were within the lower and upper limits of usual care [13].

Although contemporaneous oxygen management in neonatal intensive care units (NICUs) has been described [14, 15], management in SUPPORT has not been rigorously compared to actual usual care. We sought to determine whether oxygen therapy interventions in SUPPORT were consistent with concurrent usual care as documented in the scientific literature. We analyzed and compared usual care to the protocol-specified interventions in SUPPORT and four methodologically similar trials run concurrently—the Benefits of Oxygen Saturation Targeting trials (BOOST II) [16] in Australia, New Zealand (Australian and New Zealand Clinical Trials Registry numbers, ACTRN12605000055606 and ACTRN12605000253606), and the U.K. (Current Controlled Trials number, ISRCTN0084266); and the Canadian Oxygen Trial (COT) (Clinical Trials number NCT00637169) [17]. We found that trial interventions had substantial deviations from published routine clinical practices at the time of the trials.

## Methods

### Systematic Literature Search and Study Selection

To characterize usual care practices concurrent to the five clinical trials, four databases (PubMed, EMBASE, Web of Science, Scopus) were searched (most recently May 15, 2014) for: 1) SpO_2_ target ranges used in NICUs for extremely premature infants since 1990; 2) achieved SpO_2_ levels in the same setting; 3) calibration of and SpO_2_ values from Masimo pulse oximeters (Masimo Radical Pulse Oximeter; Masimo Corporation; Irvine, California), the brand used in SUPPORT and the four similar randomized trials; or 4) data from these five trials. The search was limited to publications in English with additional search terms detailed for each database (see S1 Text). Follow up searches were performed periodically to identify further publications related to the five clinical trials.

Of 470 publications found, 19 provided data on SpO_2_ target ranges or achieved SpO_2_ levels in usual care settings [14, 15, 18–34], four provided relevant information regarding Masimo pulse oximeters [35–38], and eight reported results from the five randomized trials [1, 2, 16, 17, 39–42]. Studies were excluded if they did not contain relevant data, were duplicates, or focused on populations dissimilar from those enrolled in the five trials.

### SUPPORT Consent Forms

To determine how oxygen management interventions were described in SUPPORT consent forms, institutional review board-approved forms were obtained (M.A.C.) for all institutions enrolling infants from the National Institutes of Health (NIH) through the Freedom of Information Act (available in S2 Text).

### Data Extraction

Two investigators (I.C.P. and M.A.C.) independently reviewed each article and the consent forms. Patient characteristics, SpO_2_ target ranges, achieved SpO_2_ values, and pulse oximeter monitoring practices were extracted from each article. Written descriptions of oxygen ranges and potential risks, as provided to parents of potential SUPPORT subjects, were directly excerpted from the consent forms.

### Data Analysis

Because of similarities in gestational ages, monitors used, and sites where care was delivered, detailed analyses were conducted comparing the five trials to corresponding data from the AVIOx study [14]. From 2003 to 2004, the AVIOx study of usual care enrolled 84 infants born at less than 28 weeks gestation and requiring oxygen therapy at 14 NICUs in the U.S., U.K., and New Zealand (including some NICUs that participated in the randomized trials). Notably, infants in the AVIOx study would have met major enrollment criteria for the five clinical trials. During the first four weeks of life, a second pulse oximeter, the Masimo model used in the five randomized trials, was attached to these infants receiving usual care. SpO_2_ readings were recorded continuously each week over 72 hours with the Masimo pulse oximeter, but not displayed to caregivers. Graphs were generated comparing SpO_2_ target ranges and achieved levels for usual care at the 14 AVIOx NICUs to those for the low and high saturation arms studied in the five randomized trials.

### Statistical Methods

The 95% prediction ellipse, for the plot of lower *versus* upper limits of the intended SpO_2_ ranges for each AVIOx NICU, was calculated assuming a bivariate normal distribution between the lower and upper limits within each AVIOx NICU. SAS version 9.3 (SAS Institute Inc., Cary, NC) was used; two-sided p-values of 0.05 or less were considered significant. Achieved median SpO_2_ levels for the high and low groups in the clinical trials were compared to usual care at the AVIOx sites using linear mixed models (LMMs), with a random effect accounting for the variability of results from AVIOx NICUs and the country of study for COT and BOOST II. Similar LMMs were used to compare the percentage of time actual SpO_2_ was below 85% in the low oxygen arms *versus*: (i) the high oxygen arms from the clinical trials; and (ii) the percentage of time actual SpO_2_ was below the lower limit of the intended range during usual care at the AVIOx NICUs with saturation lower limits ≤88%.

## Results

**Fig 1:**
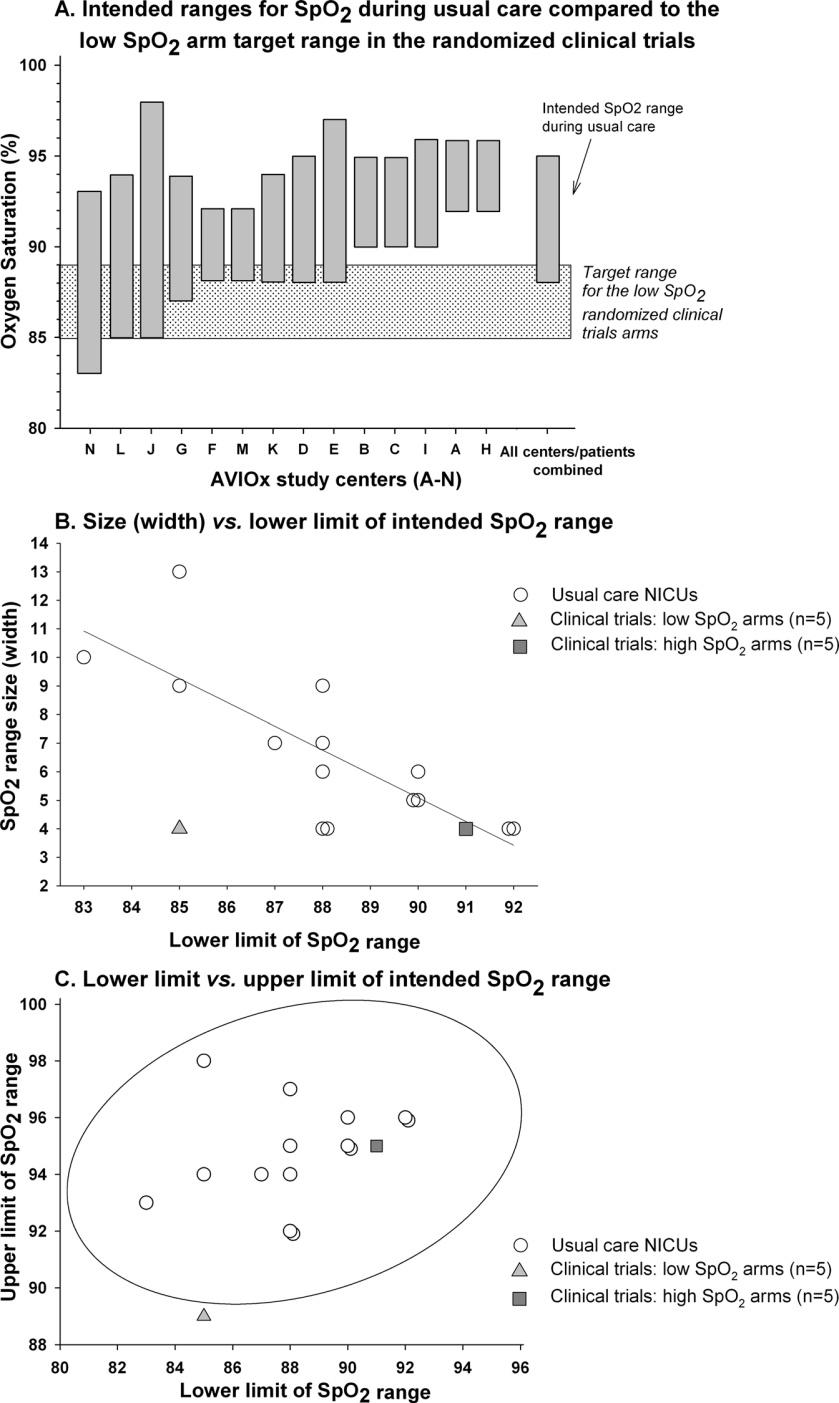
Usual care oxygen saturation (SpO_2_) target ranges in 14 centers for preterm infants (24 to 27 weeks) compared to low and high SpO_2_ target ranges in SUPPORT, COT and BOOST II. In panel A, the usual care SpO_2_ intended target ranges from 14 neonatal intensive care units (NICUs) in the AVIOx study are plotted (dark grey vertical bars). On the X-axis, letters randomly assigned to each of the 14 NICUs in the AVIOx study are provided. NICUs are ordered on the x-axis from the lowest to the highest lower limit of the target range employed. The bar for all centers/patients combined delineates the medians of the upper limits and lower limits of the 14 ranges. The light grey-shaded area represents the low target range studied in the five clinical trials. Panel B shows the relationship of the lower limit of the target ranges (X-axis) to their total width or size (Y-axis) for the individual usual care neonatal intensive care units (NICUs) of the AVIOx study (open circles) and for the low (light grey triangle) and high (dark grey square) SpO2 target range arms of the clinical trials. Panel C shows the relationship of the lower limit (X-axis) to the upper limit (Y-axis) of the same target ranges. The ellipse represents the 95% prediction region for this relationship in the 14 usual care NICUs (see METHODS).

We compared the SpO_2_ target ranges studied in SUPPORT, BOOST II and COT with those intended for use in a comparable population of infants at the 14 centers included in the AVIOx study. The SpO_2_ target range used for the low arm of the clinical trials was lower and narrower than those applied during usual care. Specifically, the upper limit of the low SpO_2_ target range arm (89%) was lower than the upper limit of intended ranges (92 to 98%) used during usual care at all 14 AVIOx NICUs (Fig 1A). Across the 14 AVIOx NICUs, as the lower limit of the intended range decreased, the width of the range increased (Fig 1B). While the high target range in the clinical trials was consistent with this relationship, the low target range was not, being narrower than usual care ranges with comparable lower limits. Unlike the high SpO_2_ target arm, the low arm did not fall within a 95% prediction ellipse for the relationship between the low *versus* high saturation range limits for usual care (Fig 1C).

Published intended SpO_2_ ranges applied during usual clinical care at other NICUs worldwide are remarkably consistent with the AVIOx study data. Two surveys of usual care for preterm infants in the U.S, one presenting intended SpO_2_ ranges for 120 NICUs in 2001 [15] and the other for 40 NICUs in 2004 [18] found that the upper limit of the intended target range was always 92% or greater. Collectively, for more than 100 unique centers worldwide, usual care was reported in surveys, observational studies, and randomized controlled trials to have an SpO_2_ upper limit of 92% or greater with one exception (Table 1). A single study, reporting data collected between 1990 and 1994, had a SpO_2_ target range upper limit as low as 90% [34]. The cohort in this early study experienced a high mortality rate of approximately 50% compared to the 15 to 25% commonly observed in more recent reports.

**Table 1:** Usual care oxygen supplementation practices in preterm infants obtained from surveys, randomized controlled trials and observational studies. Data on usual care SpO_2_ target ranges used and patient or subject characteristics are displayed when available from surveys, randomized controlled trials and observational trials.

Author (year published)	Type of study	Clinical inclusion criteria	Year(s) data collected	Location	Number of centers	Infants' birth weight (g)	Infants' GA (weeks)	Usual care SpO2 target range
***SURVEYS***								
Anderson CG, et al (2004)	Mailed questionnaire to directors of NICUs	Continental USA NICUs; BW < 1500 g	2001	USA	120	< 1500	Not available	**Lower SpO2 limit**: 89.9 + 2.7% (82‐99%) [First 2 weeks], 90.2+2.3% (82‐96%) [After 2 weeks]; **Upper SpO2 limit**: 95.6 + 1.9% (92‐100%) [First 2 weeks], 95.8+ 1.9% (92‐100%) [After 2 weeks]a
Nghiem TH, et al (2008)	Web‐based survey to staff nurses NICUs	USA NICUs with neonatal perinatal fellowships	2004	USA	40	Not available	< 28	**Lower limit SpO2**: 86 + 3% (80‐92%); **Upper limit SpO2**: 94 + 2% (92‐100%)a
***RANDOMIZED CLINICAL TRIALS (Usual Care arms)***								
Claure N, et al (2011)	Randomized crossover trial comparing automatic *vs*. manual FiO_2_ adjustment	Requiring MV and O_2_ Intermittent hypoxemia	2008	USA	4	622 (IQR 568‐770)^#^	25 (IQR 24‐27) at birth ^#^	87‐93%
Hallenberger A, et al (2014)	Randomized crossover trial comparing automatic *vs*. manual FiO_2_ adjustment	GA<37 weeks; Requiring MV/CPAP; FiO2 >0.25	2009‐2012	Germany	4	840 (410‐2460)^##^	26.4 (23‐35.3) at birth; 29.9 (26‐35.6) at enrollment^##^	Target ranges for individual centers: 80‐92%, 83‐ 93%, 85‐94% and 90‐95%
Quine D, et al (2008)	Randomized crossover trial comparing TcPO2 *vs*. SpO_2_ for monitoring oxygen exposure	Preterm infants >24h old with supplemental O_2_	2004‐2005	UK	1	1003 + 416*	27.2 + 2.5*	86‐94%
Schmid MB, et al (2013)	Randomized crossover trial comparing incidence of cerebral desaturations with usual care *vs*. low SpO_2_ target range	GA<34 weeks; Severe intermittent hypoxemia or bradycardia requiring O_2_	2010‐2011	Germany	1	537 (312‐1150)^##^	25.9 (22.6‐30.4)^##^	80‐92%
Urschitz MS, et al (2004)	Randomized crossover trial comparing automatic *vs* manual FiO_2_ adjustment	GA <34 weeks; Requiring NCPAP and O_2_	2002‐2004	Germany	1	800 (600‐2490)^##^	25.5 (24‐33) at birth^##^	87‐96%
***AVIOx and other OBSERVATIONAL STUDIES***								
***AVIOx Study*** Hagadorn JI, et al (2006)	Prospective	GA< 28 weeks and <96h old	2003‐2004	New Zealand, UK, and USA	14	Not available	26.3 (IQR 24.9‐27.4)^#^	**Lower limit SpO2**: 88% (range 83‐92%); **Upper limit SpO2**: 95% (range 92‐98%)
Ahmed SJM, et al (2010)	Prospective	GA<32 weeks Requiring O_2_	Not reported	USA	1	872 (400‐1565)**	26 (24‐31)**	85‐92%
Bhandari V, et al (2009)	Retrospective	BW < 1250g	2002‐2004	USA	2	863 + 198 (managed with SNIPPV) or 964 + 183 (no SNIPPV)*	26.4 + 1.7 (managed with SNIPPV) or 27.9 +2.4 (no SNIPPV)*	Target ranges for individual centers: 85‐95% and 88‐96%
Bizzarro MJ, et al (2014)	Retrospective	BW < 1500g	2004‐2011	USA	1	906g + 278*	26.5 + 2.2*	88‐96% (period I) and 85‐93% (period II)
Clucas L, et al (2007)	Prospective	BW < 1500g or GA<32 weeks admitted within first 24h	2005‐2006	Australia	1	1226g + 354*	29.3 + 2.4*	88‐92% (after 2006, previously it was 90‐95%)
Deulofeut R, et al (2006)	Prospective	BW < 1250g	2000‐2004	USA	2	896 + 211 (period I) / 886 + 219 (period II)*	26.8 + 2.4 (period I) / 27 + 2.4 (period II)*	92‐100% (period I) and 85‐93% (period II)
Laptook AR, et al (2006)	Prospective	BW 501‐1250g Requiring continuous O^2^	2002‐2003	USA	1	847 + 192 (group I) / 873 + 177 (group II)*	27 + 2 (group I)/ 26 + 2 (group II)*	90‐95% (group I) and 88‐94% (group II)
Lim K, et al (2014)	Prospective	GA<37 weeks Receiving CPAP and O^2^	2012	Australia	2	1300 (IQR 930‐1800)#	30 (IQR 27‐32)#	88‐92%
Mills BA, et al (2010)	Prospective	GA<32 weeks or BW<1500g receiving supplemental O_2_	2007	Australia	1	913 + 297*	26.7 + 2*	88‐92%
Sink DW, et al (2011)	Prospective	BW<1500g and GA<29 Weeks	2008	USA	1	860 + 270*	26.6 + 1.6*	85‐92%
Tin D, et al (2001)	Retrospective	GA<28 weeks	1990‐1994	UK	5	Not available	<28	Target ranges for individual centers: 70‐90%, 84‐94%, 85‐95% and 88‐98%
van der Eijk AC, et al (2012)	Prospective	GA< 28 weeks BW<1000g Requiring O_2_ in first 2 weeks life	Not reported	Netherlands	1	760 (545‐935)#	26.3 (24.3‐28)#	88‐94%

BW = birth weight; GA = gestational age; MV = mechanical ventilation; (N)CPAP = (nasal) continuous positive airway pressure; FiO2 = fraction of inspired O2

TcPO2 = transcutaneous oxygen pressure

Data are provided as: * = mean and SD; ** = mean (range); # = median (IQR); ## = median (range)

Intermittent hypoxemia = >4 events of SpO2<80% (Claure et al) or >8 events of SpO2<75% (Schmid et al) within 8h

a Data are means + SD (ranges)

SNIPPV = Synchronized nasal intermittent positive-pressure ventilation.

BW and GA are provided as: * = mean and SD; ** = mean (range); # = median (IQR); ## = median (range)

**Fig 2:**
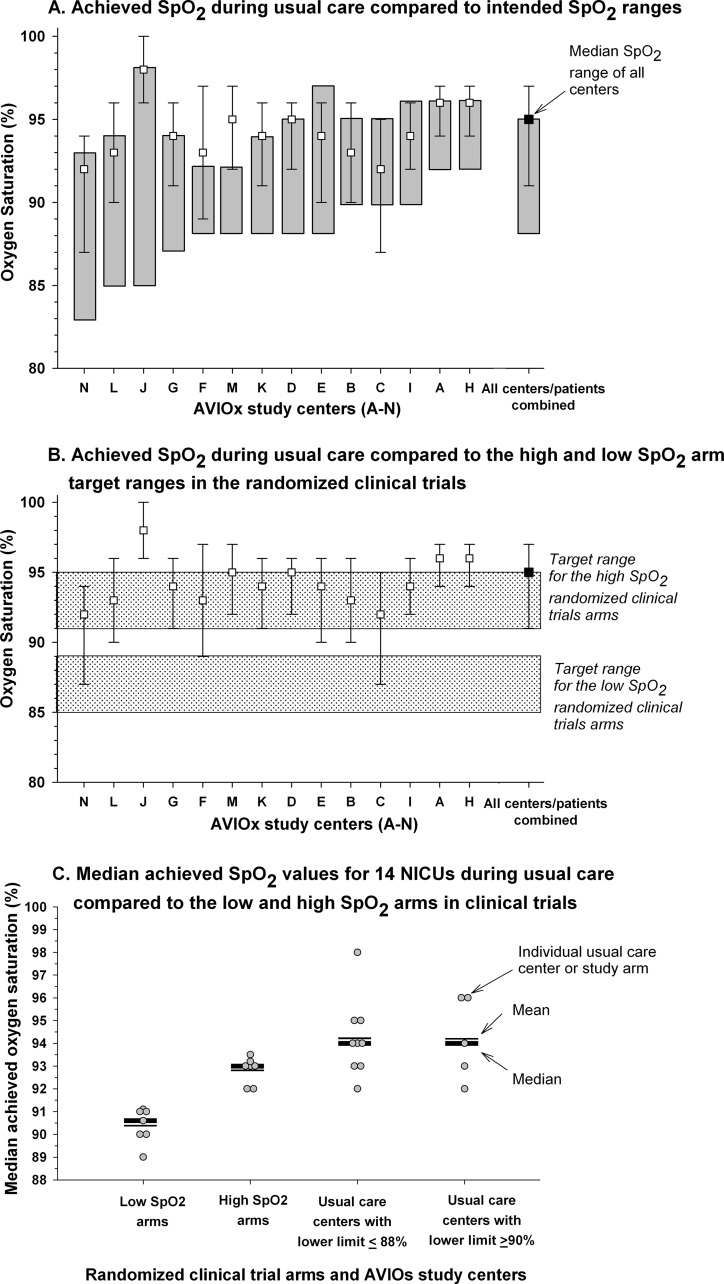
Usual care median achieved SpO_2_ values in 14 care centers for preterm infants receiving oxygen therapy compared to low and high SpO_2_ arms in COT and BOOST II. Panel A compares the median achieved SpO_2_ values, and interquartile range, of each of these 14 usual care NICUs with the intended SpO_2_ range established in the same NICUs (represented by dark grey vertical bars). Panel B compares median achieved SpO_2_ values, and interquartile range, from these 14 NICUs to the target ranges of the low (lower grey-shaded area) and high (upper grey-shaded area) SpO_2_ arms of the SUPPORT, BOOST II and COT trials. In panel C median achieved SpO_2_ values are plotted in 7 low and 7 high SpO_2_ arms during the BOOST II and COT trials, as well as in the 14 NICUs included in the AVIOx study; the latter are separated into 9 centers using a lower limit of the intended SpO_2_ target range at or below 88% and 5 centers using a lower limit of the intended target SpO_2_ range ≥90%. This separation was done to compare usual care to the clinical trial arms with comparable lower limits of the intended target SpO_2_ ranges. For each of four compared groups, the median (thick horizontal line) and the mean (thin horizontal line) of the achieved SpO_2_ values are plotted. The number of study arms is 7 for each target range because in three trials (BOOST II Australia and U.K. and COT) the data were provided separately from before and after recalibration of the Masimo pulse oximeters.

Next, we compared achieved SpO_2_ values during usual care at the AVIOx centers with those achieved by the low and high arms of the BOOST II and COT trials (median achieved SpO_2_ values were not available for SUPPORT). During COT and BOOST II in Australia and the U.K., Masimo pulse oximeter calibration software was revised to correct a 1% to 2% overestimation of oxygen saturation measurements, especially between values of 87% to 90% (see S1 Fig). Data before and after recalibration have been analyzed separately for these three trials. Notably, median achieved SpO_2_ values during usual care in AVIOx NICUs were skewed toward or above the upper limit of intended ranges at all centers but one, center C (Fig 2A). Thus, achieved saturation values in clinical practice extensively overlapped with those targeted by the high, but not the low SpO_2_ arms of the clinical trials (Fig 2B). Achieved SpO_2_ values during usual care at all AVIOx NICUs were well above the low target range of the five randomized trials. In all but three AVIOx centers, the 25th percentile of achieved SpO_2_ values was above the upper limit of the low target range (Fig 2B). Accordingly, median achieved SpO_2_ levels in the low target arms of BOOST II and COT were significantly lower than those achieved at the nine AVIOx NICUs that targeted ranges with similar lower limits (≤88%) (p = 0.003, Fig 2C). In contrast, median achieved SpO_2_ levels in the high target arms were not significantly different from the AVIOx NICUs, whether compared to the AVIOx centers with relatively low (≤88%; 9 NICUs) or high (≥90%; 5 NICUs) lower limits.

**Fig 3:**
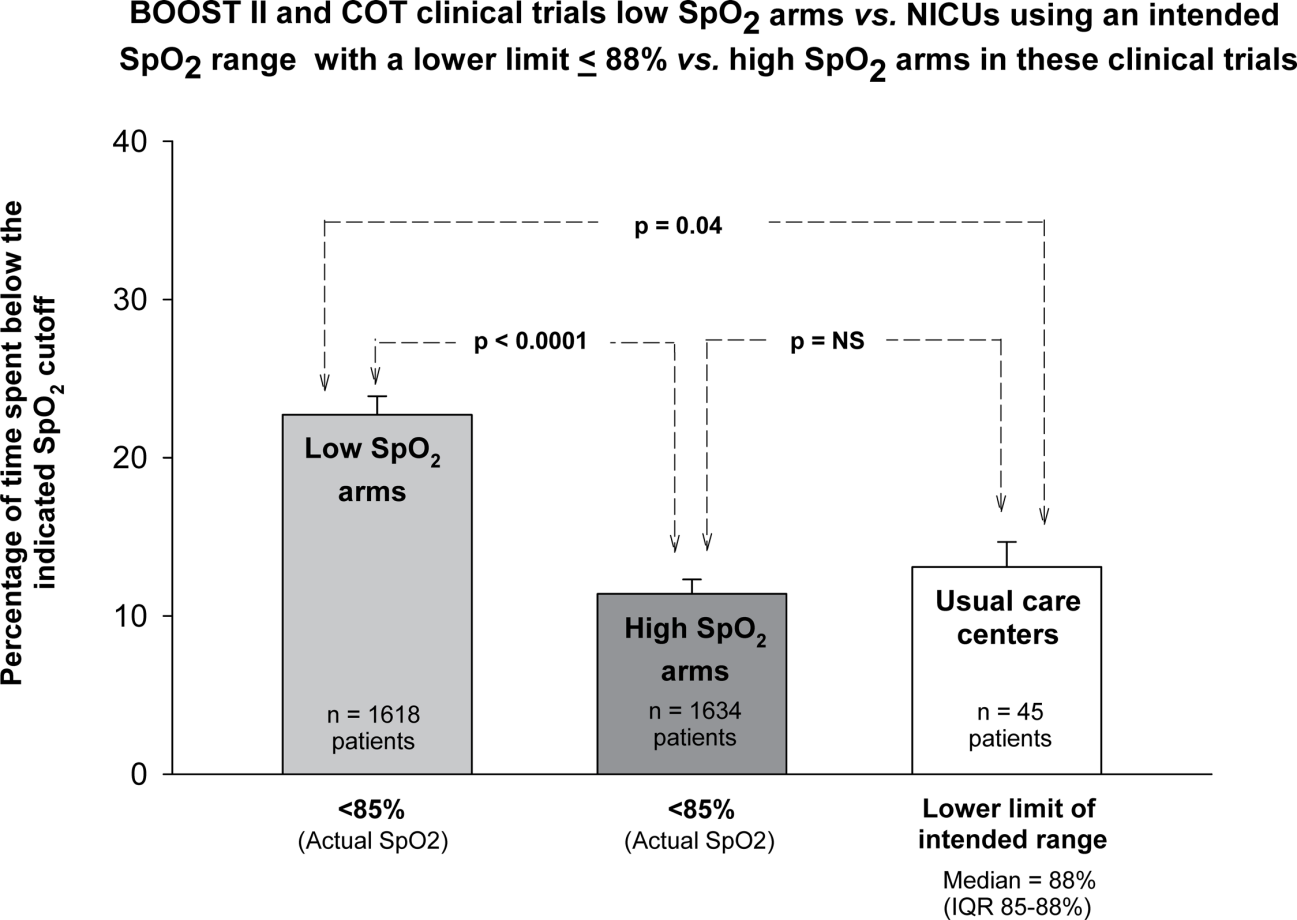
Percentage of time spent below an oxygen saturation (SpO_2_) value of 85%, and below the intended SpO_2_ range. The mean ± SE of the percentage of time spent below a true SpO_2_ value of 85% for 7 low SpO_2_ arms from the BOOST II and COT trials (light grey bar) is plotted *versus* the time spent below 85% for 7 high SpO_2_ arms from the same trials (dark grey bar) *versus* the time spent below the lower limit of the intended range for the nine usual care neonatal intensive care units from the AVIOx study that had with comparable lower limits of the intended range (median lower limit of 88%; white bar). The number of low and high SpO_2_ arms is 7 because in three trials (BOOST II Australia and U.K. and COT), the data were provided separately for before and after recalibration of the Masimo pulse oximeters.

Infants randomized to the low SpO_2_ arms of COT and BOOST II (for which published data is available) spent almost twice as much time below the lower limit of their intended target range (85%) as those receiving usual care at the nine AVIOx NICUs with lower limits ≤88% (p = 0.04). Subjects randomized to the low SpO_2_ arms of COT and BOOST II also spent significantly more time below a true saturation value of 85% than infants randomized to the high arms (p<0.0001) (Fig 3), as expected from their target ranges.

Finally, none of the consent forms acknowledged that the low SpO_2_ arm was an experimental intervention, not a widely practiced subset of usual care, and therefore posed risks, some of which were foreseeable, some less well-understood. Twenty of twenty-two SUPPORT consent forms explicitly or implicitly described the oxygen ranges studied as standard of care, usual care, or as a desired approach in some units (Table 2). Eleven consent forms had statements indicating that there was no predictable increase in risk to infants enrolled in the study, and two had statements indicating that there was no more risk to subjects than those seen in premature infants needing NICU management. Two forms (institutions I and V) did not have such characterizations of the oxygen ranges and risks. All consent forms for the BOOST II and COT trials were not available to us and were not analyzed.

**Table 2:** Excerpts from SUPPORT informed consent forms. * A selection of statements extracted from the 22 institutional review board-approved SUPPORT consent forms that characterized the oxygen management interventions are displayed in a tabular format. Institutions are blinded in this table.

A	“Each of the 4 possible combinations of treatments is considered **standard care** by some units in the United States.” “All of the treatments (CPAP in the delivery room, delivery room intubation plus surfactant, lower oxygen range, and higher oxygen range) proposed in this study are **standard of care** at various hospitals like [institution F] in the United States, so there are no predictable increases in risk for your baby.”
B	“Each of these 4 possible treatment groups is considered the **standard of care** in the NICU at [institution G].” “The treatments talked about in this study are all **standard of care**. …There is no predictable increase in risks above **standard of care** for your baby.” “Both of the **ranges for oxygen used** in this study are within the range that we currently use in our NICU.”
C	“We will also be looking at the ranges of oxygen saturation that are **currently being used** with these same babies.” “All of these saturations are considered **normal ranges** for premature infants.” “Sometimes higher ranges are used and sometimes lower ranges are used. All of them are acceptable ranges.”
D	“All of these saturations are considered **normal ranges** for premature infants.” “Sometimes higher ranges are used and sometimes lower ranges are used. All of them are **acceptable ranges used** in different institutions.” “…each of the 4 possible combinations of treatments is considered by some units to represent their **desired approach**.” “Because all of the treatments proposed in this study are **standards of care** at different hospitals across the country, there is no predictable increase in risk for your baby.”
E	“Keeping the level in either end of the **normal range** is **routinely used** in the NICU for premature babies.” “This will determine if your baby will have his/her oxygen saturation level kept in the high or low part of the **normal oxygen saturation range**.” “Your infant will have al [sic] **usual care** for infants born before 28 weeks gestation.” “The oxygen saturation ranges to be used are currently used for **usual care** in premature infants in the NICU.”
F	“… your infant will either be on the high end or the low end of the **normal oxygen saturation** that we normally use in our intensive care nursery.” “All the treatments in this study are **currently used** in the intensive care nursery and most infants born at the same age as your infant will receive all those treatments during their stay in the intensive care nursery.” “The **standard of care** at [institution E] neonatal intensive care nurseries varies with the attending doctor taking care of your infant and may be similar to any of the above 4 groups of therapies that the research is studying.”
G	“Within the range of oxygen which we normally use, your infant will either be on the high end of **normal** or the low end of **normal**.” “…each of the 4 possible combinations of treatments is **currently used** by some NICUs as their **primary approach** to treating premature infants.” “Because all of the treatments proposed in this study are **standard of care**, there is no expected increase in risk for your infant”
H	“Sometimes higher ranges are used and sometimes lower ranges are used. All of them are **acceptable ranges**” “All of these saturations are considered **normal ranges** for premature babies.”
I	“… your baby will have his/her oxygen saturation level kept in the high or low part of the **normal oxygen saturation range**.”
J	“Both [oxygen] groups are within the range of **usual standard of care** for the NICU.” “Once your baby is admitted to the NICU, he/she will receive the **standard care** according to the policies and procedures set by the NICU.”
K	“Both of these ranges are within the oxygen saturation range that is **currently used** for premature infants in the NICU at [institution K].” “All of these **treatments** have been carefully studied and all are used in Newborn ICUs.” “All of these treatments are **currently clinically accepted**, but haven’t been compared with each other in this manner …” “For this study, there will be no change in the oxygen saturation range from the one that is **currently used** in the NICU at [institution K].”
L	“This will allow us to keep the saturations at the high and low ends of the **normal range**…” “Because all of the treatments proposed in this study are within **standard of care**, there is no predictable increase in risk for your baby.” “…each of the 4 possible combinations of treatments is considered by some NICUs to represent their **desired approach**.”
M	“Within the range of oxygen that we normally keep babies in (85 to 95%), your baby will either be in the high end of **normal** or the low end of **normal**.” “Your baby will receive all **standard care** provided to any baby in the Neonatal Intensive Care.” “The procedures that are being used are **standard (routine) treatments** used in neonatal intensive care. … To the best of our understanding, there will be no more risks for the baby in this study than are possible for any ill premature baby needing intensive care.”
N	“Each of the 4 possible combinations of treatments is considered by some hospitals to represent their **desired standard approach**.” “This will allow us to keep the saturations at the high or low end of the **normal range**…” “This study does not pose significant risks beyond those inherent in a sick premature baby. … All treatments are **standard of care** at some NICUs across the country”
O	“**Routine neonatal intensive care** will be provided during your baby's participation in the study.” “Each of the study treatments is **already being used** by many doctors across the country, there is no predictable increase in risk for your baby.”
P	“Within the range of oxygen which we normally keep babies in, your baby will either be on the high end of **normal** or the low end of **norma**l.” “Because all of the treatments proposed in this study are **standard of care**, there is no predictable increase in risk for your baby.” “… each of the 4 possible combinations of treatments is considered by some units to represent their **desired approach**.”
Q	“There are also two oxygen support strategies: 1) a low **normal** range (85‐ 89%) and 2) a high **normal** range (91‐95%).” “Because all treatments proposed in this study are **currently accepted standard of care**, there is no predictable increase in risk to your baby.” “… because all of the treatments proposed in this study are **currently accepted as standard of care**, there is no unpredictable increase [in risk] expected.”
R	“All treatments proposed in this study are **currently accepted standard of care**. All of these treatment options may have risks but there is no known predictable increase in risk to your baby from any one approach.” “The particular treatment or procedure may involve risks to the baby that are currently unforeseeable but because all of the treatments proposed in this study are **standard of care**, there is no unpredictable increase.”
S	“The oxygen saturation level currently used in the neonatal intensive care units at [institution S] is between 85% and 94%, so both treatment groups (the group for whom the target for oxygen saturation levels will be 85‐89% and the group for whom the target for oxygen saturation levels will be 91‐95%) will be treated with oxygen in a manner that is very similar to that **currently used** at both hospitals” “The ranges used in this study are in **common use** in NICU’s across the country.” “Because all of the treatments proposed in this study are **standard of care**, there is no predictable increase in risk for your baby.”
T	“Within the range of oxygen which the doctors normally keep babies in, my baby will either be on the high end of **normal** or the low end of **normal**.” “Because all of the treatments proposed in this study are **standard of care**, there is no predictable increase in risk for my baby.”
U	“We will also be looking at the ranges of oxygen saturation that are **currently being used** with these same babies.” “Another part of the study will be looking at the ranges of oxygen saturations that are **currently being used** with premature infants.” “All of these saturations are considered within the **normal** range for premature infants.” “Each of the 4 possible treatment combinations is considered to represent the **best approach** by some units.” “Since this study will compare **standard** therapies, you or your insurer will be responsible for the cost of medical care provided by the staff of the [institution V] to your infant.” “As described above, participation in this study will compare **standard** treatment strategies.” “Whether or not your infant participates, he/she will be cared for according to **standards** of newborn care.”
V	

* CPAP and Surfactant were part of a 2X2 design in this study. These therapies are not discussed because they are beyond the scope of this paper

## Discussion

In five randomized trials of supplemental oxygen for extremely preterm infants, the high SpO_2_ arms, with target saturations of 91 to 95%, reflected a range well within the scope of usual care. In contrast, for the low arms, targeting saturations of 85 to 89%, the upper limit was lower and the target range much narrower than concurrent usual clinical practice. The full range of clinical practice does encompass the bottom-end (85%) of the SpO_2_ targets investigated in these studies. However, relatively low, bottom-end saturation limits in usual care were universally paired with upper limits of 92% or greater, creating wider ranges. Importantly, caregivers appear to have a strong tendency to skew actually achieved saturations toward or above the upper end of these ranges. Consistently, low alarm limits in usual care are adhered to more stringently than upper alarm limits [27, 31]. However, in the trials, the narrow low SpO_2_ arm target range together with protocolized care blinded by offset pulse oximeters [21, 35, 38] resulted in infants spending significantly more time below an SpO_2_ of 85% compared to either usual care or the high saturation arm. As such, these infants experienced significantly more severe desaturation events [41].

All five trials used pulse oximeters programmed to display offset SpO_2_ values, to mask caregivers to trial group assignments. A careful analysis by COT investigators indicated that the transition zones from the 3% offset to the true saturation values impacted bedside care [35]. Each arm used one rapid and one slow transition zone to taper the 3% offset back to true values at each end of the target ranges. In the rapid transition zones, displayed SpO_2_ values changed up to 4% over the course of a 1% change in true values. In the slow transition zones, the displayed oxygen saturation remained fixed (e.g., at 84%), while true values decreased 3% (e.g., 87% to 84%) [3, 35, 43]. According to the COT investigators, “the masking algorithm and its transition from offset to true values may have had an important and unexpected impact on the titration of oxygen therapy” [35]. The COT investigators suspected that caregivers avoided the instability of displayed SpO_2_ values in the rapid transition zones by favoring saturation values at the bottom of the high target range and at the top of the low target range, in order to reduce the frequency of alarms [35].

Prior to starting BOOST II, an audit was initiated at participating centers to evaluate the performance of Masimo pulse oximeters [36]. Selected centers evaluated 176 preterm infants receiving usual care with the Masimo device. This study found that the Masimo pulse oximeters had a calibration error that overestimated SpO_2_, especially between values of 87 to 90% (see S1 Fig) [36]. As a consequence of this study, Masimo corrected their calibration algorithm, improving the accuracy of this monitoring device. Thus, before this correction, infants were placed on less accurate pulse oximeters as part of enrollment in SUPPORT and other similar trials. Other commercially available pulse oximeters, more commonly used in the United States [15] did not have this problem [36, 37].

As the COT investigators demonstrated [35] blinding caregivers using the masking algorithm “may have adversely affected the implementation of the protocol” [35]. Both study arms were differentially managed in an unanticipated manner relative to one another and to usual care, confounding the interpretation of study outcomes [35]. Blinding can be necessary for the validity of research, but needs to be carefully designed and preliminarily assessed in pilot studies to avoid unanticipated problems. This is particularly important in critically ill patients with high mortality rates, where blinding caregivers to a vitally important clinical parameter has the potential to increase risks. Additional pilot studies evaluating the offset pulse oximeters may have avoided changes to the calibration algorithm after the start of enrollment and provided information important to safety monitoring.

A literature review of oxygen exposure in extremely premature infants yielded only one prospective, high-quality, observational study. Despite this notable limitation to our analysis, the AVIOx study [14] collected robust data for comparing the low and high SpO_2_ treatment arms in these five trials to usual care. Further, the intended ranges in the AVIOx study centers were consistent with reported practices from two U.S. surveys, one presenting intended SpO_2_ ranges for 120 NICUs [15] and the other for 40 NICUs [18]. Similar European surveys were not identified with fully comparable premature infants. However, in a survey of 228 NICUs in the UK, 92% of responding centers maintained premature infants with respiratory distress syndrome or bronchopulmonary dysplasia at SpO_2_ levels between 90 to 98% [44]. Overall, more than 100 unique centers worldwide reported usual care practices compatible with AVIOx (Table 1). SUPPORT, therefore, was not representative of comparative effectiveness research, as commonly understood.

Unfortunately, problems in study design and informed consent processes often only come to public attention with the occurrence of harm. A recent meta-analysis of these five clinical trials found a significant increase in mortality in the low *versus* the high oxygen saturation arms, but only after recalibration of the Masimo pulse oximeters [45]. ROP also showed significant heterogeneity across trials, but, unlike mortality, this variability was not associated with changes to the calibration of the pulse oximeters during the course of some of the trials [45]. A patient-level meta-analysis (NeoPROM) is planned that will hopefully clarify some sources of this unresolved heterogeneity. Of note, the incidence of NEC, a condition associated with a high mortality rate, was consistently higher in the low oxygen saturation arms than in the high arms with no significant heterogeneity [45]. This was the only major toxicity consistently found across all trials and calibration schemes. The potential for real harm to subjects in complex clinical trials that alter delivered clinical care underscores the need for a consent process that fully discloses whether research subjects will receive an intervention as commonly practiced at the institutions enrolling subjects or an experimental intervention that significantly deviates from usual care practices and that may pose both foreseeable and less well-understood risks. This is particularly true for therapies routinely titrated based on perceptions of clinical need in critically ill patients and other vulnerable, high-risk populations [46].

In rapidly lethal conditions with high mortality rates, basic interventions such as oxygen therapy may be lifesaving, and protocol-driven changes in their administration can have serious consequences. A thorough review of available literature, combined with detailed surveys of usual care and appropriately designed pilot studies, can provide important information regarding how trial interventions might affect care relative to usual clinical practices. These achievable steps might have preemptively uncovered the differential impact of the masking algorithm on oxygen saturation targeting [35] and clarified for investigators and institutional review boards that one of the interventions differed markedly from usual care.

SUPPORT consent forms have been at the core of the controversy surrounding this trial. It is necessary for subjects to make informed decisions that consent forms disclose how the interventions studied differ from usual care. Our analysis of the scientific literature indicates that the narrow, low saturation target range studied in these oxygen trials was not commonly used. In addition, the COT investigators elegantly demonstrated that the offset pulse oximeters also altered oxygen management in unexpected ways. Describing how oxygen management in at least one of the study arms differed from usual care, as well as the potential risks posed by such modifications, were both critical to providing adequate informed consent.

Despite being within the 85 to 95% target range recommended by the American Academy of Pediatrics [47], the low SpO_2_ target range studied in SUPPORT and the other four trials had an upper limit of 89% that was below those upper limits used during usual care. Similarly, many other sub-ranges, such as 85 to 86% or 94 to 95%, would not have been usual or standard of care and cannot be assumed to be safe. At the time of these five trials, our literature review found that most NICUs targeted SpO_2_ ranges with a lower limit between 85 and 89%, but always combined with an upper limit between 92 and 95%. In addition, achieved SPO_2_ values measured at the bedside often skewed higher than these target ranges. Notably, our literature review of usual care was limited to publications written in English and therefore most reports were from North America, UK, Australia and New Zealand. As such, we cannot rule out the possibility that different SpO_2_ target ranges were being used in non-English speaking regions or countries.

In conclusion, our findings highlight the need for investigators, prior to designing clinical trials, to rigorously evaluate actual clinical practices at institutions intending to enroll subjects. Likewise, institutional review boards need access to such data before approving protocols and consent forms. This is particularly important for research purported to be testing interventions consistent with usual care.

## Supporting Information

S1 Fig Supplemental Figure. (PDF)

S1 Text Supplemental Methods and Figure Legends. (DOCX)

S2 Text IRB-Approved SUPPORT Consent Forms. IRB-Approved SUPPORT Consent Forms presented in the order cited in Table 2. Excerpted text presented in Table 2 is highlighted in each consent form.
(PDF)
