# Supplementary material for: Usual Care and Informed Consent in Clinical Trials of Oxygen Management in Extremely Premature Infants
Source: PLoS One. 2016 May 18;11(5):e0155005. doi: 10.1371/journal.pone.0155005 (PMC4871545; doi:10.1371/journal.pone.0155005)
Supplement: S1 Fig — (PDF) [file pone.0155005.s001.pdf]

S1 Fig

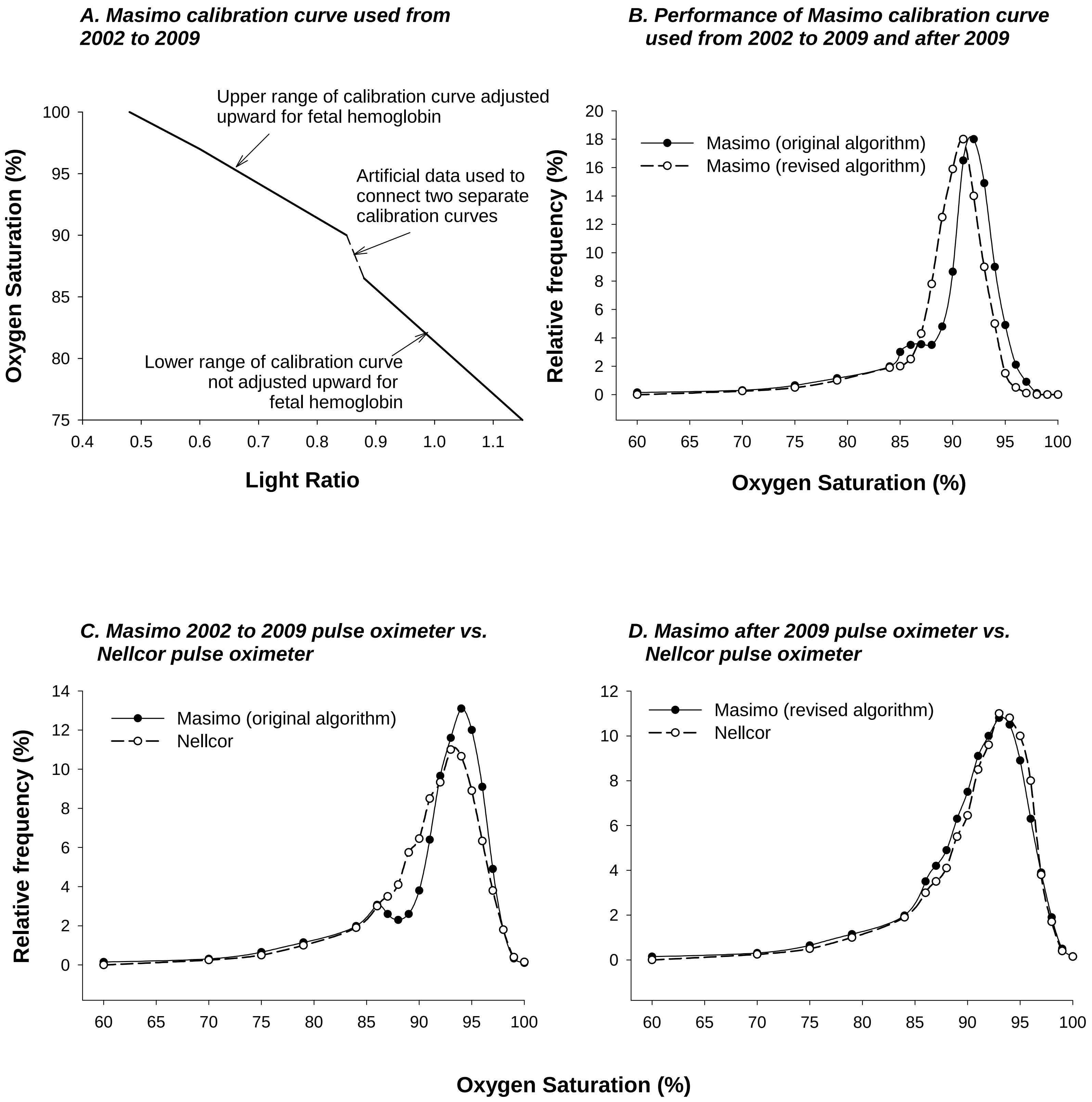

A. Masimo calibration curve used from 2002 to 2009
Upper range of calibration curve adjusted upward for fetal hemoglobin
Artificial data used to connect two separate calibration curves
Lower range of calibration curve not adjusted upward for fetal hemoglobin
Oxygen Saturation (%)
100
95
90
85
80
75
0.4
0.5
0.6
0.7
0.8
0.9
1.0
1.1
Light Ratio
B. Performance of Masimo calibration curve used from 2002 to 2009 and after 2009
Masimo (original algorithm)
Masimo (revised algorithm)
Relative frequency (%)
20
18
16
14
12
10
8
6
4
2
0
60
65
70
75
80
85
90
95
100
Oxygen Saturation (%)
C. Masimo 2002 to 2009 pulse oximeter vs. Nellcor pulse oximeter
Masimo (original algorithm)
Nellcor
Relative frequency (%)
14
12
10
8
6
4
2
0
60
65
70
75
80
85
90
95
100
D. Masimo after 2009 pulse oximeter vs. Nellcor pulse oximeter
Masimo (revised algorithm)
Nellcor
12
10
8
6
4
2
0
60
65
70
75
80
85
90
95
100
Oxygen Saturation (%)
