# Supplementary material for: Usual Care and Informed Consent in Clinical Trials of Oxygen Management in Extremely Premature Infants
Source: PLoS One. 2016 May 18;11(5):e0155005. doi: 10.1371/journal.pone.0155005 (PMC4871545; doi:10.1371/journal.pone.0155005)
Supplement: S1 Text — (DOCX) [file pone.0155005.s002.docx]

**S1 Text. Supplemental Methods and Figure Legends**

**Supplemental Methods**

*Databases and search strategies*

PubMed

(spo2[ti] OR “oxygen saturation”[ti] OR “oxygen saturations”[ti] OR “pulse oximeter”[ti] OR “pulse oximeters”[ti] OR “pulse oximetry”[ti]) AND (“premature infant”[ti] OR “premature infants”[ti] OR “preterm infant”[ti] OR “preterm infants”[ti] OR prematurity[ti] OR gestation[ti] OR gestational[ti]) Filters: English

(oxygen/blood[majr] OR oximetry[majr] OR spo2[tiab] OR “oxygen saturation”[tiab] OR “oxygen saturations”[tiab] OR “pulse oximeter”[tiab] OR “pulse oximeters”[tiab] OR “pulse oximetry”[tiab]) AND (infant, premature[mesh] OR “premature infant”[tiab] OR “premature infants”[tiab] OR “preterm infant”[tiab] OR “preterm infants”[tiab] OR prematurity[tiab] OR “24 week gestation”[tiab] OR “24 weeks gestation”[tiab] OR “25 week gestation”[tiab] OR “25 weeks gestation”[tiab] OR “26 week gestation”[tiab] OR “26 weeks gestation”[tiab] OR “27 week gestation”[tiab] OR “27 weeks gestation”[tiab] OR “28 week gestation”[tiab] OR “28 weeks gestation”[tiab] OR “29 week gestation”[tiab] OR “29 weeks gestation”[tiab] OR “30 week gestation”[tiab] OR "30 weeks gestation”[tiab]) AND (target[tiab] OR targets[tiab] OR targeting[tiab] OR “saturation range”[tiab] OR “saturation ranges”[tiab] OR “spo2 range”[tiab] OR “spo2 ranges”[tiab] OR “saturation level”[tiab] OR “saturation levels”[tiab] OR “spo2 level”[tiab] OR “spo2 levels”[tiab] OR “median saturation”[tiab] OR “median spo2”[tiab] OR “mean saturation”[tiab] OR “mean spo2”[tiab] OR “saturation value”[tiab] OR “saturation values”[tiab] OR “spo2 value”[tiab] OR “spo2 values”[tiab] OR intended[tiab] OR achieved[tiab] OR actual[tiab] OR documented[tiab] OR guideline[tiab] OR guidelines[tiab] OR “standard practice”[tiab] OR “standard practices”[tiab] OR “standard of practice”[tiab] OR “standards of practice”[tiab] OR policy[tiab] OR policies[tiab] OR physician’s practice patterns[mesh] OR organizational policy[mesh] OR guideline adherence[mesh] OR practice guidelines as topic[mesh] OR evidence based practice[mesh] OR reference values[mesh]) Filters: English

EMBASE

spo2:ti OR 'oxygen saturation':ti OR 'oxygen saturations':ti OR 'pulse oximeter':ti OR 'pulse oximeters':ti OR 'pulse oximetry':ti AND ('premature infant':ti OR 'premature infants':ti OR 'preterm infant':ti OR 'preterm infants':ti OR prematurity:ti OR gestation:ti OR gestational:ti) AND [english]/lim

'oxygen blood level'/exp/mj OR 'blood oxygen tension'/exp/mj OR 'oximetry'/exp/mj OR spo2:ab,ti OR 'oxygen saturation'/exp/mj OR 'oxygen saturation':ab,ti OR 'oxygen saturations':ab,ti OR 'pulse oximeter'/exp/mj OR 'pulse oximeter':ab,ti OR 'pulse oximeters':ab,ti OR 'pulse oximetry'/exp/mj OR 'pulse oximetry':ab,ti AND ('prematurity'/exp OR 'premature infant':ab,ti OR 'premature infants':ab,ti OR 'preterm infant':ab,ti OR 'preterm infants':ab,ti OR prematurity:ab,ti OR '24 week gestation':ab,ti OR '24 weeks gestation':ab,ti OR '25 week gestation':ab,ti OR '25 weeks gestation':ab,ti OR '26 week gestation':ab,ti OR '26 weeks gestation':ab,ti OR '27 week gestation':ab,ti OR '27 weeks gestation':ab,ti OR '28 week gestation':ab,ti OR '28 weeks gestation':ab,ti OR '29 week gestation':ab,ti OR '29 weeks gestation':ab,ti OR '30 week gestation':ab,ti OR '30 weeks gestation':ab,ti) AND (target:ab,ti OR targets:ab,ti OR targeting:ab,ti OR 'saturation range':ab,ti OR 'saturation ranges':ab,ti OR 'spo2 range':ab,ti OR 'spo2 ranges':ab,ti OR 'saturation level':ab,ti OR 'saturation levels':ab,ti OR 'spo2 level':ab,ti OR 'spo2 levels':ab,ti OR 'median saturation':ab,ti OR 'median spo2':ab,ti OR 'mean saturation':ab,ti OR 'mean spo2':ab,ti OR 'saturation value':ab,ti OR 'saturation values':ab,ti OR 'spo2 value':ab,ti OR 'spo2 values':ab,ti OR intended:ab,ti OR achieved:ab,ti OR actual:ab,ti OR documented:ab,ti OR guideline:ab,ti OR guidelines:ab,ti OR 'standard practice':ab,ti OR 'standard practices':ab,ti OR 'standard of practice':ab,ti OR 'standards of practice':ab,ti OR policy:ab,ti OR policies:ab,ti OR 'clinical practice'/exp/mj OR 'health care policy'/exp/mj OR 'practice guideline'/exp/mj OR 'evidence based practice'/exp/mj OR 'reference value'/exp/mj) AND [english]/lim

Web of Science

TI=(spo2 OR "oxygen saturation" OR "oxygen saturations" OR "pulse oximeter" OR "pulse oximeters" OR "pulse oximetry") AND TI=("premature infant" OR "premature infants" OR "preterm infant" OR "preterm infants" OR prematurity OR gestation OR gestational) Refined by:LANGUAGES: ( ENGLISH )

Scopus

(TITLE(spo2 OR "oxygen saturation" OR "oxygen saturations" OR "pulse oximeter" OR "pulse oximeters" OR "pulse oximetry")) AND (TITLE("premature infant" OR "premature infants" OR "preterm infant" OR "preterm infants" OR prematurity OR gestation OR gestational)) AND (LIMIT-TO(LANGUAGE, "English"))

Cochrane Central Register for Controlled Trials

'(spo2 OR "oxygen saturation" OR "oxygen saturations" OR "pulse oximeter" OR "pulse oximeters" OR "pulse oximetry") AND ("premature infant" OR "premature infants" OR "preterm infant" OR "preterm infants" OR prematurity OR gestation OR gestational) in Record Title in Trials

ClinicalTrials.gov

"premature infant" OR "premature infants" OR "preterm infant" OR "preterm infants" OR prematurity OR gestation OR gestational | spo2 OR "oxygen saturation" OR "oxygen saturations" OR "pulse oximeter" OR "pulse oximeters" OR "pulse oximetry"

**SUPPLEMENTAL FIGURE LEGENDS**

***S1 Fig. Calibration of Masimo pulse oximeters before and after modification***

These figures have been plotted using information provided in Johnston ED et al.[^42^](#_ENREF_42) In panel A, we show a simulation of how the original calibration curve (used from 2002 to 2009) in Masimo pulse oximeters was constructed. Pulse oximeter sensors measure the ratio of red to infrared waveform intensities, and the device converts these ratios into displayed SpO_2_ values using a calibration curve. Here, the SpO_2_ displayed value (Y-axis) is plotted *versus* the ratio of red to infrared waveform intensities (X-axis). Two different curves were used in the Masimo monitors, one for SpO_2_ values below 87% and one for SpO_2_ values above 90%. The upper curve (for SpO_2_ values above 90%) had an upward adjustment obviating the effects of low levels of circulating fetal hemoglobin, while the lower curve (for SpO_2_ values below 87%) did not have the upward shift. These two calibration curves were connected using an artificial line that is steep and short, therefore artificially displaying higher SpO_2_ levels for each change in light ratio. Other brands of pulse oximeters used a single calibration curve for the entire range of SpO_2_ values.

Panel B shows the frequency histogram for the percentage of time spent at each displayed SpO_2_ values between 60% and 100% using the Masimo original calibration curve (used from 2002 to 2009) in comparison to the revised calibration curve (used after 2009). In 2009, Masimo revised the calibration scheme and modified it to rely on a single curve instead of two curves connected by a steep slope. As shown in this panel, when the original and revised algorithms are compared, the original calibration curve produced a histogram with a reduced frequency of displayed values between 87% and 90% in comparison to the revised curve.

Panels C and D show the frequency histograms in a similar fashion as panel B, but now comparing data from a Masimo pulse oximeter with the original (panel C) or revised calibration scheme (panel D) to a pulse oximeter from a different brand (i.e., Nellcor). In panel C, the original calibration scheme of the Masimo device displayed a lower frequency of SpO_2_ values 87-90% in comparison to the Nellcor pulse oximeter. In contrast, as shown in panel D, once the calibration scheme was revised and a single calibration curve was used, both pulse oximeters had a similar frequency histogram of displayed SpO_2_ values.
