# Supplementary material for: Usual Care and Informed Consent in Clinical Trials of Oxygen Management in Extremely Premature Infants
Source: PLoS One. 2016 May 18;11(5):e0155005. doi: 10.1371/journal.pone.0155005 (PMC4871545; doi:10.1371/journal.pone.0155005)
Supplement: S2 Text — IRB-Approved SUPPORT Consent Forms presented in the order cited in Table 2. Excerpted text presented in Table 2 is highlighted in each consent form. (PDF) [file pone.0155005.s003.pdf]

S2 Text. IRB-Approved SUPPORT Consent Forms

IRB-Approved SUPPORT consent forms, presented in the order cited in Table 2.

Excerpted text presented in Table 2 is highlighted in each consent form.

Form
M0345

DUKE UNIVERSITY HEALTH SYSTEM

Consent To Participate In A Research Study

The SUrfactant Positive Airway Pressure and Pulse Oximetry Trial in Extremely Low Birth Weight Infants: The SUPPORT Trial

Introduction:

You are being asked to allow your child to be in this study because there is a possibility he/she will be born between 12 and 16 weeks early (24-28 weeks gestational age). Dr. Michael Cotten and the Neonatology clinical research group at Duke Medical Center and colleagues at the 16 centers in the National Institutes for Child Health and Human Development (NICHD) Neonatal Research Network are conducting a research study to determine the best way to help extremely premature babies breathe better after they are born. This study will compare the effectiveness, risks and benefits of two different types of treatment for premature lung disease. The two methods being compared are CPAP (positive pressure applied with a face mask or nasal prongs to help keep the lungs inflated) in the delivery room and reduced use of a breathing machine to assist the baby's breathing *versus* intubation (using a breathing tube placed in the trachea or windpipe) in the delivery room and more aggressive use of mechanical ventilation (breathing for the baby using a machine). This study is also being done to learn the appropriate levels of oxygen saturation (oxygen levels in the blood) that should be maintained in extremely premature babies while they are being treated in the hospital. Dr Cotten and the Neonatology clinical research group are receiving salary support from the NICHD to conduct this study.

Purpose of the Study:

1) To compare the outcome of infants who receive delivery room CPAP and who have restrictive guidelines for having a breathing tube placed for mechanical ventilation with infants who have the tube placed and surfactant (a liquid which helps babies with immature lungs breath easier by helping keep their lungs from collapsing) given in the delivery room and have less strict guidelines for maintaining a breathing tube and mechanical ventilation.

2) To compare low range (85-89%) oxygen saturation levels with high range (91-95%) levels to determine if a lower range results in decreased ROP (Retinopathy of Prematurity, an eye disease that may result in impairment of vision or even blindness) which may be caused by excessive levels of oxygen.)

Duration of the Study: We expect to include about 1300 babies in the study from the 16 NICHD Neonatal Research Network hospitals around the country over a two year period. 60 babies will take part at Duke.

Background:

CPAP vs. Intubation/Surfactant. Research studies of surfactant treatment along with intubation and mechanical ventilation reduced mortality versus intubation and mechanical ventilation with no surfactant treatment. Other studies have suggested that many extremely premature babies may not need intubation or treatment with surfactant if they are treated with CPAP in the delivery room. Research studies suggest that avoiding intubation may help babies avoid lung damage associated with premature birth and subsequent mechanical ventilation, even if surfactant is used. Delivery room intubation and surfactant treatment has never been compared to CPAP treatment started in the delivery room.

DUKE UNIVERSITY HEALTH SYSTEM

Consent To Participate In A Research Study
The SUrfactant Positive Airway Pressure and Pulse Oximetry Trial in Extremely Low Birth Weight Infants: The SUPPORT Trial

Some hospitals in the United States that provide care for extremely preterm infants like yours use CPAP in the delivery room, while other similar hospitals intubate every baby in the first hour of life and give all the infants between 24 and 28 weeks gestation surfactant. The decision as to which to use is currently made by the physician attending the delivery. This study will help doctors find out if it is better to intubate in the delivery room and treat infants like yours with surfactant in the first hour of life, or to start them on CPAP in the delivery room and try to limit the chance that they will need intubation.

Oxygen Level Study. The current alarm limits for oxygen saturation monitors in many neonatal intensive care units (NICU's) in the United States are set at 85% and 95%. The aim in many units is to keep oxygen saturations between 88 and 92%. This goal is based on research suggesting high levels of oxygen (> 95% saturation) in the first weeks of life are related to later development of ROP and damage to the lungs. We are not sure how much lower we should set the goal for oxygen saturation in extremely preterm infants and still avoid increasing the risk of developmental delay. Previous studies have suggested that risk of ROP decreases when oxygen saturation goals were set with a lower limit of 80% without any increase in risk in neurodevelopmental impairment in the first year after discharge. Currently in the United States, some hospital NICU's target oxygen levels in the lower end of the 85 – 95% range, while others target the higher range. Both treatment groups in this study (85-89% and 91-95%) fall within the alarm range currently used at Duke. The study will attempt to keep babies in one of these two ranges.

Each of the 4 possible combinations of treatments is considered standard care by some units in the United States.

Study Procedures:
Prior to delivery, and after your permission is given, your baby will be randomized (chosen by chance like the flip of a coin) to one of two lung treatment strategies. The treatments are as follows:

1) *CPAP* in the delivery room immediately after birth and continuing in the NICU, *or*

2) *Intubation* (placement of a tube in his/her trachea or windpipe) in the delivery room followed by surfactant administration and mechanical ventilation.

In addition to being randomly assigned to one of the two groups described above, your baby will be randomized to a High reading or Low reading oxygen saturation monitor, also known as an "oximeter" (a monitor that displays how much oxygen is in the blood). The oximeters used in this study are FDA approved, and they have been modified for research purposes. This modification makes the monitors show a value which is either slightly higher or slightly lower than the true oxygen level when values are between 85 and 95%. Outside those ranges, the oximeter works the same as the standard of care oximeter.

Only the study coordinator will know which oxygen group your baby is in. Neither you nor the doctors, nurses, and respiratory therapists taking care of your baby will know which oximeter group your baby is in. Within the alarm range of the usual oxygen saturation monitors which we normally use, your baby will either be on the high end of normal or the low end. He/she will remain on this device until he/she reaches 36 weeks adjusted age (for example, 24 wks gestation plus 12 weeks of age = 36 weeks adjusted age). In the case of an emergency, the study doctor can quickly find out which oxygen saturation group your baby is in.

We will also collect information on how well your baby gains weight and grows. Other care will be conducted as normal during his/her participation in the study. Your baby will be followed periodically in our Special Infant Care Clinic (SICC) during the first 2 to 3 years of life. At these visits we check your baby's health and development. For this study, we will ask you to allow us to report the results of your baby's neurologic evaluation and developmental testing at the 18-22 months corrected age follow-up clinic visit.

Your baby will have routine ultrasounds of his/her head during his/her stay in the NICU. A copy of the head ultrasounds conducted between 4-14 days of life and at the time of the original expected due date will be collected for this study. If your baby's head ultrasound at the time of the expected due date occurs before 35 weeks post-menstrual age, your baby will receive another head ultrasounds for the purpose of this study between 35-42 weeks post-menstrual age. In addition, your baby will have a MRI (Magnetic Resonance Imaging) for the purpose of this research study between 35-42 weeks post-menstrual age. Neither the head ultrasound nor the MRI is experimental and both are currently used on infants at Duke University Medical Center. The MRI is a specialized brain scan that takes detailed pictures of the brain structure and can detect normal and abnormal brain tissue. If conducted for the purpose of this study, the head ultrasound and /or MRI will be paid for by the grant. If your baby is still in the NICU, your baby will need to be transported to the MRI suite in order to have the procedure. Only those patients considered stable for transport will undergo an MRI. If your baby is discharged home prior to 35-42 weeks PMA, you maybe asked to return to Duke University Medical Center for the head ultrasound and MRI procedures. The MRI will be conducted after your baby is swaddled while sleeping following a feeding and with the use of a jacket-like device that allows the baby to lie still without using sedation. A physician at the hospital in which you receive the head ultrasound and MRI will provide you with an interpretation of the results of the tests. Neither the ultrasound nor the MRI involves exposure to radiation. Please initial your choice below.

(_______) Yes, I agree to allow my baby to receive an MRI.

(_______) No, I do not agree to allow my baby to receive an MRI.

DUKE UNIVERSITY HEALTH SYSTEM

Consent To Participate In A Research Study
The SUrfactant Positive Airway Pressure and Pulse Oximetry Trial in Extremely Low Birth Weight Infants: The SUPPORT Trial

We know that many babies born as early as your baby are at risk for breathing problems, especially wheezing and coughing during early childhood, after discharge from the NICU. The purpose of Breathing Outcomes Study described here is to determine the effect of the SUPPORT Study treatment on your baby's respiratory health in early childhood, during the first 18-22 months after his/her expected due date if he or she was to have been born at full term.

You and your infant's participation will begin with an interview at the time of your baby's first regular follow-up visit with the Special Infant Care Clinic (SICC). At this interview Dr. Goldstein or one of her research associates will ask you questions about your family, including questions about family history of breathing problems, and questions about your home, including things that may increase your child's risk of breathing problems. You do not need to answer any questions that make you uncomfortable. The interview will take about 15 minutes.

We will continue to stay in touch with you and your infant by telephone or in person at one of your subsequent SICC visits The main study center at the University of Rochester has trained interviewers who will call you by telephone every 6 months over the next 18-22 months, a total of three times. At these times, they will ask questions about your child's breathing (especially wheezing and coughing), medication use, and visits to a doctor, emergency room, or hospital visits for treatment of breathing problems. They will also ask you several questions about your family and yourself. The entire call should take about 15 minutes of your time, less if your baby has had no breathing problems.

They will schedule the telephone calls at a time that is convenient for you. The telephone calls will occur when your infant is 6, 12, and 18 months after his/her expected delivery at full term.

The results from your baby's questionnaire will be combined with other infants from around the country. However, your baby's name will never be included in any information that leaves Duke. Please initial your choice below.

(_______) Yes, I agree to be contacted.

(_______) No, I do not agree to be contacted

Risks :

Participation in this study may involve some added risks or discomforts. All of the treatments (CPAP in the delivery room, delivery room intubation plus surfactant, lower oxygen range, and higher oxygen range) proposed in this study are standard of care at various hospitals like Duke in the United States, so there are no predictable increases in risk for your baby.

Infants randomized to the CPAP group may, at some point in their care, require intubation and assisted ventilation (use of a breathing machine). The study provides guidelines for this, but if the attending physician deems this necessary at any time, participation in the study will not affect this decision

regardless of the study guideline. With CPAP, there is a small risk of skin damage to the nose. Your baby's nurses and doctors will closely monitor your baby's nose while he/she is on CPAP. Risks of surfactant therapy include slowing of the heart and a decrease in oxygen saturation during dosing as the surfactant liquid passes through the breathing tube into the lungs. Surfactant treatment may cause a rapid increase in how much the lungs expand with each breath. Trained respiratory therapists, nurses and physicians will monitor your baby for these effects during and after surfactant dosing.

The risks of higher versus lower oxygen saturation levels in the two ranges to be used in this study are unknown. Lower oxygen range may help infants avoid ROP and chronic lung disease (need for supplemental oxygen at 36 weeks post-conception or discharge) which has been associated with neurodevelopmental delay and higher oxygen levels may or may not have an effect on the risk of neurodevelopmental delay. It is unclear which oxygen level, if any, will help babies grow better. Both ranges are within the alarm limits set in infants like yours in the Duke NICU. Some unknown risks may be learned during the study. If these occur, you will be informed by the study personnel.

There are no known effects from exposure to magnetic fields (MRI). Temporary minor skin irritation from tape used to apply MRI-compatible monitoring electrodes may occur, but this risk is unlikely.

The only other risk of this study is the risk to confidentiality. Every effort will be made to keep your child's medical record confidential. There will be no name or other patient identification in any study report that may be published after the study is completed. Measures taken to protect you and your baby's identity are described in the confidentiality section of this document.

Benefits:
There may be benefits to your child directly from participation in this study. The knowledge gained from this study may help us improve treatment of babies in the future.

Data Handling and Confidentiality:
Clinical information will be collected from your baby's chart by Duke Neonatology Clinical Research study personnel. Information will be labeled with a code number. Coded information will be sent to the NICHD Neonatal Network's Data Collection Center at Research Triangle Institute (RTI) in Research Triangle Park, North Carolina. The study log linking the code number with your baby's identity will be kept under lock and key at Duke. Information directly identifying your baby will not leave Duke. Research records will be kept confidential to the extent provided by law, and will be maintained in the study record until your child reaches the age of 21 years or six years after the study is complete, whichever is longest. Your baby's study record may be reviewed in order to meet federal or state regulations. Reviewers of your child's study record may include representatives from the Food and Drug Administration, representatives of the National Institutes of Health (NIH) and the Duke Institutional Review Board (IRB, the hospital committee that supervises research in human beings). When research results from this study are reported in a professional setting, such as in a medical journal or at a scientific meeting, the identity of research subjects taking part in the study is withheld. If disclosed by the sponsor, the information is no longer covered by the federal privacy regulations.

Costs and Compensation:

There is no charge to you or your child for participating in this research. You or your child's insurance will be responsible for paying the cost of routine medical care for your child's condition. The costs for your child's routine care will appear on his/her hospital bill. Although there is no compensation for allowing your child to participate in this project, we will give you $25 and a parking pass to help with travel expenses to the clinic for the 18 – 22 month visit.

Injuries:

Immediate necessary medical care is available at Duke University Medical Center in the event that your child is injured as a result of participation in this research study. However, there is no commitment by Duke University, Duke University Health System, Inc., or your child's Duke physicians to provide monetary compensation or free medical care to your child in the event of a study-related injury. For questions about the study or a research –related injury, contact Dr. Mike Cotten at 919-681-0630 during regular business hours and at 919-970-4381 after hours and on weekends and holidays.

Alternatives to Participation:

As an alternative to participation in this study, you may decide to have your baby's doctor decide which treatment your baby will receive. If you decide not to include your child in this study, none of his/her medical information will be included in the study data. Participation in research is entirely voluntary.

Right not to Participate or to Withdraw:

You may choose for your child not to be in the study, or, if you agree for your child to be in the study, you may withdraw him/her from the study at any time. If you withdraw him/her from the study, no new data will be collected for study purposes unless the data concern an adverse event (bad effect) related to the study. If such an adverse event occurs, we may need to review your child's entire medical record. All data that have already been collected for study purposes, and any new information about an adverse event related to the study, will be sent to the sponsor.

Your decision for your child not to participate or to withdraw from the study will not involve any penalty or loss of benefits to which you or your child are entitled, and will not affect your access to health care at Duke. If you do decide for your child to withdraw, we ask that you contact Dr. Cotten in writing and let him know that you are withdrawing from the study. His mailing address is: DUMC Box 3179, Durham, North Carolina, 27710. If you withdraw your child from the study, the attending physician will decide whether to maintain current treatment or change it, based on your child's needs at the time of the decision.

In addition, your child's doctor may decide to take him/her off this study if her/his condition gets worse, he/she has serious side effects, or the study doctor determines that it is no longer in your child's best interest to continue. The sponsor or regulatory agencies may stop this study at anytime without your consent. If this occurs, you will be notified and your child's study doctor will discuss other options with you. We will tell you about new information that may affect your child's health, welfare, or willingness to stay in this study.

Form
M0345

DUKE UNIVERSITY HEALTH SYSTEM

Consent To Participate In A Research Study
The SUrfactant Positive Airway Pressure and Pulse Oximetry Trial in Extremely Low Birth Weight Infants: The SUPPORT Trial

Whom to Call with Questions or Problems:

For questions about the study or a research-related injury, contact Dr. Cotten at 919-681-6025 during regular business hours and at 919-970-4381 (pager) or the Duke paging operator, 919-684-8111 after hours and on weekends and holidays. During regular business hours, you may also call the clinical research pager at 919-970-1425 and speak to a member of the Neonatology Clinical Research team. For questions about your child's rights as a research participant, contact the Duke University Health System Institutional Review Board (IRB) Office at (919) 668-5111.

Statement of Consent:

"The purpose of this study, procedures to be followed, risks and benefits have been explained to me. I have been allowed to ask questions, and my questions have been answered to my satisfaction. I have been told whom to contact if I have additional questions. I have read this consent form and agree for my child to be in this study, with the understanding that I may withdraw him/her at any time. I have been told that I will be given a signed copy of this consent form."

______________________________________ ____________

Signature of Parent/Guardian **Date**

______________________________________ ____________

Signature of Person Obtaining Consent **Date**

Emory University School of Medicine
Consent to be a Research Subject

Title: The Surfactant Positive Airway Pressure and Pulse Oximetry Trial in Extremely Low Birth Weight Infants

Principal Investigator: Susie Buchter, M.D., P.I.
Barbara J. Stoll, M.D., Co-P.I.

Sponsor's Name: National Institute of Child Health and Human Development (NICHD)

Introduction/Purpose:

You are being asked to volunteer your baby for a research study. There is a possibility that your baby will be born between 16 and 12 weeks early (24-28 weeks gestational age). Babies born this early usually have difficulty breathing. Their lungs are not mature enough to work well so that the babies can breathe on their own. Most all babies born at this early age will need assistance breathing and or extra oxygen. If needed, this support begins at birth in the delivery room.

This study will look at the use of CPAP in the delivery room. CPAP is positive pressure applied with a facemask to help keep the lungs inflated. This study will also look at the levels of oxygen saturation (oxygen levels in the blood) in premature babies.

It is known that the breathing problems of babies can be improved by putting a liquid in the lungs (surfactant). This liquid is put into the lungs by placing a tube in the windpipe (intubation). Afterwards, breathing is supported with a breathing machine and or extra oxygen.

However, when babies get this support for a long time, their lungs can become injured. This may cause the baby to be dependent upon the extra support for a long time. Studies from Europe have suggested that early CPAP can reduce the need for intubation in very premature infants.

There is no standard way to use CPAP/Positive End Expiratory Pressure for resuscitation in the delivery room for tiny premature infants. This pressure is given using a mask placed on the baby's face. The pressure may also be given using prongs placed in the infant's nostrils. The pressure is produced using current breathing machines. There are also special devices that are designed to deliver such pressures.

Studies have suggested that the use of early CPAP and trying not to use a breathing machine may have a better outcome for babies. These babies may also have a decreased need for surfactant therapy. These babies may also have a decreased need for additional oxygen. The current commonly used treatment is surfactant administration. Surfactant is produced by the normal lung. It is lacking in very preterm infants. Its use has been connected with a decrease in death and respiratory problems. Both the uses of CPAP and surfactant may be good. There has not been a study to compare the use of CPAP with surfactant treatment begins after delivery and continues in the NICU.

Retinopathy of Prematurity (ROP) is a common eye problem in tiny premature infants. Blood vessels that nourish the preterm infant's eyes are not fully developed. Small vessels in the retina (part of the eye) may have periods of increased or rapid and wild growth. Over time ROP can get

better or get worse. Usually ROP will heal without any problems. If the ROP is worse than usual, there is a chance that the blood vessels will grow out of control. If this happens, surgery may be needed to prevent scars inside the eye. These scars can cause severe vision loss.

Getting extra oxygen for a long time can also damage the baby's eyes. Another study has shown that less eye surgery was needed in special nurseries using lower oxygen limits.

There are two purposes of this study. First of all, we will compare two different types of care in the delivery room. We will compare infants who receive delivery room CPAP and those who have a breathing tube and surfactant given. Secondly, we will compare low range (85-89%) oxygen saturation levels with a high range (91-95%). We want to know if a lower oxygen level in babies can prevent this bad eye problem.

This study is being performed at Grady Memorial Hospital and Crawford W. Long Hospital. Fifteen other medical centers in the U.S. are also part of this study. The National Institute of Child Health and Human Development (NICHD) sponsors this research. Your baby will only be eligible for this study if you deliver between 24 and 27 weeks. The study will last about two years and will enroll about 1300 infants nationally. About 100 babies will be studied at Emory.

Procedures:

If you agree to this study and give consent for your baby, the following will happen. Prior to delivery, your baby will be assigned to one of two treatments. Assignment will be random (like flipping a coin). In the first treatment group, your baby would be placed on CPAP in the delivery room to help with their breathing. If your baby is in the second treatment group, a tube will be placed in his/her trachea (windpipe) to help with their breathing. After the tube is placed, a dose of Surfactant will be given in the tube. Both of these treatment groups are current standards of care for preterm babies in the delivery room.

At the same time that your baby is assigned to the above treatment group, he/she will also be randomly (like flipping a coin) assigned to a high or low oxygen monitoring group. Your baby will be treated using either a lower (85-89%) or higher (91-95%) oxygen saturation range. We will start this monitoring of oxygen saturation by 2 hours of age. Both of the ranges for oxygen used in this study are within the range that we currently use in our NICU. In this study we will try to maintain your baby within one of these 2 ranges. Each of these 4 possible treatment groups is considered the standard of care in the NICU at Emory. The study will take place during the entire time your baby is on oxygen while he/she is in the NICU.

A final eye exam will be done at 3 months corrected age (3 months after your due date for this baby). If your baby has been discharged from the hospital prior to this, an outpatient eye appointment will be scheduled. This eye appointment is part of standard of care. As part of the study, we will collect information about the eye exam. We may need to request a copy of your baby's eye exam if it is done after discharge.

As part of this study we will follow your baby's growth. A research nurse will measure your baby's length and the measurement around their head. We will collect your baby's weight from their chart. We will collect information about feedings your baby receives from the baby's chart. This information will be collected up to eight different times beginning on the day your baby is born and ending the day your baby is discharged to go home with you. We want to know if babies in this study grow at different rates or the same rate.

We want to know if your baby has breathing problems after they go home. We also want to know if your baby needs extra care to help them with their breathing. As part of this study we will follow your baby and collect information from you at discharge and after the baby goes home. We will call you by phone to get information when your baby is about 6, 12, and 18 months old. Each phone call will take about 15 minutes of your time. A member of the research team will ask you questions about the health of different people your baby comes in contact with. You will be asked questions about your baby's breathing. You will be asked questions about any visits your baby might have to the doctor, clinic, emergency room and hospital admissions. We will give you a brochure to help you record the information we will need about your baby.

Your baby will be followed for this study until he/she is discharged from the hospital. As part of standard of care the Emory Developmental Progress Clinic (DCP) will follow your baby. At 18 months of age, he/she will be seen in the DPC as part of our routine follow-up program. At the 18 month visit you will be asked to allow your baby to be part of a follow up study. You will be asked to sign a separate consent. Information will be shared with the NICHD Follow-up Study of very tiny premature babies. At that time we will look at your baby's growth. We will also perform physical and neurological testing and developmental testing.

Risks:
The treatments talked about in this study are all standard of care. However, intubation, administration of surfactant and CPAP are not without risk. Risks of intubation may include improper placement of tube and windpipe damage from the tube. Risks of surfactant could be unequal distribution of the liquid and bleeding into the lungs. Another risk could be delayed surfactant use while CPAP is being used. Risks of CPAP could be damage to the nose and overinflation of the lungs. There is no predictable increase in risks above standard of care for your baby. Some unknown risks may be learned during the study. If this happens, the research team will let you know.

Benefits:
If either treatment group is found to be a better way to treat babies, your baby may benefit from the study. But taking part in this study may not benefit your baby. Doctors may learn new things that may help babies in the future.

Alternatives:
You may choose not to have your baby take part in this study and your baby will continue to get the standard of care. The current standard of care at Grady and Crawford Long is to assess the baby's breathing at birth. The majority of babies born at this early date will be intubated and given surfactant. A few will receive CPAP in the delivery room or later in the intensive care nursery. Both of the ranges for oxygen used in this study are within the range that we currently use in our NICU.

Confidentiality:
People other than those doing the study may look at both medical charts and study records. Agencies that make rules and policy about how research is done have the right to review these records. So do agencies that pay for the study. Those with the right to look at your baby's study records include the Food and Drug Administration, the Office for Human Research Protections, National Institute of Child Health and Human Development, the Emory University Institutional Review Board, and Grady's Research Oversight Committee. Records can also be opened by court order. We will keep your baby's records private to the extent allowed by law. We will do this

even if outside review occurs. We will use a study number rather than your baby's name on study records where we can. Your baby's name and other facts that might point to him/her will not appear when we present this study or publish its results.

If you are or have been a patient at an Emory Healthcare facility, then you will have an Emory Healthcare medical record. If you are not and have never been a patient at an Emory Healthcare facility then no Emory Healthcare medical record will be created for you just because you are participating in a research study.

Results from study tests and procedures that are performed, analyzed and/or read at or for Emory Healthcare facilities that can be used for healthcare purposes will be placed in any medical record that you have with Emory Healthcare facilities. In addition, a copy of the informed consent form and HIPAA authorization form that you sign will be placed in any Emory Healthcare medical record you may have. Persons who have access to your medical record will be able to have access to all results and documents that are placed there, and the results/documents may be used by Emory Healthcare facilities to help provide you with medical care. Any results and documents that are kept as part of your medical record are not covered by certain state and federal laws and regulations that may prevent the disclosure of, research data. However, the confidentiality of the results and other documents in the medical record will be governed by laws such as HIPAA that concern medical records.

Emory University does not have any control over results from tests and procedures performed and/or analyzed or read at non-Emory Healthcare facilities. These results are NOT routinely included in medical records at Emory Healthcare facilities, and they will not necessarily be available to Emory Healthcare providers. Emory University also does not have control over any other medical records that you may have with other healthcare providers and will not send any test or procedure results from the study to these providers. It is up to you to let these healthcare providers know that you are participating in a clinical trial.

Some tests and procedures that may be performed during this study by Emory Healthcare or other facilities or persons MAY NOT BE LOOKED AT OR READ FOR ANY HEALTHCARE TREATMENT OR DIAGNOSTIC PURPOSES. THESE TESTS AND PROCEDURES WILL ONLY BE LOOKED AT FOR RESEARCH PURPOSES AND THE RESULTS WILL NOT BE REVIEWED TO MAKE DECISIONS ABOUT YOUR PERSONAL HEALTH OR TREATMENT. The specific types of tests or procedures, if any, that fall within this category are listed below: NONE

Costs and Compensation:

There will be no cost to you or your baby for being in this study. You will not be paid for being in this study. If your baby is injured as a result of this research, medical care will be available. However, Emory University (including Crawford Long Hospital) and the Grady Health System have not set aside funds to pay for this care or to compensate you if a mishap occurs. If you believe your baby has been injured by this research, you should contact Dr. Susie Buchter, the investigator in charge at 404-778-1450.

Contact Persons:

Call Dr. Susie Buchter at (404) 778-1450 if you have questions about this study or if you feel your baby has been harmed from being in this study. If you have any questions or concerns about your rights as a participant in this research study, contact Colleen Dilorio, Ph.D., Chairman, Emory University Institutional Review Board, at (404) 712-0720. If your baby is a patient at Grady

Hospital you may contact Dr. Curtis Lewis, Senior Vice President for Medical Affairs, at (404) 616-4261.

New Findings:

We may learn new things during the study that you may need to know. We can also learn about things that might make you want to stop participating in the study. If so, you will be notified about any new information.

Voluntary Participation and Withdrawal:

Participation in the study is voluntary. You have the right to refuse to let your baby be in this study. If you decide to let your baby be in this study and change your mind, you have the right to drop out at any time. This decision will not affect in any way your baby's current or future medical care. This decision will not affect any other benefits to which you are otherwise given.

Although your infant will be treated according to a specific plan (protocol), individual circumstances may arise. In such cases, your infant's health will always be considered more important than strictly following the study. Changes will be discussed before they are made whenever possible.

We will give you a copy of this consent form to keep.

If you're willing to volunteer your baby for this research, please sign below.

____
Subject's name

______________________________ ___________ ___________
Subject's legally authorized representative ***Date*** ***Time***

______________________________ ___________ ___________
Person Obtaining Consent ***Date*** ***Time***

IRB#: 1158-2004
Consent Form Approval Period
FROM: 9/25/07 TO: 9/24/08
AUTHORIZATION: rm

Informed Consent

Title of Research: The Surfactant Positive Airway Pressure and Pulse Oximetry Trial in Extremely Low Birth Weight Infants (SUPPORT Study) (Multicenter Network of Neonatal ICU's)

Title of Secondary Research: Neuroimaging and Neurodevelopmental Outcome (MRI Study)

Postnatal Growth of Infants Enrolled in SUPPORT Study (Growth Study)

UAB IRB Protocol Numbers: F040910010, X060418004 and F050922007

Investigators: Dr. Wally Carlo and Dr. Namasivayan Ambalavanan

Sponsor: National Institute of Child Health and Development (NICHD)

You are being asked to give your permission for your baby to participate in a study designed to determine if using positive airway pressure during resuscitation after birth helps decrease the severity of lung disease in premature babies. We will also be looking at the ranges of oxygen saturation that are currently being used with these same babies. You and your baby were selected as possible participants because you are less than 28 weeks pregnant and your baby may be born prematurely. The doctors at UAB, along with 15 other centers across the country, are participating in this project sponsored by the by the National Institute of Child Health and Human Development.

This consent form gives you information about the research study, which a member of the research team will discuss with you. This discussion should go over all aspects of this research: its purpose, the procedures that will be performed, any risk of the procedures, and possible benefits. Once you are informed about this study, you will be asked if you want your baby to participate; if so, you will be asked to sign this form.

Introduction

If born prematurely, your baby is at risk for a breathing problem called **Respiratory Distress Syndrome (RDS).** A baby's lungs are made up of tiny air sacs; each one is supposed to open and close as the baby breathes in and out. Oxygen is supposed to go in and carbon dioxide is supposed to come out. This works well in full term babies and adults; however, in premature babies, the lung sacs don't always work this way. Some lung sacs open and close normally; others collapse and stick together when the baby breathes out making it harder for the baby to breathe. Doctors treat this problem with expanding breaths and pressure to keep the lungs slightly inflated between those breaths. Keeping a little air pressure in the lungs after the baby

UAB-IRB
Consent Form Approved 06-04-08
Expiration Date 04-30-09

____
Parents' Initials or Those of Legally Authorized Representative

breathes out (resting pressure) makes it easier for the baby to take the next breath. Sometimes a medication called *surfactant* is given to try to help keep the lung sacs expanded. After your baby is born, if he/she needs help breathing, the doctor or nurse will place a resuscitation bag over the baby's nose and mouth to provide oxygen and manual breaths. The bag is squeezed to force air into the baby's lungs. The bag and mask may be used to give breaths or give just pressure to keep the lungs inflated between breaths. This resting pressure is called continuous positive airway pressure or CPAP or PEEP.

At the present time, there is no recommendation regarding the early use of CPAP/PEEP in the delivery room and continuing it in the nursery for premature infants. However, some studies have suggested that the use of early CPAP/PEEP may be associated with improved outcomes such as: fewer babies needing to be placed on a breathing machine, less oxygen use in babies at one month of age and longer, and less need for a medication given in the babies lungs called surfactant. This study will begin in the delivery room and continue into the nursery to compare the use of CPAP/PEEP and early placement on the breathing machine along with the early use of surfactant to see if we can help lessen the severity of and even possibly prevent long term lung problems in premature infants.

Another part of the study will be looking at the ranges of oxygen saturation that are currently being used with premature infants. Doctors, nurses, and others taking care of your baby use a machine called a **pulse oximeter** in routine daily care to help them adjust the oxygen to meet the baby's needs. Sometimes higher ranges are used and sometimes lower ranges are used. All of them are acceptable ranges. In this part of the study, we would like to pinpoint the exact range that should be used to help prevent some of the problems that occur with premature babies such as Retinopathy of Prematurity (ROP). This is when there is abnormal blood vessel growth in the eye. It causes scar tissue to build up around the retina and if it pulls on the retina hard enough, it can cause blindness. It is known that ROP is increased by the prolonged use of supplemental oxygen from observations published in the 1950s, but the benefit of higher versus lower levels of oxygenation in infants, especially for premature infants, is not known. In going back and looking at how babies in the past were managed, it is being suggested that the use of lower saturation ranges may result in a lower incidence of severe ROP.

Expanation of Procedures

SUPPORT Study: If your baby is born before a gestational age of 28 weeks, he/she will randomly (like the flip of a coin) be placed into a group that receives early CPAP/PEEP use in the delivery room **or** early placement on the breathing machine (intubation) with the use of surfactant. Both ways are currently used in our hospital and we hope to determine which is the better way for these premature babies.

If your baby is in the Early CPAP group, he/she will be treated with CPAP/PEEP in the delivery room and will remain on it upon admission to the nursery. If, at any time, your baby shows signs of needing intubation for resuscitation purposes, then he/she will be intubated. If this happens within the first 48 hours he/she will also be given surfactant.

____
Parents' Initials or Those of
Legally Authorized Representative

If your baby is in the Early Surfactant and Ventilation group, he/she will be placed on the breathing machine in the delivery room and will be given surfactant within the first hour of birth. For the first 14 days of life, there will be guidelines for the doctors in the nursery to follow. These guidelines help them decide when to place babies on the breathing machines and when to try and take them off the breathing machines. These guidelines will also help decide when to put babies on and take them off of CPAP/PEEP.

The babies in this study will also be placed randomly (again, like the flip of a coin) into a group monitored with lower oxygen saturation ranges or higher oxygen saturation ranges. Oxygen saturation is measured on a baby with a machine called a pulse oximeter. It uses a tiny sensor on the hand or foot of the baby and can give the doctors a measurement of how saturated the baby's blood is with oxygen. Oximeters are not painful and can provide oxygen saturation measurements 24 hours a day. The babies in the lower range group will have a target saturation of 85-89%, while the babies in the higher range group will have a target saturation of 91-95%. All of these saturations are considered normal ranges for premature infants. If the saturation falls below 85% or goes higher than 95% then the pulse oximeter will alarm so that the doctors and nurses know when to turn your baby's oxygen up or down.

If your baby is still receiving oxygen close to the time of discharge (at approximately 36 weeks corrected age) a test will be done to determine the severity of lung disease that may be present. During this test, the oxygen your baby is receiving will be decreased gradually while continuously measuring oxygen saturation with the pulse oximeter. If the saturation falls below an acceptable range, your baby will then be returned to the prior oxygen level.

MRI Study: Part of your baby's regular care during the first few months after birth will include one or more head ultrasounds. The first one usually occurs during the first 2 weeks. There is also one done closer to the time of your baby's due date. In addition to the routine head ultrasound done close to your baby's due date, we would like to ask your permission to also do Magnetic Resonance Imaging (MRI) on your baby. The MRI is a common procedure that uses a magnetic field to make pictures of the inside of the head. It does this by taking a closer look at the tiny particles that are in the brain. Your baby will be placed on a narrow bed for about 20-30 minutes while the machine scans the brain and makes pictures. Your baby will not be exposed to any radiation when having the MRI done. The magnetic fields do not cause any known harmful effects at the levels used in the MRI machine. National and local guidelines have been developed for MRI machines, and these recommendations will be followed.

The ultrasound and MRI pictures of your baby's brain will be looked at by radiologists (doctors who are specialists in X-rays and other pictures of the body). Your doctors will tell you what they find. Because this study will be done in several hospitals across the United States, the ultrasound and MRI pictures from babies who participate will also be seen by other radiologists. They will look at all the pictures from all the babies.

Growth Study: It is routine care in the nursery to weigh and measure babies to watch their growth. With this secondary study to the SUPPORT Study, we will be collecting weight and measurements along with feeding information to take a closer look at how your baby grows.

____
Parents' Initials or Those of
Legally Authorized Representative

Duration of Study

Your baby will be involved in the ventilation part of this study for the first 14 days after birth. After the first 14 days, he/she will still be monitored with the saturation monitor as long as he/she is receiving extra oxygen. Once your baby has been off of oxygen for 72 hours, then the saturation monitor may be discontinued. Information will be gathered from the medical record throughout your baby's hospitalization.

We expect to include about 1310 babies in this study from all the NICHD Neonatal Research Network hospitals over a two year period.

Long Term Follow-up

When your baby is 18-22 months old, he/she will be seen in the Newborn Follow Up Clinic for an evaluation. At this visit, we will ask you a few extra questions about your baby's health. It is also possible that you may be contacted in the future for further long term follow up for the study.

Possible Benefits

The investigators do not promise or guarantee that your baby will receive any direct benefit from participating in the SUPPORT Study or any of the secondary studies. Participation will, however, benefit the medical community by providing valuable information which may help us treat babies in the future.

SUPPORT Study: If he/she is in the group which receives CPAP/PEEP, he/she might benefit by not needing additional breathing support. He/she may not require surfactant to be given into the lungs.

It is possible that using lower pulse oximeter ranges will result in fewer babies with severe Retinopathy of Prematurity (ROP).

MRI Study: There may be benefits to your baby directly, including findings of brain injury which will allow for earlier intervention than would normally occur.

Growth Study: There is no direct benefit to participating in this secondary study.

Possible Risks

SUPPORT Study: The possible risks of using CPAP/PEEP include stomach bloating and a temporary slowing of the heart rate. Another possible risk is collapsing one or both of the lungs. Use of the CPAP/PEEP at the level used in this study does not increase the risk of collapsed lungs.

Like with the use of CPAP/PEEP, a possible risk of being intubated (placed on the breathing machine) may include a temporary slowing of the heart rate or possibly the collapse of one or

Parents' Initials or Those of
Legally Authorized Representative

both lungs. Another risk is the possibility of the airway being punctured. Other possible risks include bruising or cutting of the tongue, gums, or airway.

Other potential risks during resuscitation after birth include; the need for chest compressions, rescue medications, and even death. It is not thought that the use of either of these ways of delivering oxygen to the baby's lungs increases these risks.

There is no known risk to your baby from monitoring with the pulse oximeters used for this study. The possible risk of skin breakdown at the site will be minimized by your baby's nurse moving the oximeter to another arm or leg a couple of times a day.

CPAP/PEEP, intubation, and pulse oximetry are commonly used in the newborn intensive care (NICU). Study participation should not increase these risks because all procedures are carried out by experienced NICU staff.

MRI Study: The risks of participating in this secondary study are minimal. The head ultrasound is a routine part of the care of a premature baby, and the way it is performed will not be changed for this study, nor does it cause any discomfort for the baby. The MRI is often done on babies whenever the doctor feels that it will give him information he needs to treat the baby. For this study, all participants who agree to participate will have an MRI done after getting the approval of the attending physician. The "tapping" noise that the MRI machine makes may agitate your baby. To minimize this, your baby's ears will be covered while the MRI is being done.

Your baby may also need to be given medicine to make him/her drowsy for the MRI. A possible risk of sedation is breathing difficulty. Your baby's heart rate and breathing will be closely monitored by an experienced baby nurse to reduce this risk.

Growth Study: There are no risks to participating in this secondary study.

<u>Alternative Procedures</u>

If you do not want your baby to participate in this study, he/she will receive the routine care given in the delivery room and nursery. The routine care may or may not include the use of CPAP and/or surfactant administration. He/she will most likely have oxygen saturation measured with a pulse oximeter as well. Routine care in the nursery may or may not include MRI.

<u>Confidentiality</u>

Information will be collected from your baby's chart by trained research personnel. It will be labeled with a code number and sent to the NICHD Neonatal Network's Data Collection Center at Research Triangle Institute (RTI) in North Carolina. The study log linking the code number to your baby's identity will be kept under lock and key in the UAB Division of Neonatology Research office. Any information that might identify your baby will not leave UAB. In addition, the NIH/NICHD, the UAB Institutional Review Board (IRB), the Food and Drug Administration (FDA), or the Office of Human Research Protections (OHRP) may monitor the

Parents' Initials or Those of
Legally Authorized Representative

trial records and the individual conducting the review may see your name in the file folder. Otherwise, the records will remain confidential to the extent permitted by law.

Information relating to this study, including your name, medical record number, date of birth and social security number may be shared with the billing office of UAB and UAB Health System-affiliated entities so that claims may be appropriately submitted to either the study sponsor or your insurance company for clinical services and procedures provided to you during the course of this study. The results of the treatment may be published for scientific purposes; however, your baby's identity will not be revealed. If you or your baby receive services in University Hospital, or The Children's Health System as part of this trial, this informed consent will be placed in and made part of your baby's permanent medical record at these facilities.

If your baby is transferred to another hospital or discharged before his/her eyes have reached maturity, then we will call the hospital or eye doctor to find out the results of eye exams that are done after discharge.

Withdrawal Without Prejudice

Participation in this study is voluntary. If you do not wish to participate in this study, your baby will not lose benefits to which he/she is entitled. You are free to withdraw your consent and to discontinue your baby's participation in this project at any time without prejudice against future medical care he/she may receive at this institution. This means that withdrawing him/her will have not effect on the future care or treatment of your baby by physicians or by this institution.

In addition, if the study physician feels that it is in your baby's best interest to be withdrawn from the study, he will do so immediately.

Significant New Findings

Any significant new findings discovered during the course of this study, which may influence your decision to allow your baby to continue participation, will be made known to you.

Costs of Participation

The cost of your baby's standard medical care, including surfactant administration and head ultrasounds, will be billed to you and/or your insurance company in the usual manner. The costs of the study, including the MRI that will be done close to your baby's due date, will be covered by a research grant. If any other MRI's are ordered by your baby's doctor as part of clinical care, they will be billed to you or your insurance company. There will be no additional cost to you or your insurance company for expenses related to this study.

Payment for Participating in Research

There will be no payment to you or your baby for participating in this research study.

Payment for Research Related Injuries

If, as a result of your baby's participation, he/she experiences injury from known or unknown risks of the research procedures as described, immediate care and treatment, including

____
Parents' Initials or Those of
Legally Authorized Representative

hospitalization if necessary, will be available. Neither UAB, The Children's Hospital of Alabama, nor the National Institutes of Health has made provision for monetary compensation in the event of injury resulting from the research, and in the event of such injury, treatment is provided, but is not free of charge. Further information regarding medical treatment can be obtained from Dr. Wally Carlo at 934-4680.

Questions

If you have questions about this study or experience any problems during the study, you should contact Dr. Wally Carlo at (205) 934-4680. You may also reach Monica Collins, RN, Shirley Cosby, RN, or Vivien Phillips, RN at (205) 934-5771. If you have questions about your baby's rights as a research participant, or concerns or complaints about the research, you may contact Ms. Sheila Moore. Ms. Moore is the Director of the Office of Institutional Review Board for Human Use (OIRB). Ms. Moore can be reached at (205) 934-3789 or 1-800-822-8816. If calling the toll-free number, press the press the option for "all other calls" or for an operator/attendant and ask for extension 4-3789. Regular hours for the Office of the IRB are 8:00 a.m. and 5:00 p.m. CT, Monday through Friday. You may also call this number in the event the research staff cannot be reached or you wish to talk to someone else.

Legal Rights

By signing this consent form, you are not waiving any of your or your child's legal rights.

Optional Participation in Secondary Studies

Please sign your choice below:

Neuroimaging and Neurodevelopmental Outcome (MRI Study)

_______________________ I agree to allow my baby to participate in the MRI Secondary Study.

_______________________ I **Do Not** agree to allow my baby to participate in the MRI Secondary Study.

Postnatal Growth of Infants enrolled in the SUPPORT Study (Growth Study)

_____________________ I agree to allow my baby to participate in the Growth Secondary Study.

_____________________ I **Do Not** agree to allow my baby to participate in the Growth Secondary Study.

____
Parents' Initials or Those of
Legally Authorized Representative

Signatures
You are making a voluntary decision whether or not to let your baby participate in this study. Your signature below indicates that you have decided to let your baby participate, that you have read (or been read) the information provided above, that you were given the opportunity to ask questions and that they have been answered to your satisfaction. The consent form will remain in the files at UAB Division of Neonatology and a copy will be placed in your baby's medical record. You will receive a copy of this signed consent form.

WAIVER OF ASSENT

The assent of ____________________ (name of child) has been waived because of age.

____________________________________ ______________________

Signature of Parent or Legally Authorized Representative — Date

____________________________________ ______________________

Signature of Person Obtaining Consent — Date

____________________________________ ______________________

Signature of Witness — Date

University of Alabama at Birmingham
Authorization for Use/Disclosure of Health Information for Research

What is the purpose of this form? You are being asked to sign this form so that UAB may use and release your health information for research. Participation in research is voluntary. If you choose to participate in the research, you must sign this form so that your health information may be used for the research.

Participant name: ___________________________

UAB IRB Protocol Number: *F040910010, F050922007 and X060418004*

Research Protocol: *The Surfactant Positive Airway Pressure and Pulse Oximetry Trial in Extremely Low Birthweight Infants; Secondary Studies: Neuroimaging and Neurodevelopmental Outcome and Postnatal Growth of Infants Enrolled in SUPPORT Study (Multicenter Network of Neonatal ICU's)*

Principal Investigator: *Wally Carlo, MD Namasivayam Ambalavanan, MD;*

Sponsor: *National Institute of Child Health and Development (NICHD)*

What health information do the researchers want to use? All medical information and personal identifiers; including past, present, and future history, examinations, laboratory results, imaging studies and reports and treatments of whatever kind related to or collected for use in the research protocol.

Why do the researchers want my health information? The researchers want to use your health information as part of the research protocol listed above and described to you in the Informed Consent document.

Who will disclose, use and/or receive my health information? The physicians, nurses and staff working on the research protocol (whether at UAB or elsewhere); other operating units of UAB, HSF, The Children's Hospital of Alabama, Callahan Eye Foundation Hospital and the Jefferson County Department of Public Health, as necessary for their operations; the IRB and its staff; the sponsor of the research and its employees; and outside regulatory agencies, such as the Food and Drug Administration.

How will my health information be protected once it is given to others? Your health information that is given to the study sponsor will remain private to the extent possible, even though the study sponsor is not required to follow the federal privacy laws. However, once your information is given to other organizations that are not required to follow federal privacy laws, we cannot assure that the information will remain protected.

How long will this Authorization last? Your authorization for the uses and disclosures described in this Authorization does not have an expiration date.

Can I cancel the Authorization? You may cancel this Authorization at any time by notifying the Director of the IRB, in writing, referencing the Research Protocol and IRB Protocol Number. If you cancel this Authorization, the study doctor and staff will not use any new health information for research. However, researchers may continue to use the health information that was provided before you cancelled your authorization.

Can I see my health information? You have a right to request to see your health information. However, to ensure the scientific integrity of the research, you will not be able to review the research information until after the research protocol has been completed.

Signature of parent or legally authorized representative ____________________________________ Date____________________

Printed Name of parent/participant's representative:____________________________________

Relationship to the participant:____________________________________

Project Title: The Surfactant Positive Airway Pressure and Pulse Oximetry Trial in Extremely Low Birth Weight Infants

A: The nature, duration and purpose of study.

You are being asked to give permission for your baby to participate in a research study designed to determine the best way to decrease the severity of the lung disease and eye disease common in premature babies by comparing two methods of treatment currently used in hospitals in the U.S. 1) Continuous Positive Airway Pressure or CPAP (the pressure used to keep the lungs inflated between breaths) after birth or 2) Ventilation (breathing machine) and *surfactant* (a liquid medication placed into the breathing tube) after birth.

We will also be studying the ranges of oxygen saturation that are currently being used with these same babies. You and your baby were selected as possible participants because you are less than 28 weeks pregnant and your baby may be born prematurely. The doctors at Women & Infants' Hospital along with 15 other centers across the country, are participating in this project sponsored by the National Institute of Child Health and Development. The duration of the study is until the infant is discontinued from oxygen; however, your infant will be followed throughout his/her hospital stay. In order to see if your infant's breathing improves as a result of the treatment received in this study we will ask you a few questions prior to your infant's discharge from the hospital and also at 6, 12 and 18-22 months corrected age. These questions will take about 10-20 minutes to complete.

If your baby is born prematurely he/she will be at risk for a breathing problem called **Respiratory Distress Syndrome (RDS).** A baby's lungs are made up of tiny lung sacs; each one is supposed to open and close as the baby breathes in and out. Oxygen is supposed to go in and carbon dioxide is supposed to come out. This works well in full term babies and adults. However, in premature babies, the lungs do not always work this way. Some lung sacs open and close normally and others collapse and stick together when the baby breathes out making it harder for the baby to breathe. Doctors treat this problem with expanding breaths and pressure to keep the lungs slightly inflated between those breaths. Keeping a little air pressure after the baby breathes out (inflating pressure) makes it easier for the baby to take the next breath.

After your baby is born, if he/she needs help breathing, the doctor or respiratory therapist may place a breathing bag over the baby's nose and mouth to provide oxygen and manual breaths. Sometimes a device called a Neopuff is used to force air into the baby's lungs. This inflating pressure is called Continuous Positive Airway Pressure or CPAP. Some hospitals use CPAP at birth and others use ventilation and surfactant. If a baby needs to be ventilated (placed on a breathing machine) *surfactant* is usually given through a breathing tube into the baby's windpipe to try to help keep the lung sacs expanded.

Another part of the study will be looking at the ranges of oxygen saturation that are currently being used with premature infants and how it affects their eyes. Doctors, nurses and others taking care of your baby use a machine called a pulse oximeter in routine daily care to help them adjust the oxygen to meet the baby's needs. Sometimes higher ranges are used and sometimes lower ranges are used. All of them are acceptable ranges used in different institutions. In this part of the study we would like to pinpoint the exact range that should be used to help prevent some of the problems that occur with premature babies such as Retinopathy of Prematurity (ROP). This is when there is abnormal blood vessel growth in the eye. It causes scar tissue to build up around the retina and if it pulls on the retina hard enough it can cause blindness. It is known that ROP is increased by prolonged use of supplemental oxygen. At the same time, not enough oxygen can affect growth and development. The study doctors are trying to find the best oxygen level to prevent lung and eye disease.

Women & Infants Hospital
IRB Approved
8·25·08
Expiration Date
8·24·09

B: The means by which it is conducted.

If your baby is born before a gestational age of 28 weeks, he/she will be randomly (like the toss of a coin) assigned to one of 4 groups:- 1) Early CPAP/Low oxygen saturation ranges, 2) Early CPAP/High oxygen saturation ranges, 3) Early Ventilation and surfactant /Low oxygen saturation ranges and 4) Early Ventilation and surfactant/High oxygen saturation ranges.

If your baby is assigned to early CPAP he/she will be treated with CPAP in the delivery room and will remain on it upon admission to the nursery. If, at any time your baby shows signs of needing intubation (a breathing tube in the windpipe) for breathing purposes, then he/she will be intubated. If this happens within the first 48 hours he/she will also be given surfactant.

If your baby is assigned to early Ventilation and surfactant he/she will be placed on a ventilator (breathing machine) in the delivery room and will be given surfactant into the lung within the first hour of birth.

For the first 14 days of life, there will be guidelines for the doctors in the nursery to follow. These guidelines help them decide when to place babies on the breathing machines and when to try and take them off the breathing machines. These guidelines will also help decide when to put them on and take them off of CPAP.

The babies in this study will also be randomly placed into a group monitored with lower oxygen saturations ranges or higher saturation ranges. Oxygen saturation is measured on a baby with a machine called a pulse oximeter. It uses a tiny sensor on the hand or foot of the baby and can give the doctors a measurement of how saturated the baby's blood is with oxygen. Oximeters are not painful and can provide oxygen saturation measurements 24 hours a day. The babies assigned to the lower oxygen saturation range will have a target saturation of 85%-89%, while the babies in the higher oxygen saturation range will have a target saturation of 91% to 95%. All of these saturations are considered normal ranges for premature infants.

The pulse oximeters used in this trial are FDA approved and have been modified for research purposes. This modification makes the monitors show a value which is either slightly higher or slightly lower than the true oxygen level when values are between 85% and 95%. Outside those ranges, the oximeter works the same as the standard of care device. If the saturation falls below 85% or goes higher than 95% then the pulse oximeter will alarm so that the doctors and nurses know when to turn your baby's oxygen up or down. This modification prevents the nursery staff from knowing which group your baby is in so that all infants are treated in an identical fashion.

If your baby is requiring oxygen therapy close to 36 weeks gestational age a test will be done to determine the severity of his or her lung disease. During this evaluation the oxygen given will be decreased gradually while continuously measuring the amount of oxygen in the blood using a standard pulse oximeter. If a saturation falls below an acceptable range, your child will be returned to the prior oxygen level.

You will also be contacted by a Follow –Up staff member at 6 and 12 months corrected age to ask you questions about your baby's breathing (especially coughing and wheezing), medication use, and visits to a doctor, Emergency Room, or Hospital for treatment of breathing problems. We will also ask you several questions about your family and yourself. As with all information we collect, the answers to these questions will be kept confidential and no names will be used.

Follow up at 18 months of age is essential for this study. The follow up visit will include a medical history, growth measurements, neurological exams including testing reflexes, developmental assessments and some questions about your baby's breathing and breathing symptoms similar to those asked at 6 and 12 months corrected age. This visit will take approximately 1.5 to 2 hours. Families who participate in this project are agreeing to remain in contact with the investigators and to return to the Neonatal Follow up Clinic with their child when he/she is 18 months of age. In order to successfully evaluate any eye disease your baby may develop the investigators will also want to follow up with your

Women & Infants Hospital
IRB Approved
8·25·08
Expiration Date
8·24·09

baby's eye doctor after discharge and obtain the results of your baby's eye exams until the eye results are final.

C: The possible benefit

The investigators do not promise or guarantee that your baby will receive direct benefit from being in the study. There may be benefits to your baby directly, including a possible decrease in chronic lung disease (need for oxygen near discharge) and/or a decrease in the need for eye surgery as a result of exposure to oxygen. Because we do not know in advance which treatment strategies are most effective, it is also possible that your baby will receive no direct benefit. However, as noted above, each of the 4 possible combinations of treatments is considered by some units to represent their desired approach.

D: The potential risks, and discomforts.

Because all of the treatments proposed in this study are standards of care at different hospitals across the country there is no predictable increase in risk for your baby. Infants randomized to the Early CPAP group may, at some point in their care, require intubation and assisted ventilation (methods to help them breathe). Participation in this study will not affect this decision if the attending physician deems it necessary. Some unknown risks may be learned during the study. If this occurs, you will be informed by the study personnel.

E: Alternatives

If you do not want your baby to participate in this study, he/she will receive routine care given in the delivery room and nursery which may or may not include the use of CPAP and/or surfactant administration. He/she will most likely have oxygen saturation measured with a pulse oximeter as well.

Women & Infants Hospital
IRB Approved
8.25.08
Expiration Date
8.24.09

Project Title: **The Surfactant Positive Airway Pressure and Pulse Oximetry Trial in Extremely Low Birth Weight Infants**

1. I have been told about this study. The experimental procedures have been discussed with me. I have had a chance to ask questions. My questions were answered to my satisfaction.

2. I authorize my confidential, protected health care information to be shared with individuals, persons and groups associated with this study. My confidential health care information will be used only as necessary to participate in this study. Except when required by law, I will not be identified in study records disclosed outside of Women & Infants' Hospital by name, social security number, address, telephone number, or any other direct personal identifier. For records disclosed outside of Women & Infants' Hospital the investigator or his staff will assign me a unique identifying code. The key to the code will be kept in a locked file in Dr. Laptook's research office.

3. I authorize as part of the study that Dr. Abbott Laptook and his study team) report the results of study related laboratory tests and x-rays to the study sponsor the National Institute of Child Health and Development(NICHD) and the Data Coordinating Center (Research Triangle Institute) . These would include laboratory tests such my baby's blood counts and tests to measure e.g; liver and kidney function and all x-rays or ultrasound, CT or MRI scans and the results of all my baby's eye exams. Information may have to be released when required by law when reasonable cause is shown under government regulations, or proper judicial orders. It may also be released to an official of the United States Food and Drug Administration, the United States Department of Health and Human Services, the United States Inspector General, the United States Office of Civil Rights, representatives of the NICHD and the Research Triangle Institute and the Women & Infants Hospital and its Institutional Review Board. If my research record is reviewed by any of these groups, they may also need to review my entire medical record. If disclosed by the sponsor, the information is no longer covered by the federal privacy regulations.

4. I authorize the retention of the study results in my child's research record (until the child reaches the age of 21). At the end of this retention period, either the research information not already in the child's record will be destroyed or information identifying the child will be removed from such study results. Any research information in the child's record will be kept indefinitely.

5. Any information from the study will be used for education or research purposes. My child's or my name will not be used.

6. I will be told of any changes to the risks or benefits of this study.

7. I do not have to take part in this study.
   I do not have to authorize use of my confidential, protected health information.
   My authorization to share my protected, personal health information expires after 21 years.

8. I am free to withdraw my consent at anytime. I am free to stop taking part in the study at any time. I will still receive the best care possible for me and/or my child. If I want to withdraw I should contact Dr. Abbott Laptook in writing and let him know I am withdrawing. His mailing address is Pediatric Suite, Women & Infants' Hospital, 101 Dudley Street, Providence, RI, 02905. If I withdraw my consent or permission the information which has already been collected about me or my child by the Hospital or the researchers will be kept by the researchers or hospital. This information may be needed to complete reports of this research.

Women & Infants Hospital
IRB Approved
8·25·08
Expiration Date
8·24·09

Project Title: **The Surfactant Positive Airway Pressure and Pulse Oximetry Trial in Extremely Low Birth Weight Infants**

9. In the event that injury occurs as result of this research, I am requested to notify the Principal Investigator Dr. Abbott Laptook at 401-274-1122, extension 1221.
   Should I be injured in a research project, treatment will be provided at Women & Infants Hospital, or at another appropriate health care institution, at no cost to me beyond that which third party payers will cover.
   Further information in regard to this may be obtained from Barbara Riter, Manager, Research Administration, whose telephone number is (401)-453-7677.

10. If I have questions about this study, I may call Dr. Abbott Laptook at 401-274-1122, extension 1221.If I have questions about my rights as a research subject, I may call Barbara Riter, Manager, Research Administration, at (401) 453-7677.

11. I will be given a copy of this signed consent form.

Future Contact: I have initialed whether I authorize the researchers to contact me:

_________I authorize the researchers to contact me in the future for research purposes.

Signature: ______________________________ Date: _____________ Time: _______ AM / PM

Name (please print):_________________________________

Name of Translator (if used): ________________

Translator's signature: ___________________

If not for self: relationship to patient: ______________________________

Person who explained study: ______________________ Date: _________

Hospital policy states that the signed original consent form is to be included in the subject's medical record. One copy of the original signed consent form is to be given to the subject. One copy of the signed original consent form should be retained in the investigator's files.

Women & Infants Hospital
IRB Approved
8·25·08
Expiration Date
8·24·09

UNIVERSITY HOSPITALS CASE MEDICAL CENTE[illegible]
CONSENT FOR INVESTIGATIONAL STUDIES

Project Title: SURFACTANT POSITIVE AIRWAY PRESSURE AND PULSE OXIMETRY IN EXTREMELY LOW BIRTHWEIGHT (ELBW) INFANTS- The SUPPORT Trial

Principal Investigator M. Walsh

Introduction

You have been admitted to MacDonald Women's Hospital and you are at risk for delivering your baby prematurely. If your baby is born early he/she may need help with his/her breathing immediately after birth in the delivery room and in the Neonatal Intensive Care Unit (NICU). Premature babies who need help with their breathing may also require extra amounts of oxygen. Many babies born before 28 weeks' gestation require this care.

You are being asked to allow your child to be considered for a research study will compare several ways which are normally used to help support breathing problems in infants who have premature lungs to see which is most helpful and least harmful. 1310 infants will be enrolled in this study.

You will need to be able to read and understand this consent form in order to allow your baby to be enrolled in this study. If you do not feel you are able to read and understand this consent, you should not sign to give your permission for your child to be enrolled in this study.

There are two common ways to help premature babies with breathing after birth. One common way is **CPAP** which stands for continuous positive airway pressure. A mask over the nose and mouth or soft nasal prongs placed into the tip of the nose may be used to provide air flow to help open the baby's lungs and give extra oxygen. Another common way is **Intubation** which is the placement of a breathing tube which is passed through the mouth into the airway and connected to a breathing machine (ventilator) to assist with respirations, give extra amounts of oxygen and give surfactant (a medication given into the lungs to help with breathing problems). Both ways are routinely used in the delivery room and in the NICU. The pediatric doctors, nurses and respiratory therapists will evaluate your baby's breathing immediately after birth and during his/her time in the NICU.

If a baby requires extra amounts of oxygen it is important that the concentration of oxygen in the blood (oxygen saturation) be monitored. A pulse oximeter is used to monitor the oxygen saturation. A sensor (the instrument that reads the oxygen saturation) is placed on the baby's hand, wrist or foot and is attached to the oximeter which then gives a continuous reading of the oxygen saturation.

It is important to keep the oxygen saturation in a certain range (85%-95%). Some doctors will want to keep the oxygen saturation level close to the upper part of the range. Some doctors prefer to keep the oxygen saturation close to the lower part of the range. Keeping the level in either end of the normal range is routinely used in the NICU for premature babies. If your baby requires extra amounts of oxygen when admitted to the NICU, an oximeter will be used in order to keep the oxygen within the normal range.

Project Title: SURFACTANT POSITIVE AIRWAY PRESSURE AND PULSE OXIMETRY IN EXTREMELY LOW BIRTHWEIGHT (ELBW) INFANTS- The SUPPORT Trial

Principal Investigator M. Walsh

Purpose

There are two purposes for this research study:

The first purpose of this study is compare either using a mask or soft nasal prongs to assist your baby with his/her breathing immediately after birth and continuing in the NICU **to** using a breathing tube, mechanical ventilation and receiving a drug called surfactant into the breathing tube. We want to compare these two to see if there is a difference in the amount of breathing help your baby requires and how long he/she will continue to need this help during the first two weeks of life.

The second purpose of this study is to compare babies who have oxygen saturations kept in the high end of the normal range with babies who have oxygen saturations kept in the low end of the normal range. The doctors, nurses and respiratory therapists will use a study pulse oximeter to monitor your baby during the time he/she requires extra amounts of oxygen.

Study Procedures

Your baby will receive care form the doctors, nurses and respiratory therapists who specialize in newborns to help stabilize him/her in the delivery room.

Your baby will be randomized (assigned by chance similar to a flip of a coin) to 2 treatment assignments.

The first assignment will determine his/her care in the delivery room as follows:
If your baby participates in this study he/she will be randomized (assigned by chance similar to a flip of a coin) to ***either*** the CPAP group ***or*** Intubation group to manage his/her breathing immediately after birth. This will determine if a mask/soft nasal prongs ***or*** a breathing tube will be offered first in the delivery room.

If your baby is randomized to the CPAP group a mask or soft nasal prongs will be used immediately after birth to assist your baby with his/her breathing in the delivery room and continued when he/she is admitted to the NICU. If your baby requires more help with his/her breathing a breathing tube and the ventilator may be offered and your baby will receive all routine care for premature infants with breathing problems. This may include giving a medication called surfactant into the lungs through the breathing tube to help with his/her breathing problems.

If your baby is randomized to the Intubation group your baby will have a breathing tube placed in the delivery room and will be admitted to the NICU. Your baby will receive all routine care for premature babies with breathing problems. This will include giving a medication called surfactant into the lungs through the breathing tube to help with his/her breathing problems.

Project Title: SURFACTANT POSITIVE AIRWAY PRESSURE AND PULSE OXIMETRY IN EXTREMELY LOW BIRTHWEIGHT (ELBW) INFANTS- The SUPPORT Trial

Principal Investigator M. Walsh

The second treatment assignment will determine which end of the normal oxygen saturation range (high or low end) as follows:

When your baby is admitted to the NICU, he/she will be randomized (assigned by chance similar to the flip of a coin) to be kept in a certain oxygen saturation range. This will determine if your baby will have his/her oxygen saturation level kept in the high ***or*** low part of the normal oxygen saturation range. The oximeter will be used for as long as your baby is requiring oxygen and until he/she has been in room air (no extra oxygen) for at least 3 days. Your baby will also have his/her oxygen monitored by other routine methods used in the NICU, which may include blood samples to measure the oxygen concentration when the doctors feel it is necessary.

Your infant will have al usual care for infants born before 28 weeks gestation. This includes measuring your child's weight several times weekly, as well as, measuring length and head circumference once each week. If your child is not on a breathing tube or CPAP, your child's length will be performed using a length board [laced inside his/her bed. The length board will remain in your child's bed only long enough to obtain the length measurement.

Usual care for premature infants in the NICU includes head ultrasound examinations to look at the brain. The head ultrasound is convenient, can be done at the bedside and does not require sedation. Routine ultrasounds are done during the first two weeks of life and again close to 36 weeks' corrected age or more often if the doctors feel it is necessary.

Another examination of the brain called an MRI (magnetic resonance imaging) can also give information about your child's brain. The doctors would like to compare results of the routine head ultrasounds to an MRI done close to 36 weeks' corrected age for infants who were treated with CPAP or Intubation and assigned to the high or low end of the normal oxygen saturation range. We will compare these results to see which test will best predict neuromotor (physical abilities such as walking, talking, vision and hearing) and neurodevelopmental (development of intelligence and language) outcome at 18-22 months' corrected age.

Your infant will be taken to the MRI department in a special transporter that provides a protective environment including warmth, oxygen, breathing assistance and I fluids if necessary. Most often infants at this age will sleep during this test and will tolerate the MRI well. If your child does not sleep during the brain MRI, your child may need a mild sedative. If this is necessary, the doctors and nurses will monitor your child's response to the mild sedative.

Follow-up Procedures

In order to understand problems that some former premature infants experience after discharge, we conduct parent interviews in person and by telephone. The interviews ask about breathing problems, especially wheezing and the need for visits to the doctor and/or the hospital for breathing problems. We want to compare infants who are treated with CPAP or Intubation and who are assigned to the high

or low end of the normal oxygen saturation range to better understand breathing problems during early childhood.

The first interview will be conducted in the hospital close to the time your child is scheduled for discharge. We will ask you questions about your home and whether breathing problems run in your family. The next three interviews will be conducted by telephone at 6, 12, and 18 months' corrected age. During each telephone interview we will ask about your child's breathing, especially wheezing and coughing and about your child's need for medical visits and treatments for breathing problems. We also ask how your child is adjusting to his/her new home. These telephone interviews will be scheduled at a time that is convenient for you.

Risks

Possible complications with using soft nasal prongs and/or the ventilator may include trauma to the airway, collapse of airway passages, abnormal lung damage by air getting into the tissues or lung collapse. However, the need for help with breathing may be necessary due to the premature birth.

The oxygen saturation ranges to be used are currently used for usual care in premature infants in the NICU. The known risks associated with the high end of the normal oxygen saturation range may include slow or abnormal growth of blood vessels in the eye causing vision problems. Known risks associated with the low end of the normal oxygen saturation range may include low amounts of oxygen delivered to the tissues.

Risks associated with the use of surfactant may include a temporary drop in the oxygen level during the time the medication is given, brief breathing difficulty while the surfactant is being given and bleeding from the lungs.

Premature babies who have lung problems have a risk of long term breathing problems which may require extra amounts of oxygen.

Risks associated with the MRI examination may include minor skin irritation from the tape used to apply monitoring electrodes (sensors that monitor vital signs during the MRI). If sedation is needed, risks may include less vigorous breathing and lower blood pressure which may require additional monitoring. An intravenous line (IV) may also be required to administer sedation, and if needed, the risk of an IV may include bruising, swelling or rarely an infection. The transport of your child to the MRI department may also represent a possible risk. However, only those patients considered stable for transport will have an MRI performed.

There are no known risks associated with head ultrasounds done in the NICU.

We anticipate no risk to you, your child and your family to participate in interviews in person and by telephone about continued breathing problems in former premature infants.

IRB NUMBER: 11-04-29
IRB APPROVAL DATE: 08/15/2011
IRB EXPIRATION DATE: 06/15/2012

There may be unforeseen risks associated with participation in this research study that are not known at this time.

Benefits

There may be no direct benefit to your child to participate in this research study.

Your child's participation in this study may aid in the understanding of the use of a mask/nasal prongs or a breathing tube to help premature babies with breathing immediately after birth and in the NICU. It will also help the doctors to learn more about premature infants managed in the high and low ends of the normal range of oxygen saturation.

An MRI performed for the study may have potential benefit which may include a more detailed view of your child's brain that could show abnormalities not seen with a head ultrasound. This would allow the early identification of possible problems for each individual child.

There will be no benefit to you, your child or your family for participation in the parent interviews. However, information from this study may determine the effect of the different ways to help premature babies with breathing and managing oxygen saturation levels on breathing problems in early childhood.

Participation

You may choose for your baby to have the MRI performed to look at the structure of his/her brain and help identify injury.

Yes, I would like my child to have an MRI performed for this study.* ____________________
Parent initials/ date

No, I do not want my child to have an MRI performed for this study.* ____________________
Parent initials/ date

You may choose to participate in the parent interviews before your child's discharge and at 6, 12, and 18 months' corrected age.

Yes, I would like to participate in the parent interviews.* ____________________
Parent initials/ date

No, I do not want to participate in the parent interviews.* ____________________
Parent initials/ date

*Your signature will also be required on the last page of this consent form.

Early Withdrawal From the Study

You may choose to withdraw your child from this research study for any reason and at any time with no penalty or loss of benefit of care. Your child will continue to receive all routine care for premature infants which may include help with breathing, monitoring with a pulse oximeter, head ultrasound examinations and weekly measurements to monitor growth.

Alternatives to Participation

If you do not want your child to participate in this study he/she will not be randomized to soft nasal prongs or a breathing tube to assist with breathing immediately after birth in the delivery room and after admission to the NICU. Also, he/she will not be assigned to the high or low end of the normal oxygen saturation range.

All routine and usual care will be provided to your baby which may include help with his/her breathing in the delivery room and in the NICU. The doctors may use nasal prongs, a breathing tube and the ventilator and the oximeter to monitor my baby's oxygen saturation when they feel it is necessary. Usual care for premature infants includes head ultrasounds. An MRI test may be performed if the doctors feel it is necessary.

Financial Information

There will be no extra cost to you or your insurance company for your child's participation in this research study. You and your insurance company will be responsible for all usual care in the NICU which may include the use of nasal prongs, a breathing tube and the ventilator to assist with your child's breathing, surfactant to help with breathing problems and an oximeter to monitor oxygen saturation.

There is no additional cost to you or your insurance company and your insurance company will not be billed for the MRI done for the study. You and your insurance company will be responsible for all usual care which may which may include head ultrasounds or brain MRI when the doctors feel it necessary.

There is not cost to you or your insurance company for participation in the parent interview prior to discharge and at 6, 12, and 18 months' corrected age of your child. You and your child will not be paid for participation in this research study.

Confidentiality

Information collected for this study will be sent electronically to a central data center via a dedicated computer which is password sensitive. Your child will be identified by a study number and not by name or other identifying information.

If it is necessary for the study personnel to contact you or your child at a later time, we will contact you at the address and/or telephone numbers you have provided. In the event that we cannot locate you at the address and/or telephone numbers you have provided, we may use other information in your medical record such as social security numbers, your child's pediatrician or other contact information.

Summary of your rights as a participant in a research study

Your participation in this research study is voluntary. Refusing to participate will not alter your usual health care or involve any penalty or loss of benefits to which you are otherwise entitled. If you decide to join the study, you may withdraw at any time and for any reason without penalty or loss of benefits. If information generated from this study is published or presented, your identity will not be revealed. In the event new information becomes available that may affect the risks or benefits associated with this study or your willingness to participate in it, you will be notified so that you can decide whether or not to continue participating. If you experience physical injury or illness as a result of participating in this research study, medical care is available at University Hospitals Case Medical Center (UHCMC) or elsewhere; however, UHCMC has no plans to provide free care or compensation for lost wages.

Disclosure of your study records

Efforts will be made to keep the personal information in your research record private and confidential, but absolute confidentiality cannot be guaranteed. The University Hospitals Case Medical Center Institutional Review Board may review your study records. If this study is regulated by the Food and Drug Administration (FDA), there is a possibility that the FDA might inspect your records. In addition, for treatment studies, the study sponsor and possibly foreign regulatory agencies may also review your records. If your records are reviewed your identity could become known.

Contact information

_M.C. Walsh, A.A. Fanaroff, N. Newman, B. Siner__ has described to you what is going to be done, the risks, hazards, and benefits involved. The researchers conducting this study are __M.C. Walsh, N. Newman and B. Siner. You may ask any questions you have now. If you have any questions, concerns or complaints about the study in the future, you may contact them at _216-844-3387_.
If the researchers cannot be reached, or if you would like to talk to someone other than the researcher(s) about; concerns regarding the study; research participant's rights; research- related injury; or other human subject issues, please contact University Hospitals Case Medical Center's Chief Medical Officer at (216) 844-3695 or write to:
The Chief Medical Officer, The Center for Clinical Research, University Hospitals Case Medical Center, 11100 Euclid Avenue, Lakeside 1400, Cleveland, Ohio, 44106-7061.

Project Title: SURFACTANT POSITIVE AIRWAY PRESSURE AND PULSE OXIMETRY IN EXTREMELY LOW BIRTHWEIGHT (ELBW) INFANTS- The SUPPORT Trial

Principal Investigator M. Walsh

Signature

Signing below indicates that you have been informed about the research study in which you voluntarily agree to participate; that you have asked any questions about the study that you may have; and that the information given to you has permitted you to make a fully informed and free decision about your participation in the study. By signing this consent form, you do not waive any legal rights, and the investigator(s) or sponsor(s) are not relieved of any liability they may have. A copy of this consent form will be provided to you.

____
Printed Name of Participant

________________________________ Date________ ____________________________
Parent or Legal Guardian signature Relationship to Child

________________________________ Date________ ____________________________
Signature of Person Obtaining Consent Printed Name of Person Obtaining Consent
(Must be study investigator or individual who has been designated in the Checklist to obtain consent.)

________________________________ Date________ ____________________________
Second Parent signature Relationship to Child

________________________________ Date________ ____________________________
Signature of Person Obtaining Consent Printed Name of Person Obtaining Consent
(Must be study investigator or individual who has been designated in the Checklist to obtain consent.)

If only one parent can sign this consent, indicate the reason that applies to the other parent.

( ) deceased
( ) unknown
( ) legally incompetent
( ) no legal responsibility for the care and custody of the child
( ) not reasonably available - indicate why _______________________________
(acceptable reasons for this category must not be based on convenience)

________________________________ Date________ ____________________________
Signature of Principal Investigator Printed Name of Principal Investigator
(Affirming subject eligibility for the Study and that informed consent has been obtained.)

Project Title: SURFACTANT POSITIVE AIRWAY PRESSURE AND PULSE OXIMETRY TRIAL IN EXTREMELY LOW BIRTHWEIGHT INFANTS- The SUPPORT Trial

Principal Investigator: M.C. Walsh

Consent signed and copy returned to parent/family. Date_______________

Name of Person returning consent______________________________

The University of Texas Southwestern Medical Center at Dallas
Parkland Health & Hospital System and Children's Medical Center

CONSENT TO PARTICIPATE IN RESEARCH

Title of Research: The Surfactant Positive Airway Pressure and Pulse Oximetry Trial in Extremely Low Birth Weight Infants

Sponsor: National Institute of Child Health and Human Development Neonatal Research Network

Investigators:	Telephone No. (regular office hours)	Telephone No. (other times)
Pablo J. Sánchez, MD	214-648-3753	972-206-9021
Nancy Miller, RN	214-648-3780	214-590-6500
Melissa Leps, RN	214-648-3780	214-590-6500
Roy Heyne, M.D.	214-648-3753	972-206-8791
Janet Morgan, RN	214-456-2585	972-206-9153
Andrea Duncan, MD	214-648-2098	972-451-1192
Arpitha Chiruvolu	214-648-2098	972-451-1193
Darryl Miao, MD	214-648-2098	972-206-9268
LaKeitha Foster, MD	214-648-2098	972-326-1689
Liaqat Khan, MD	214-648-2098	972-601-0011
David Lam, MD	214-648-2098	972-326-1690
Catrinel Marinescu, MD	214-648-2098	972-326-1691
Lilian St. John, MD	214-648-2098	972-206-8775
Nathan Sundgren, MD	214-648-2098	972-601-8701
Nimisha Gupta, MD	214-648-2098	972-326-1175

INVITATION: You/your newborn infant are invited to participate in this research because there is a possibility he/she will be born 12 to 16 weeks early and could possibly develop a lung problem called bronchopulmonary dysplasia (an abnormal formation of the lungs in premature infants leading to a need for oxygen treatment for a long time) and an eye problem called retinopathy of prematurity (an eye disease that may result in poor vision or loss of sight in premature infants), which may be caused by high oxygen treatment.

Medical research involves offering a plan of care to a group of patients, collecting and studying information about each patient's experience, and using that information to develop the best possible care for future patients.

NUMBER OF PARTICIPANTS: The sponsor plans to include 1300 newborn infants in the study from all the National Institute of Child Health and Human Development Neonatal Research Network hospitals over a two-year period.

IRB File # 012005-019
Approved: MAR 24 2010
Expires: DEC 20 2010

PURPOSE: This study has three purposes and they are:

1) To compare newborn infants, in the delivery room, who receive breathing support through their nose with a cannula with newborn infants who have a tube placed in their windpipe (a process called intubation) and surfactant (a liquid which helps babies with immature lungs breathe easier by keeping their lungs from collapsing) given in the first hour of life.

2) To compare newborn infants, in the intensive care nursery, treated with low range (85-89%) oxygen saturation levels (a measure of the amount of oxygen in the blood) with newborn infants treated with high range oxygen saturation (91-95%) levels.

3) To measure the effects of the oxygen therapies used in the study on the growth of premature infants.

PROCEDURES

Breathing support through the nose with a cannula blowing air (also called nasal continuous positive airway pressure), or intubation with surfactant are both treatments currently used in the delivery room at Parkland Health and Hospital System. The decision to use one or the other on any newborn infant is made by the doctor who is in the delivery room.

Screening: The study doctor will ask you questions on whether you are admitted to the hospital in premature labor and whether we can approach you to be in this study.

Randomization:

If you agree for your newborn infant to be in this study, your infant will be randomized (chosen by chance like the flip of a coin) to one of two lung treatment groups:

1) Nasal continuous positive airway pressure through the nose or the mouth in the delivery room immediately after birth and continuing in the intensive care nursery, or

2) The placement of a tube in his/her windpipe in the delivery room followed by giving surfactant and mechanical ventilation (breathing for the baby using a machine).

After admission to the intensive care nursery, your newborn infant will also be randomized to a low or a high oxygen saturation group using a specially designed oximeter (a monitor that displays blood oxygen level).

Your infant will have a 1 in 4 chance of being in one of these groups:

- Group 1: the nasal continuous positive airway pressure /low saturation,
- Group 2: the nasal continuous positive airway pressure /high saturation,
- Group 3: oral intubation with surfactant/low saturation, or
- Group 4: oral intubation with surfactant/ high saturation.

Treatment:

Groups 1 and 2 (nasal continuous positive airway pressure):

Delivery Room Care: In the delivery room the doctor resuscitating your newborn infant will try using nasal continuous positive airway pressure on your baby. If your baby responds to nasal continuous positive airway pressure, he/she will continue on nasal continuous positive airway pressure and will be transferred to the intensive care nursery. If your baby does not improve with nasal continuous

IRB File # 012005-019
Approved: MAR 24 2010
Expires: DEC 20 2010

positive airway pressure, the doctor resuscitating your baby will intubate your baby in the delivery room. If intubated in the delivery room, your infant will receive one dose of surfactant within one hour of life. Your infant may receive more than one dose of surfactant in his/her first day of life, but your infant's doctor, based on your infant's condition, will decide this.

Care in the Neonatal Intensive Care Nursery: The doctor may intubate your infant if he/she believes your infant is not doing well on the nasal continuous positive airway pressure. The study sponsor has guidelines for your infant intubation or extubation (the process of removing the tube from the windpipe) to safeguard your infant. Your infant's doctor will follow the sponsor guidelines for intubating or extubating your infant in the first two weeks of life. However, if your infant requires intubation three times, he/she will be out of the study and further intubations or extubations will be decided solely by your infant's doctor. After two weeks of life all intubations and extubations are decided only by your infant's doctor.

Groups 3 and 4 (oral intubation with surfactant):
Delivery Room Care: In the delivery room, the doctor resuscitating your newborn infant will place a tube in his/her windpipe after delivery and will give a dose of surfactant in the first hour of life. Your infant will be transported to the intensive care nursery on a breathing machine.

Care in the Neonatal Intensive Care Nursery: Your newborn infant will be admitted to the intensive care nursery on a breathing machine. The study sponsor has specified guidelines for the first two weeks of life at which your infant will be taken off the breathing machine and your infant's doctor will follow those criteria to decide on extubating your infant. Once your infant is extubated for the first time, further intubations or extubations will be decided only by his/her doctor.

All study infants will be placed on the study oximeter within two hours of birth. (An oximeter is an object placed on the skin to measure oxygen in the blood). Which oxygen saturation group your infant is randomized to will not be known to the nurse taking care of your infant, or his/her doctor. Only the study coordinator will know which group your infant is in. However, your infant will either be on the high end or the low end of the normal oxygen saturation that we normally use in our intensive care nursery. Your infant will remain on the study oximeter until he/she reaches 36 weeks adjusted age (e.g. 24 wks gestation plus 12 wks of age = 36 wks adjusted age) or until he/she is discharged home.

We will measure your infant's weight, length, and head at birth, day of life 7, 14, 21, 28, and at 32 and 36 wks of age and at home discharge to evaluate the effects of the study procedures on his/her growth.

All other care will be conducted as normal during your infant's participation in the study. The studies to be done on your baby's blood will be performed on blood already drawn in the standard care and no additional blood draws will be done for the research. Your baby will be followed in our infant follow-up clinic at Children's Medical Center after discharge from the intensive care nursery as we usually do for all babies his/her size.

Follow-up after discharge from the Neonatal Intensive Care Nursery:

At the time of a regular follow-up visit, following your infant's discharge from the NICU, our follow-up staff (Dr. Roy Heyne or his designee) will interview you (or your designee) to find out about your child's diet, breathing problems in the family, and things in the home that may increase your child's risk of breathing problems. This interview will take about 15 minutes and will include questions about the air quality at your home, your home location, your infant's exposure to infections, your family

IRB File # 012005-019
Approved: MAR 24 2010
Expires: DEC 20 2010

history of asthma and allergies, and any recent hospital or doctor office visits. You do not need to answer any questions that make you uncomfortable. The follow-up staff will be in touch with you and your infant, either by telephone or in person at one of your follow-up visits every 6 months for a total of three times. At these times, they will ask questions about your child's breathing (especially wheezing and coughing), medication use, and visits to a Doctor, Emergency Room, or Hospital for treatment of breathing problems. They will also ask you several questions about things in the home or day care setting that may affect your child's breathing. The entire call should take about 15 minutes of your time, less if your baby has had no breathing problems. If your infant is followed in the Low Birth Weight Clinic at Children's Medical Center, the follow-up staff will arrange to meet you during the clinic visit to ask you these questions. Otherwise, they will schedule the telephone calls at a time that is convenient for you. The telephone calls will occur when your infant is 6, 12, and 18 months after his/her expected delivery at full term. The results from your baby's questionnaire will be combined with those of other infants from around the country. However, your baby's name will not be used.

At 18-22 months of age your baby will receive, at no charge, a complete exam of their muscles, nerves, intelligence and motor function. We will also measure his/her weight, length, and head size. This exam is done at the Children's Medical Center Follow-up Clinic.

Evaluations During the Research:
Dr. Sanchez and his research staff will closely monitor your infant's response to the study in the delivery room and in the intensive care nursery. Dr. Sanchez and his research staff will review your infant's medical record to gather information on your infant's date, time and place of birth, delivery room events, birth weight, length, head circumference, age at birth, gender, results of lab tests performed by your infant's doctor, results of the physical exam performed by your infant's doctor, type and name of medicines your infant receives during his hospitalization, duration and type of respiratory support and mechanical ventilation, duration and degree of oxygen therapy, complications during hospitalization, amounts of milk and intravenous nutrition, duration of hospital stay, results of the head ultrasounds performed during the hospital stay (head ultrasounds, also called head sonograms, are pictures taken of the baby's brain by a special machine that is brought to the baby's bedside), results of the eye exam performed during the hospital stay, results of the neurologic exams and the Bayley tests performed in the follow-up clinic(the neurologic and Bayley tests are a complete evaluation of your infant's muscles, nerves, intelligence, and motor function). Dr. Roy Heyne and his research staff will review the results of the interview to gather information on your infant breathing problems and conditions that may affect his/her breathing following the study.

Investigational Procedures:
The oximeters (oxygen monitors) used in this trial are FDA approved oximeters, but have been modified for this study. The FDA is a government agency that oversees new drugs and medical equipment.

POSSIBLE RISKS
All the treatments in this study are currently used in the intensive care nursery and most infants born at the same age as your infant will receive all those treatments during their stay in the intensive care nursery.

The risk of intubation includes injury to your infant's throat and windpipe, but this is unusual. The risk of surfactant treatment includes bleeding in the lung but this is rare. Infants receiving nasal continuous positive airway pressure in the delivery room may have problems breathing and their heart-beat may abnormally slow down. If this happens to your infant, your infant's doctor in the delivery room will intubate your infant and place him/her on a breathing machine. Infants placed on nasal continuous

IRB File # 012005-019
Approved: MAR 24 2010
Expires: DEC 20 2010

positive airway pressure for a long time, may have damage to their nose; our nurses and doctors are very aware of this possible problem and are careful to prevent it. The weight, length, and head measurements of your infant are performed by experienced nurses and do not add any risk. Some unknown risks may be learned during the study. If these occur, you will be informed by the study personnel.

Following discharge from the NICU, you (or your designee) will be involved in a series of interviews about factors related to your child's breathing; and while answering interview questions you may experience some anxiety or emotional discomfort; but you do not have to answer questions you do not feel comfortable responding to.

Another risk of this study is the risk to confidentiality. Every effort will be made to keep your infant's medical record confidential. There will be no name or other patient identification in any study report that may be published after the study is completed. Measures taken to protect you and your infant's identity are described in the confidentiality section.

Blood samples: Your infant will have the same amount of blood collected whether your infant receives standard medical care for your infant's health problems or participates in this research. Therefore, your infant's risk of complications from collecting the blood is the same.

Your infant may experience discomfort, bleeding and/or bruising. On a rare occasion, an infection could develop at the site where the blood was collected.

Unforeseen risks: A previously unknown problem could result from your newborn infant's participation in this research. It is not possible to estimate the changes of such problems or how serious problems could be.

POSSIBLE BENEFITS

Benefit to your infant: Your study doctor cannot guarantee that your newborn infant will benefit from participation in this research.

Benefit to other premature infants: The information learned from this study may help us better treat premature infants in the future. However, your infant's study doctor will not know whether there are benefits to other premature infants until all of the information obtained from this research has been collected and analyzed.

ALTERNATIVES TO YOUR INFANT'S PARTICIPATION IN THIS RESEARCH: Your infant does not have to participate in this research to receive care for your infant's medical problem. If you decide not to participate in this research, your infant will receive the standard of care at Parkland Health and Hospital System and Children's Medical Center intensive care nurseries. The standard of care at the Parkland Health and Hospital System and Children's Medical Center neonatal intensive care nurseries varies with the attending doctor taking care of your infant and may be similar to any of the above 4 groups of therapies that the research is studying.

Please ask your infant's study doctor as many questions as you wish. The doctor's answers to your questions could help you decide whether your infant will participate in this research or receive the standard care that is currently available for your infant's medical problem.

If you decide now that your infant will participate in research, and later change your mind, your infant may stop participation in the research then and receive the standard of care.

IRB File # 012005-019
Approved: MAR 24 2010
Expires: DEC 20 2010

THE STUDY DOCTOR'S DECISION TO STOP YOUR INFANT'S PARTICIPATION: Your infant's study doctor or the sponsor may stop your infant's participation in this research without your or your infant's permission under any one of the following conditions:

- Your infant's study doctor believes participation in the research is no longer safe for your infant.
- Your infant's study doctor believes that other treatments may be more helpful.
- The sponsor or the FDA stops the research for the safety of the participants.
- The sponsor cancels the research.

PROCEDURES AFTER STOPPING PARTICIPATION IN THIS RESEARCH: If you, the study doctor, or the sponsor stops your infant's participation in the research, it is your responsibility to do the following:

- Let the study doctor know immediately that you wish that your infant withdraw from the research.
- Return to the research center for tests that may be needed for your infant's safety.
- Discuss your infant's future medical care with the study doctor and/or your infant's regular doctor.

INCENTIVE TO TAKE PART IN THIS RESEARCH: You will not be paid for your infant's participation in this research.

COSTS TO YOU: The sponsor will pay the expenses for the neurodevelopmental testing (tests that evaluate the function of nerves, muscles and intelligence of your infant) that is part of the research.

Expenses related to standard medical care for prematurity are your responsibility (or the responsibility of your insurance provider or government program). Since nasal continuous positive airway pressure, intubation, mechanical ventilation, surfactant treatment, and the use of a pulse oximeter are all part of the routine care of preterm infants, the research will not pay for those therapies.

You, your insurance or your government program will be responsible for the cost of delivery room care, the costs of the day-to-day care in the intensive care nursery, the using cost of all respiratory equipments (mechanical ventilation machine, nasal continuous positive airway machine, pulse oximeter) used in the day-to-day care of your infant, and the costs of the daily physician care.

COMPENSATION FOR INJURY: Compensation for an injury resulting from your infant's participation in this research is not available from the University of Texas Southwestern Medical Center at Dallas, Children's Medical Center, or Parkland Health & Hospital System. You and your infant retain your legal rights during your infant's participation in this research.

VOLUNTARY PARTICIPATION IN RESEARCH: You and your infant have the right to agree to or refuse participation in this research. If you decide that your infant will participate and later change your mind, you are free to discontinue participation in the research at any time.

Your refusal to participate in this research will involve no penalty or loss of benefits to which you and

IRB File # 012005-019
Approved: MAR 24 2010
Expires: DEC 20 2010

your infant are otherwise entitled. Your refusal to participate in this research will not affect your legal rights or the quality of health care that you and your infant receive at this center.

NEW INFORMATION: Any new information which becomes available during your infant's participation in the research and may affect your infant's health and safety or your willingness for your infant to continue in the research will be given to you.

RECORDS OF YOUR INFANT'S PARTICIPATION IN THIS RESEARCH: You and your infant have the right to privacy. Any information about you or your infant that is collected for this research will remain confidential as required by law. In addition to this consent form, you will be asked to sign an "Authorization for Use and Disclosure of Protected Health Information for Research Purposes."

YOUR QUESTIONS: Your infant's study doctor, Dr. Pablo J. Sánchez, or his research nurse, Nancy Miller, RN, are available to answer your questions about this research; and Dr. Roy Heyne and his research nurse, Janet Morgan, RN, are available to answer questions about the breathing interview. The Chairman of the IRB is available to answer questions about your rights and your infant's rights as a participant in research or to answer your questions about an injury or other complication resulting from your infant's participation in this research. You may telephone the Chairman of the IRB during regular office hours at 214-648-3060.

YOU WILL HAVE A COPY OF THIS CONSENT FORM TO KEEP.

Your signature below certifies the following:

- You have read (or been read) the information provided above.
- You have received answers to all of your questions.
- You have freely decided that your infant may participate in this research.
- You understand that you and your infant are not giving up any of your legal rights.

____
Participant's Name (printed)

____
Legally authorized representative's name (printed)

______________________________ ______________
Legally authorized representative's signature Date

____
Legally authorized representative's name (printed)

______________________________ ______________
Legally authorized representative's signature Date

IRB File # 012005-019
Approved: MAR 24 2010
Expires: DEC 20 2010

Name of person obtaining consent (printed)

Signature of person obtaining consent

Date

IRB File # 012005-019

Approved: MAR 24 2010

Expires: DEC 20 2010

THE SURFACTANT POSITIVE AIRWAY PRESSURES AND PULSE OXIMETRY TRIAL IN EXTREMELY LOW BIRTH WEIGHT INFANTS

The SUPPORT Trial of the NICHD Neonatal Research Network

INVITATION TO TAKE PART

You are invited to enter your infant into a research study conducted at the University of Texas Health Science Center and Memorial Hermann Children's Hospital (MHCH). You are being asked to allow your infant to be in the study because there is a possibility he/she will be born between 16 and 12 weeks early (24-27 weeks gestational age).

Dr. Kathleen Kennedy and Dr. Jon Tyson (doctors who specialize in the care of sick or premature infants) and the National Institutes for Child Health and Human Development (NICHD) Neonatal Research Network are conducting this research study. Your decision to allow your infant to take part is voluntary and may be withdrawn at any time. Refusal to take part in this study or withdrawal at a later date will not affect the care given to your infant by the doctors or any other health professionals. You may refuse to answer any questions asked or written on any forms. This study has been approved by the Committee for the Protection of Human Subjects (CPHS) for the University of Texas Medical School at Houston as HSC-MS-04-415. The CPHS is an independent committee to help make sure that research studies are safe and properly performed.

DESCRIPTION OF RESEARCH

Purpose:
In the delivery room here at MHCH, most infants that are less than 28 weeks gestation routinely have a breathing tube placed and receive a dose of surfactant (a medicine which helps infants with immature lungs breath easier by helping keep their lungs from collapsing). Upon admission into the Neonatal Intensive Care Unit, they are placed on a breathing machine or ventilator which delivers oxygen and mechanical breaths. Additional doses of surfactant may also be given to these infants during the first two days of life. As many as half of the infants that receive this treatment go on to have a significant problem called Bronchopulmonary Dysplasia (BPD). BPD is caused by prematurity and long term exposure to the ventilator. A number of studies have suggested that the use of early CPAP (pressure applied with a face mask/prongs to help keep the lungs inflated) may result in improved outcomes, including a decreased need for mechanical ventilation, a decreased need for surfactant therapy, and a decrease in the amount of extra oxygen the infant requires.

Although additional oxygen is very necessary to the survival of premature babies with very under-developed lungs, studies have shown that too much oxygen can also be harmful. One of the problems that may occur in these tiny premature infants is retinopathy of prematurity or ROP. This eye disease may result in impairment of vision or even blindness and is associated with excessive oxygen therapy. We do not know at what blood oxygen levels ROP occurs.
Growth is another concern for the doctors who care for your infant. Infants that do not have enough oxygen grow poorly however infants that have too much oxygen are predisposed to a breathing problem called brochopulmonary dysplasia (BPD) which has been linked to poor growth.
It is also thought that high amounts of oxygen may actually cause damage to the lungs that could result in impaired growth of the airways. This could predispose infants to having increased respiratory problems during and after hospitalization.

Your infant's doctors have to balance the benefit and risk of using oxygen. We follow the oxygen level in the blood with a machine called an Oxygenation Saturation Monitor. The higher the monitor is reading the more oxygen is in the infant's blood. The highest a monitor can read is 100%. We keep our saturation monitors between 85% and 95% for our premature infants. Studies have suggested that the use of lower saturation ranges may result in a lower risk of severe ROP and BPD. However, oxygen saturations which are too low may result in poor neurological outcomes such as learning disabilities or uncoordinated muscle movements. Currently there is no agreement on the accepted saturation monitor ranges for managing premature infants from birth.

The purposes of this trial are the following:

1) To compare infants who receive delivery room CPAP and who have strict guidelines for having a breathing tube placed with infants who have the tube placed and surfactant given in the delivery room to determine if the use of CPAP from birth results in a decrease in broncopulmonary dysplasia. Broncopulmonary dysplasia or BPD happens

when the lungs grows abnormally because of exposure to high doses of oxygen and mechanical ventilation (breathing machine).

2) To compare low range (85-89%) oxygen saturation levels with high range (91-95%) levels to determine if a lower range results in a decrease in ROP.

3) To compare growth from birth to 18 months adjusted age in the high versus low saturation groups.

4) To compare breathing outcomes at discharge to 18 months adjusted age in the high versus low saturation groups.

DESCRIPTION OF THE STUDY

Procedure:
If you agree for your infant to take part in this study, the following will happen to him/her: If you consent to take part in the study and your infant delivers between 24 and 27 6/7 weeks gestation he/she will be randomized (chosen by chance like the flip of a coin) at the delivery to one of two treatment strategies. At the same time your infant will also be randomized to a "high" or "low" oxygen saturation monitor group. Please note that if you deliver twins, triplets etc they will all be randomized to the same treatment group and oxygen saturation group.
The treatments are as follows:
1) CPAP in the delivery room immediately after birth and continuing in the NICU, or
2) The placement of a tube in his/her trachea in the delivery room followed by a dose of surfactant and ventilation (breathing for the infant using a machine).
We will know if your infant was randomized to receive the breathing tube or CPAP.

The oxygen saturation monitor your infant has been randomized to will be started after he/she gets to the Neonatal ICU. He/she will be place on either a "High" reading or "Low" reading saturation monitor. Within the range of oxygen which we normally use, your infant will either be on the high end of normal or the low end of normal. We will not know what saturation monitor your infant gets. The saturation monitors used in this trial are FDA approved saturation monitors which have been modified for research purposes. This modification makes the monitors show a value which is either slightly higher or slightly lower than the true oxygen level when values are between 85 and 95%. Outside those ranges, the saturation monitor works the same as other oxygen saturation monitors.

Usually all infants in the nursery are weighed daily. Each week as part of routine care your infant's measurements (height and head circumference) are done. The research staff will be doing these measurements at age 1, 2, 3, and 4 weeks, at 32 weeks and 36 weeks adjusted age and at discharge.

To assess your infant's breathing outcomes from discharge until 18 months adjusted age 4 interviews/questionnaires will be conducted by phone or in person in the follow-up clinic.

If you decide to sign this consent and let your infant be in this study, we will collect information from your infant's medical record about his/her medical care and how he/she does throughout his/her hospital stay.

OPTIONAL STUDY COMPARING MRI AND HEAD ULTRASOUND

Premature infants, like yours, have a higher risk for brain injury. Although the way we will be providing breathing support (breathing machine or CPAP) and oxygen are routinely use in our nursery, the doctors in the Neonatal ICU (and those doing the study) do not know if either method could cause brain injury. For this reason a sub-study has been added. It is also not known if the brain ultrasound or the MRI is better at predicting your infant's outcome. Both have been used as part of routine care in neonatal units to help counsel parents and better predict the outcome of high risk infants like yours. You may decide whether you agree to allow your infant to take part in this sub-study by checking one of the boxes below.
Part of your infant's routine care during the first few weeks after birth will include one or more head ultrasounds. Head ultrasounds are done at your infant's bedside and take less than 15 minutes to complete. Also, within about four weeks of your infant's planned due-date, a MRI of the brain is done as part of his/her routine care. The ultrasounds and MRI studies show images (pictures) of the brain which are used to look for brain injury. Infant's who take part in this study will have an extra head ultrasound at the time the MRI is done so the doctors doing the study can compare the findings and see if one way of imaging gives better information than the other. This is another question this sub-study will be able to answer. There are no additional risks, time commitments, or costs to you if you allow your infant to take part in this sub-study. Because your infant would receive both a brain ultrasound and a MRI, you can be sure that he/she is receiving the best diagnostic tests available. His/her doctors

would know the result of both tests which may improve his/her ability to evaluate what your infant's development is likely to be.
Place an X in the box to indicate if you agree or do not agree to allow your infant to take part in this sub-study. If you decide not to take part in the sub-study you can still take part in the main study.

☐ Yes, I agree to allow my infant to take part in the sub-study

☐ No, I do not agree to allow my infant to take part in the sub-study

TIME COMMITMENT

This study begins in the delivery room when your infant is born and if he/she is between 24 0/7 to 27 6/7 weeks gestation. The CPAP/breathing tube part of the study will continue for 14 days. Your infant will remain on the saturation monitor until he/she reaches 36 weeks adjusted age. (e.g. 24 wks gestation plus 12 weeks of age = 36 weeks adjusted age.)
Your infant will be followed in our Infant Follow-up clinic at 6 and 12 months as standard of care for small infant. These visits will take approximately two hours. At 18-22 months corrected age your infant will receive a complete exam. A neonatologist or pediatrician will do a complete physical exam. Also a developmental specialist will do an assessment of his/her muscle movements and learning skills. On this day the evaluation will take approximately 3 hours. Again, this follow-up visit is considered standard of care for small infants.
The breathing outcome interviews will be conducted prior to discharge, and at 6, 12 and 18 months adjusted age. These interviews will be conducted by phone or in the clinic. The interview/questionnaire will be asking about doctor visits, hospitalizations or emergency room visits, medications, diet, and a breathing problems assessment. These interviews should take 15-20 minutes each.

BENEFITS

There may or may not be a benefit to your infant for taking part in this study. Possible benefits to your infant include; a possible decrease in brochopulmonary dysplasia (need for extra oxygen near discharge) and/or a decrease in the need for eye surgery as a result of exposure to oxygen. Because we do not know in advance the actual strategies chosen for your infant, or which of the treatment strategies is the most effective, it is also possible that your infant will receive no direct benefit. The knowledge learned from this study may help us treat infants in the future. However, each of the 4 possible combinations of treatments is currently used by some NICUs as their primary approach to treating premature infants.

RISKS AND/OR DISCOMFORTS

Taking part in a study may involve some added risks or discomforts. Because all of the treatments proposed in this study are standard of care, there is no expected increase in risk for your infant.

Infants randomized to the CPAP group may, at some point in their care, require a breathing tube and ventilation. If the attending doctor deems this necessary, being part of this study will not affect this decision. Some unknown risks may be learned during the study. If these occur, you will be informed by the study personnel.

ALTERNATIVES

This study is voluntary. If you choose not to have your infant take part in the project, he/she will continue to receive routine care.

STUDY WITHDRAWAL

You may withdraw your infant from the study at any time by contacting one of the Research Team at 713-500-5734.

COSTS, REIMBURSEMENTAND COMPENSATION

There will be no additional costs to you or your infant for taking part in this research study

IN CASE OF INJURY

If your infant suffers an injury as a result of taking part in this research study, please understand that nothing has been arranged to provide free treatment of the injury or any other type of payment. However, all needed facilities, emergency treatment, and professional services will be available to your infant, just as they are to the community in

general. You should report any injury to Dr. Kathleen Kennedy at 713-500-5734 and to the Committee for the Protection of Human Subjects at 713-500-7943.

CONFIDENTIALITY

You, or your infant, will not be personally identified in any reports or publications that may result from this study. Any personal information about your infant that is gathered during this study will remain confidential to every extent or the law. A special number will be used to identify your infant in the study and only the investigator(s) at UT Medical School and Hermann Children's Hospital will know your infant's name. Please understand that representatives of the Food and Drug Administration (FDA), the Committee for the Protection of Human Subjects (CPHS), and the sponsor of this research may review your infant's research and/or medical records for the purposes of verifying research data. The FDA representative can see personal identifiers in your infant's record; however his/her identity will be shielded from the view of the sponsor's representative. Your infant's name will not be seen by the sponsor unless the CPHS gives its permission for you to be contacted. You or your infant will not be personally identified in any reports or publications that may result from this study. There is a separate authorization form that you will be asked to sign which details the use and disclosure of your infant's protected health information.

QUESTIONS

You are making a decision whether or not to voluntarily take part in this study. You should not sign until you understand all the information presented in the previous pages and until all your questions about the research have been answered to your satisfaction. If you have any additional questions regarding this study Dr. Kathleen Kennedy or one of the research nurses will be available to answer them for you at any time. You may contact them at 713-500-5734 or 713-704-2900.

SIGNATURES

Sign below only if you understand the information given to you about the research and choose to take part. Make sure that any questions have been answered and that you understand the study. If you have any questions or concerns about your rights as a research subject, call the Committee for the Protection of Human Subjects at 713-500-7943. If you decide for your infant to take part in this research study, a copy of this document will be given to you.

______________________	__________	__________
Parent/guardian signature	Date	Time
______________________		
Printed name of Parent/guardian		
______________________	__________	__________
Person obtaining consent	Date	Time
______________________		
Printed name of person obtaining consent		
______________________	__________	__________
Witness	Date	Time

This study (HSC-MS-04-415) has been reviewed by the Committee for the Protection of Human Subjects (CPHS) for the University of Texas Houston Health Science Center. For any inquiries regarding research subject's rights, or to report any research related injury, call the CPHS at (713) 500-7943.

IUPUI and CLARIAN INFORMED CONSENT STATEMENT FOR

The SUrfactant Positive Airway Pressure and Pulse Oximetry Trial in Extremely Low Birth Weight Infants

STUDY PURPOSE:

You are invited to have your baby participate in a research study looking at two ways in which babies are treated for breathing problems. Your baby was selected as a possible participant because you are less than 28 weeks pregnant and your baby may be born early. Most babies born this early need some type of help to breathe. Doctors have two different ways they use to help these small babies breathe. Some doctors insert a breathing tube down into your baby's lungs, give a medication called surfactant, and attach a machine to the breathing tube that will breathe for your baby. This machine is called a ventilator or respirator. Other doctors use a special piece of equipment called continuous positive airway pressure, or CPAP, that is placed over the baby's nose and mouth and applies air pressure thru the mouth and nose into the baby's lungs. With CPAP, the baby is breathing on his/her own; some babies who are placed on CPAP eventually get tired and need to placed on a respirator. This study is trying to determine if using CPAP versus using a ventilator and surfactant immediately after birth will help decrease the severity of lung disease in babies who are born early. This study is also looking at the ranges of oxygen saturation that are currently being used with premature babies. Doctors, nurses, and others taking care of your baby use a machine called a pulse oximeter in routine daily care to help them adjust the oxygen to meet the baby's needs. Sometimes higher ranges are used and sometimes lower ranges are used. All of them are acceptable ranges. In this part of the study, we would like to pinpoint the exact range that should be used to help prevent some of the problems that occur with premature babies such as eye disease, called Retinopathy of Prematurity (ROP). This eye disease happens when there is unusual growth of blood vessels in the eye. It causes scar tissue to build up around the retina and if it pulls on the retina hard enough it can cause blindness. It is known that ROP is increased by the prolonged use of extra oxygen. The benefit of higher versus lower levels of oxygenation in babies, especially premature babies, is not known. This study hopes to determine what specific range is the best. Another goal of the study is to (delete also) compare brain imaging by ultrasound and magnetic resonance imaging (MRI), done around the time when your infant would have been born at full-term, to determine if one method of imaging gives more useful information that the other. The results will then be compared to the physical and developmental exams your infant will have when he/she is 18-22 months old and may help to determine which oxygen range is best for premature babies. Finally, to determine the effect of the SUPPORT Study treatment on your infant's breathing health, phone interviews will be conducted after your infant's discharge from the hospital. During these interviews, you will be asked questions about whether your baby has experienced any breathing difficulties since being discharged from the hospital.

NUMBER OF PEOPLE TAKING PART IN THE STUDY:

If you agree to participate, your baby will be one of 1310 babies who will be participating in this research nationally, and one of 150 babies locally.

PROCEDURE FOR THE STUDY:

If you agree for your baby to be in the study, your baby will be treated in one of four ways. If your baby is born before you reach your 28th week of pregnancy he/she will randomly (like a flip of a coin) be placed into a group that receives CPAP use in the delivery room or into a group that receives placement of the breathing tube, medication (surfactant), and then placed on the breathing machine. Both ways are currently used in our hospitals and we hope to find out which is the better way for these tiny babies.

If your baby is in the CPAP group, he/she will be treated with CPAP immediately after birth and will remain on it upon admission to the nursery. If your baby shows signs of needing the breathing tube and machine at any time, then your baby's doctor will provide this for him/her. If this happens within the first 48 hours of life your baby will also receive the surfactant medication.

If your baby is in the breathing tube/medication group, he/she will have the tube placed and will be placed on the breathing machine in the delivery room and will be given the surfactant medication with the first hour after birth.

For your baby's first 14 days of life, there will be guidelines for the doctors in the intensive care nursery to follow. These guidelines help them decide when to place babies on the breathing machines and when to try and take them off the breathing machines. These guidelines also will help decide when to put them on or take them off of CPAP. These guidelines have been agreed upon by all the doctors taking care of your baby.

The babies in this study will also be placed randomly (again, like a flip of the coin) into a group monitored with lower oxygen saturation ranges or higher oxygen saturation ranges. Oxygen saturation is measured on a baby with a machine called a pulse oximeter. It uses a tiny sensor on the hand or foot of the baby and can give the doctors a measurement of how much oxygen is in the baby's blood. Oximeters are not painful and can provide oxygen saturation measurements 24 hours a day. The babies in the lower range group will have a target saturation of 85-89%, while the babies in the higher range group will have a target range of 91-95%. All of these saturations are considered normal ranges for premature babies. If the saturation falls below 85% or higher than 95% then the pulse oximeter will alarm so that the doctors and nurses know when to turn your baby's oxygen up or down.

Your baby will be involved in the CPAP or breathing tube part of the study for the first 14 days after his/her birth. After the first 14 days, he/she will still be monitored with the saturation monitor as long as he/she is receiving extra oxygen. Once your baby has been off of oxygen for 72 hours, then the saturation monitor may be discontinued. Information will be gathered from the medical record once your baby has been discharged from the hospital. When your baby is 18-22 months old, he/she will be seen in the Newborn Follow-up Clinic for an evaluation. This evaluation will include a complete examination of the muscles, nerves, and mental and motor skills. This examination will be given by specially trained developmental pediatricians and psychologists who are experts at providing care for former premature babies. After this evaluation, your baby's participation in this study will end.

Part of your infant's regular care during the first few months after birth will include one or more head ultrasounds to check for brain injury, including at about the time he/she would normally have been born. If you decide to allow your infant to be in this study, he/she will also have a brain MRI and another head ultrasound within about a month of his/her intended due date. MRI is a method of making pictures of both normal and abnormal changes within the body. It is based on the relationship between atoms within the body when placed inside a large magnet. MRI uses a magnetic field to make images of the inside of the body. Your infant will be placed on a long narrow couch for 20 to 30 minutes while the machine gathers data. During this time, your infant will not be exposed to radiation or X-rays but, rather, a strong magnetic field and radiofrequency magnetic fields. Your baby will not feel either. Your baby will, however, hear repetitive tapping noises that arise from the gradient coils of the MRI machine. We will provide earmuffs that your baby will wear. Generally, infants who are fed, wrapped in warm blankets, and have earmuffs in place, sleep through MRIs. The ultrasound and MRI pictures of your baby's brain will be looked at by radiologists at your baby's hospital (doctors who are specialists in X-rays and other pictures of the body). Your doctors will tell you what they find. Because this is a study that involves babies from many hospitals across the United States, all of the ultrasound and MRI pictures of the brain from all the babies who participate in this study will also be seen by other radiologists who will look at all the pictures from all the babies.

Many children who were born prematurely and needed help to breathe continue to have breathing problems such as wheezing and coughing in the first two years of life. We would like to stay in touch with you by telephone when your baby goes home and continuing every 6 months for a total of four times. At these times, we will ask questions about your child's breathing, medication use, and visits to a doctor, emergency room or hospital for treatment of breathing problems. We will also ask several questions about you and your family. Each call should take about 15 minutes. You do not need to answer any questions that make you uncomfortable.

The results from your baby's questionnaire will be combined with other infants from around the country. However, your infant's name will not be used.

RISKS OF TAKING PART IN THE STUDY:

The possible risks of using CPAP include stomach bloating and a temporary slowing of the heart rate. Another possible risk is collapsing one or both of the lungs. Use of the CPAP at the level used in this study does not increase the risk of collapsed lungs. These risks are present during the routine use of CPAP; involvement in this study does not increase these risks.

Like with the use of CPAP, a possible risk of placing a breathing tube and machine may include a temporary slowing of the heart rate or possibly the collapse of one or both lungs. Another risk is the possibility of the airway being punctured. Other possible risks include bruising or cutting of the tongue, gums or airway. Again, these risks are present during routine use of the breathing tube and machine. Involvement in this study does not increase these risks.

Other potential risks during the time immediately after birth include the need for chest compressions, rescue medications, and even death. The use of either of these procedures (CPAP or the breathing tube) will not increase these risks.

Pulse oximeters are used routinely in thousands of intensive care nurseries all across the country every day. There is no known risk to your baby from monitoring with the pulse oximeters used for this study. The possible risk of skin breakdown at the site will be minimized by your baby's nurse, moving the sensor to another arm or foot a few times a day. The pulse oximeter will be used for your baby whether or not your baby is in the study.

All of the imaging proposed in this study is within standard of care; there is no predictable increase in risk for your baby. Some unknown risks may be learned during the study. You will be told of any new information that is learned which may affect your child's condition or influence your willingness to have him/her continue participation in this study. The only other risk of this study is risk to confidentiality. Every effort will be made to keep your child's medical record confidential.

BENEFITS OF TAKING PART IN THE STUDY:

There may or may not be any extra benefit to your baby taking part in this study. It is possible that group that receives CPAP might benefit by not needing additional breathing support. He/she may not require the surfactant medication. It is also possible that using the lower part of the normal saturation ranges will result in fewer babies with severe eye problems.

All infants in the study may benefit from the MRI that is conducted. Infants may benefit if they detect brain injury, which will allow for earlier intervention than would normally occur. If is also possible that your baby will receive no direct benefit. The knowledge learned from this study may help us treat babies in the future.

ALTERNATIVES TO TAKING PART IN THE STUDY:

If you do not want your baby to participate in this study, he/she will receive the routine care for premature infants. This may or may not include use of CPAP, the breathing tube/machine and the surfactant medication. He/she will also have oxygen saturation measured by a pulse oximeter in the ranges of 85-95% as well.

CONFIDENTIALITY:
Efforts will be made to keep your baby's personal information confidential. We cannot guarantee absolute confidentiality. Your baby's personal information may be disclosed if required by law. Your baby's identity will be held in confidence in reports in which the study may be published.

Organizations that may inspect and/or copy your baby's research records for quality assurance and data analysis include groups such as the investigator and his/her research associates, the IUPUI/Clarian Institutional Review Board or its designees, the National Institutes of Health (NIH) and the data coordinating center, Research Triangle Institute.

COSTS/COMPENSATION:

Taking part in this study will not lead to added costs to you or your baby's insurance company. The cost of the MRI and head ultrasound procedures for the sole purpose of research is paid for by the National Institute of Health (NIH), the study sponsor.

You will not receive payment for taking part in this study.

In the event of physical injury resulting from your baby's participation in this research, necessary medical treatment will be provided to your baby and billed as part of your baby's medical expenses. Costs not covered by your baby's health care insurer will be your responsibility. Also, it is your responsibility to determine the extent of your baby's health care coverage. There is no program in place for other monetary compensation for such injuries. However, your baby is not giving up any legal rights or benefits to which your baby is otherwise entitled.

CONTACTS FOR QUESTIONS OR PROBLEMS:

For questions about the study or a research-related injury, contact the researcher Brenda B. Poindexter MD at 317-274-4716. If you cannot reach the researcher during regular business hours (i.e. 8:00AM-5:00PM), please call the IUPUI/Clarian Research Compliance Administration office at 317/278-3458 or 800/696-2949. After business hours, please call the neonatologist on call at 317-274-6648.

In the event of an emergency, you may contact the neonatologist on-call at 317-274-6648.

For questions about your baby's rights as a research participant or complaints about a research study, contact the IUPUI/Clarian Research Compliance Administration office at 317/278-3458 or 800/696-2949.

VOLUNTARY NATURE OF STUDY:

Taking part in this study is voluntary. You may choose for your baby not to take part or your baby may leave the study at any time. Leaving the study will not result in any penalty or loss of benefits to which your baby is entitled.

SUBJECT'S CONSENT:

In consideration of all of the above, I give my consent for my baby to participate in this research study.

I acknowledge receipt of a copy of this informed consent statement.

BABY'S NAME:______________________________________ Date:______________

SIGNATURE OF PARENT:________________________________ Date:______________

SIGNATURE OF PARENT:________________________________ Date:______________

SIGNATURE OF PERSON OBTAINING CONSENT:________________ Date:______________

IRB Approval Date: DEC 4 2007

Continuing Review Due: SEP 1 9 2008

OR IRB USE ONLY
APPROVED BY: IRB-01
IRB ID #: 200605740
APPROVAL DATE: 05/19/08
EXPIRATION DATE: 05/19/09

INFORMED CONSENT DOCUMENT

Project Title: **The Surfactant Positive Airway Pressure and Pulse Oximetry Trial in Extremely Low Birth Weight Infants**

Research Team: **Edward Bell, MD**
Michael Acarregui, MD
Gretchen Cress, BSN, RN
Karen Johnson, BSN, RN
Laura Knosp, BSN, RN
Nancy Krutzfield, MSN
Ruthann Schrock, BSN
John Widness, MD
Sara Scott, BSN, RN

This consent form describes the research study to help you decide if you want to participate and allow your child to participate. This form provides important information about what you will be asked to do during the study, about the risks and benefits of the study, and about your rights as a research subject.

- If you have any questions about or do not understand something in this form, you should ask the research team for more information.
- You should discuss your participation with anyone you choose such as family or friends.
- Do not sign this form unless the study research team has answered your questions and you decide that you want to be part of this study.
- Your decision will not affect your infant's right to medical care that is not research-related.

WHAT IS THE PURPOSE OF THIS STUDY?

This is a research study. We are inviting you and your child to participate in this research study because there is a possibility that he/she will be born 12-16 weeks early (at 24 to 28 weeks of pregnancy).

There are 5 purposes of this research study:

- The first is to compare two ways of helping babies who were born prematurely and have difficulty breathing. One way is by using a mask or tube in the baby's nose, called CPAP (continuous positive airway pressure) to assist the baby with his/her breathing immediately after birth and continuing in the NICU. The second way is using a breathing tube in the baby's windpipe, mechanical ventilation and a drug called surfactant, which is given into the breathing tube. We will compare these two to see if there is a difference in the amount of breathing help the babies require and how long they will continue to need this help during the first two weeks of life.
- The second purpose is to compare babies who have oxygen saturations kept in the high end of the normal range with babies who have oxygen saturations kept in the low end of the normal range.
- The third purpose is to compare brain imaging by ultrasound and magnetic resonance imaging (MRI), done around the time when a baby would normally be born, to determine if one method of imaging gives more useful information than the other.

- The fourth purpose is to compare outcomes of babies in the two oxygen saturation ranges after they go home, particularly in regards to any wheezing or chronic coughing they might have.
- The fifth purpose is to compare outcomes of babies in the two oxygen saturation ranges after they go home, particularly in regards to their growth.

HOW MANY PEOPLE WILL PARTICIPATE?

Approximately 75 babies and their mothers will take part in this study at the University of Iowa. This study is being conducted at 15 other centers, and the total number of babies and mothers in all the centers combined will be approximately 1300.

HOW LONG WILL I BE IN THIS STUDY?

If you agree for yourself and your baby to take part in this study, your involvement will last until your baby is 18-22 months old.

WHAT WILL HAPPEN DURING THIS STUDY?

A few minutes before your baby is born, (s)he will be randomly assigned, like the flip of a coin, to either the “CPAP” group or “Intubation” group to manage his/her breathing immediately after birth.

The first assignment will determine his/her care in the delivery room as follows:
If your baby is randomized to the CPAP group, a mask or tube in the nose will be used immediately after birth to assist your baby with his/her breathing in the delivery room and continued when he/she is admitted to the NICU. If your baby requires more help with his/her breathing, a breathing tube will be placed in the windpipe and the ventilator used. Your baby will receive all routine care for premature babies with breathing problems. This may include giving a medication called surfactant into the lungs through the breathing tube.
If your baby is randomized to the Intubation group, your baby will have a breathing tube placed in the windpipe in the delivery room and will be admitted to the NICU. Your baby will receive all routine care for premature babies with breathing problems. This will include giving a medication called surfactant into the lungs through the breathing tube.

The second treatment assignment will determine which end of the normal oxygen saturation range (high or low end) will be used as follows:
When your baby is admitted to the NICU, he/she will be randomized (assigned by chance similar to the flip of a coin) to be kept in a certain oxygen saturation range. This will determine if your baby will have his/her oxygen saturation level kept in the high or low part of the normal oxygen saturation range. The study oximeter (blood oxygen monitor) will be used for as long as your baby is requiring oxygen and until he/she has been in room air (no extra oxygen) for at least 3 days. We don’t know which level is best for premature infants and that is why we’re doing the study.

Other aspects of your infant’s care will be the standard treatments for premature babies in the UIHC

NICU.

All babies in the study will have the following:

We will measure your baby's weight (using the scale routinely used), length (using a special board to measure premature babies), and head circumference (using the paper measuring tape routinely used) when he/she is 7, 14, 21 and 28 days old, at the time your baby is 32 weeks gestation (about 8 weeks before his/her due date), at the time your baby is 36 weeks gestation (about 4 weeks before his/her due date) and right before he/she is discharged.
All babies born prematurely at UIHC routinely have ultrasounds of their heads. Your baby will have the routine ultrasounds of his/her head as well as a test called a MRI (Magnetic Resonance Imaging) study. The ultrasound is a painless procedure where a probe is placed on your baby's soft spot and a picture is made by sound waves. The MRI is a specialized brain scan that takes detailed pictures of the brain structure and can detect normal and abnormal brain tissue. It is very likely that we will need to sedate your baby (giving medication to help relax him/her) during the procedure. The MRI will be done around the time when your baby would normally have been born. Your baby will be swaddled and monitored throughout the procedure.

All babies who participate in the project will return to the UIHC High Risk Infant Follow-up Clinic at regular intervals during the first two years. When the children enrolled in this study return for their 18-22 month old assessments of growth and development, the study will collect information at that visit. The information collected will include who takes care of your baby, what their marital status is, what their income and medical insurance are, who lives in the household with the baby, the baby's medical history, and the results of the physical exam and developmental testing.

To learn about the breathing outcomes of babies in the study, we will interview you before your baby is discharged from the hospital. At this interview, we will ask you questions about your family, including questions about family history of breathing problems, and questions about your home, including things that may increase your child's risk of breathing problems. The interview will take about 15 minutes.

We will continue to stay in touch with you and your infant by telephone or in person at one of your visits every 6 months over the next 18-22 months, a total of three times. At these times, we will ask questions about your child's breathing (especially wheezing and coughing), medication use, and visits to a Doctor, Emergency Room, or Hospital visits for treatment of breathing problems. We will also ask you several questions about your family and yourself. You do not need to answer any questions that make you uncomfortable. The entire call should take about 15 minutes of your time, less if your baby has had no breathing problems.

We will schedule the telephone calls at a time that is convenient for you. The telephone calls will occur when your infant is 6, 12, and 18 months after his/her expected delivery at full term.

OR IRB USE ONLY
APPROVED BY: IRB-01
IRB ID #: 200605740
APPROVAL DATE: 05/19/08
EXPIRATION DATE: 05/19/09

The following chart is a summary of what will happen and when:

**At Delivery**	**Days of Life 7, 14, 21, 28, 32 weeks, 36 weeks**
CPAP or Intubation with Surfactant	Measurements (weight, length, head)
Higher or lower oxygen saturation assignment<br>Ends at 14 days of life	
**35 to 42 weeks gestation (around "term")**	**At discharge**
Head ultrasound and MRI	Measurements (weight, length, head)
**6 months of age, 12 months of age**	**18-22 months of age**
Interview (by phone or in person)	Interview (by phone or in person)<br>High Risk Infant Follow-up Clinic visit

<u>WHAT ARE THE RISKS OF THIS STUDY?</u>

There may be some risks from being in this study. The use of CPAP and intubation in managing infants' breathing are within usual standard of care at UIHC NICU. CPAP and intubation with ventilation have the risk of trauma to the airway, abnormal lung damage by air getting into the tissues, and the possibility that the tube may cause air to get into the stomach.

During the brain MRI we will need to attach monitors to your infant to keep track of his or her heart rate and respiration. The tape we use to attach the electrodes may cause temporary minor skin irritation The MRI makes a loud, banging noise while it is taking pictures. A set of special earmuffs will be placed over your child's ears to help with the noise. There are no known harmful effects from exposure to magnetic fields (MRI). However, some patients undergoing this procedure become anxious. If sedation is necessary, risks from the medication used include decreased blood pressure and the possibility of breathing difficulty. Your baby's heart rate and respiration will be closely monitored.

Some of the interview questions may make you uncomfortable. You may choose not to answer any or all of the questions

<u>Are there any Unforeseen Risks?</u>

In addition to the risks described above, there may be unknown risks, or risks that we did not anticipate, associated with being in this study.

WHAT ARE THE BENEFITS OF THIS STUDY?

We don't know if you or your baby will benefit from being in this study.

However, we hope that, in the future, other people might benefit from this study because the knowledge learned may help in the understanding of the use of CPAP or a breathing tube to help premature babies with breathing immediately after birth and in the NICU. It may also help us to learn more about premature infants managed in the high and low ends of the normal range of oxygen saturation. We may also gain knowledge about using MRI to detect brain injuries. Finally, knowledge may be gained about the effects of CPAP and oxygen on breathing and growth outcomes of premature infants.

WHAT OTHER TREATMENT OPTIONS ARE THERE?

Before you decide whether or not you want your baby to be in this study, your doctor will discuss the other options that are available to you. Instead of being in this study, you could choose to have your baby treated in the routine way, which may include help with his/her breathing in the delivery room and NICU using the mask, tube in the nose or breathing tube in the windpipe with a ventilator, surfactant and an oximeter to monitor oxygen saturations.

WILL IT COST ME ANYTHING TO BE IN THIS STUDY?

You and your baby will not have any additional costs for being in this research study.

You and/or your medical/hospital insurance carrier will remain responsible for your and your baby's regular medical care expenses.

WILL I BE PAID FOR PARTICIPATING?

You and your baby will not be paid for being in this research study.

WHO IS FUNDING THIS STUDY?

The National Institute of Health, NICHD Neonatal Research Network is funding this research study. This means that the University of Iowa is receiving payments from The National Institute of Health, NICHD Neonatal Research Network to support the activities that are required to conduct the study. No one on the research team will receive a direct payment or increase in salary from The National Institute of Health, NICHD Neonatal Research Network for conducting this study.

WHAT IF I AM INJURED AS A RESULT OF THIS STUDY?

- If either you or your baby is injured or becomes ill from taking part in this study, medical treatment is available at the University of Iowa Hospitals and Clinics.

- No compensation for treatment of research-related illness or injury is available from the University of Iowa unless it is proven to be the direct result of negligence by a University employee.
- If either you or your baby experiences a research-related illness or injury, you and/or your medical or hospital insurance carrier will be responsible for the cost of treatment.

WHAT ABOUT CONFIDENTIALITY?

We will keep your and your baby's participation in this research study confidential to the extent permitted by law. However, it is possible that other people may become aware of your or your baby's participation in this study. For example, federal government regulatory agencies, The National Institute of Health, NICHD Neonatal Research Network, auditing departments of the University of Iowa and the University of Iowa Institutional Review Board (a committee that reviews and approves research studies) may inspect and copy records pertaining to this research. Some of these records could contain information that personally identifies you or your baby.

To help protect your and your baby's confidentiality, we will label information with a code number. The study logs linking the code number with your infant's identity will be kept in a locked office, in a locked file cabinet and password-protected computer files. If we write a report or article about this study or share the study data set with others, we will do so in such a way that you and your baby cannot be directly identified.

A copy of this Informed Consent Document will be placed in your and your baby's medical record.

WILL MY HEALTH INFORMATION BE USED DURING THIS STUDY?

The Federal Health Insurance Portability and Accountability Act (HIPAA) requires University of Iowa Health Care to obtain your permission for the research team to access or create "protected health information" about you for purposes of this research study. Protected health information is information that personally identifies you and your baby and relates to your and your baby's past, present, or future physical or mental health condition or care. We will access or create health information about you and your baby, as described in this document, for purposes of this research and for your baby's treatment. Once University of Iowa Health Care has disclosed your protected health information to us, it may no longer be protected by the Federal HIPAA privacy regulations, but we will continue to protect your and your baby's confidentiality as described under "Confidentiality."

We may share your health information related to this study with other parties including federal government regulatory agencies, the University of Iowa Institutional Review Boards and support staff, and The National Institute of Health, NICHD Neonatal Research Network.
You and your baby cannot participate in this study unless you permit us to use your and your baby's protected health information. If you choose *not* to allow us to use your protected health information, we will discuss any non-research alternatives available to you. Your decision will not affect your right to medical care that is not research-related. Your signature on this Consent Document authorizes University of Iowa Health Care to give us permission to use or create health information about you and your baby.

Although you may not be allowed to see study information until after this study is over, you may be given access to your and your baby's health care records by contacting your health care provider. Your permission for us to access or create protected health information about you and your baby for purposes of this study has no expiration date. You may withdraw your permission for us to use your and your baby's health information for this research study by sending a written notice to: Dr. Edward Bell

University of Iowa Hospitals & Clinics
200 Hawkins Drive
Dept. of Pediatrics, 8811 JPP
Iowa City, Iowa 52242

However, we may still use your and your baby's health information that was collected before withdrawing your permission. Also, if we have sent your and your baby's health information to a third party, such as the study sponsor, or we have removed your identifying information, it may not be possible to prevent its future use. You will receive a copy of this signed document.

IS BEING IN THIS STUDY VOLUNTARY?

Taking part in this research study is completely voluntary. You may choose for you and/or your baby not to take part at all. If you choose for your baby to be in this study, you may stop your and his/her participation at any time. If you decide not to have you and/or your child be in this study, or if you decide to stop you or your baby from participating at any time, you and your baby won't be penalized or lose any benefits for which you otherwise qualify.

Will I Receive New Information About the Study while Participating?

If we obtain any new information during this study that might affect your willingness to continue participating or to let your baby continue participating in the study, we'll promptly provide you with that information.

Can Someone Else End my Participation in this Study?

Under certain circumstances, the researchers or the study sponsor might decide to end your and your baby's participation in this research study earlier than planned. This might happen because your baby needs to be treated in a way outside the study protocol or because funding for the research study is ended.

WHAT IF I HAVE QUESTIONS?

We encourage you to ask questions. If you have any questions about the research study itself, please contact: Dr. Edward Bell at (319)356-4006 or Karen Johnson, R.N. at (319)356-2924.

If you have questions, concerns, or complaints about your rights as a research subject or about research related injury, please contact the Human Subjects Office, 340 College of Medicine Administration Building, The University of Iowa, Iowa City, Iowa, 52242, (319) 335-6564, or e-mail irb@uiowa.edu. General information about being a research subject can be found by clicking "Info for Public" on the Human Subjects Office web site, http://research.uiowa.edu/hso.

OR IRB USE ONLY
APPROVED BY: IRB-01
IRB ID #: 200605740
APPROVAL DATE: 05/19/08
EXPIRATION DATE: 05/19/09

This Informed Consent Document is not a contract. It is a written explanation of what will happen during the study if you decide to participate. You are not waiving any legal rights by signing this Informed Consent Document. Your signature indicates that this research study has been explained to you, that your questions have been answered, and that you agree to take part in this study. You will receive a copy of this form.

Parent Subject's Name (printed): ______________________________________

____ _________________
(Signature of Parent Subject) (Date)

Parent/Guardian or Legally Authorized Representative's Name and Relationship to Subject:

Child Subject's Name (printed): ______________________________________

____ _________________
(Parent/Guardian Name - printed) (Relationship to Subject - printed)

____ __________________
(Signature of Parent/Guardian or Legally Authorized Representative) (Date)

Statement of Person Who Obtained Consent

I have discussed the above points with the subject or, where appropriate, with the subject's legally authorized representative. It is my opinion that the subject understands the risks, benefits, and procedures involved with participation in this research study.

____ _________________________________
(Signature of Person who Obtained Consent) (Date)

APPROVED
AS CORRECTED
Apr 14, 2005
WIRB®
Olympia, WA

RESEARCH SUBJECT INFORMATION AND CONSENT FORM

**TITLE:**	The SUrfactant Positive Airway Pressure and Pulse Oximetry Trial in Extremely Low Birth Weight Infants
**PROTOCOL NO.:**	None<br>WIRB® Protocol #20050156
**SPONSOR:**	National Institute of Child Health and Human Development (NICHD)<br>Bethesda, Maryland<br>United States
**INVESTIGATOR:**	Shahnaz Duara, M.D.<br>Holtz Center (ET5005)<br>1611 NW 12th Avenue<br>Miami, Florida 33136<br>United States
**SITE(S):**	University of Miami<br>Holtz Center (ET5005)<br>1611 NW 12th Avenue<br>Miami, Florida 33136<br>United States
**STUDY-RELATED PHONE NUMBER(S):**	Shahnaz Duara, M.D.<br>305-585-6408<br>305-585-5140 (24 Hours)

PURPOSE

You are being asked to allow your baby to participate in a clinical research study at the University of Miami/Jackson Medical Center, sponsored by the National Institute of Child Health and Human Development (NICHD).

Please read this consent form and ask your study doctor or study staff as many questions as you need to about the study before you decide to be part of the study or not. If there is any word or information that you don't understand, your study doctor or study staff will explain them to you. If after reading this consent form you agree to allow your baby to participate in the study you will be asked to sign this consent form. You may keep an unsigned copy of this consent form to think about or discuss with family or friends before making your decision.

If your baby is born prematurely, he/she will need some assistance breathing and will need to be given supplemental oxygen because his/her lungs may not be mature enough. All babies born at 27 weeks gestational age (3 months before due date) will need some help when they are born. A team of nurses, doctors, and respiratory therapists will evaluate and assist your baby within the first minutes of life. If your baby continues to need support for breathing for more than the first few moments of life, he/she may need to be intubated. This involves having a tube placed through the mouth and wind-pipe (trachea) to better deliver oxygen to the lungs. The mechanical ventilation machine will better deliver extra (supplemental) oxygen to the baby and if needed deliver surfactant (the substance that is lacking in these babies lungs because of their immaturity). All babies born prematurely at extremely low birth weights will need supplemental oxygen. However, too much or too little oxygen may be dangerous to immature organs, causing unwanted illnesses that may have life-long effects during the developmental stages of life.

This study has two aims:

AIM 1:
We want to find out if using Continuous Positive Airway Pressure (CPAP) / Positive End Expiratory Pressure (PEEP) (air pressure given to your baby to keep your baby's lung open) will reduce the need for intubation, the need for surfactant and/or the need for mechanical ventilation (the use of a machine to breathe for your baby) during the first 14 days of life.

AIM 2:
We want to determine what is the best level of oxygen saturation (SpO2) (the amount of oxygen that the blood carries to the organs of the body) required for these very small babies. The current normal levels range from 85% to 95% and this study will allow the comparison of a lower oxygen saturation range (85 to 89%) with a higher 02 saturation range (91% to 95%) until your infant no longer requires oxygen and has matured to within 4 weeks of his/her due date.

Your baby will be one of 1320 babies participating in this study at different hospitals within the United States, and one of 150 babies at the University of Miami/Jackson Children's Hospital.

APPROVED
AS CORRECTED
Apr 14, 2005
WIRB®
Olympia, WA

PROCEDURES

If your baby is born prematurely and you allow your child to participate in this study, immediately after birth the resuscitation will follow usual guidelines. Once your baby is stabilized, your baby will be randomly (by chance, like the flip of a coin) assigned to one of two study groups. Your baby has an equal chance of being in either of the study groups.

TO EVALUATE AIM 1: YOUR BABY WILL BE ASSIGNED A TREATMENT GROUP (CPAP GROUP) OR A CONTROL GROUP (EARLY SURFACTANT GROUP):

A. Treatment Group: (CPAP Group)

These infants will be managed as follow:

Delivery Room Management

If your baby requires positive pressure during resuscitation, CPAP or ventilation with (PEEP) will be used. CPAP will be continued until admission to the Neonatal Intensive Care Unit (NICU). Intubation will be done if your baby requires it.

If your baby requires intubation for resuscitation, he/she will receive surfactant within 60 minutes of birth. The other aspects of the resuscitation will be managed according to the Neonatal Resuscitation Program (NRP) guidelines and follow current practice.

NICU Management

If your baby is in the treatment group, while in the NICU, your baby will be managed on nasal CPAP. If your baby needs intubation, specific requirements for intubation (tube insertion), extubation (tube removal) and reintubation (reinsertion of the tube) have been developed and will continue in effect for a minimum of 14 days of life.

If your baby is intubated in the first 48 hours for respiratory distress, he/she will be given a minimum of one dose of surfactant. Up to 4 doses of surfactant may be given if needed.

B. Control Group: (Early surfactant and ventilation)

If your baby is in the control group, he/she will be treated using an approach considered similar to current standards of care. It is anticipated that a majority of these infants will be intubated and receive surfactant in the delivery room.

APPROVED
AS CORRECTED
Apr 14, 2005
WIRB®
Olympia, WA

Infants in this group will be managed as follows:

Delivery Room Management

Infants will be intubated in the delivery room and given surfactant or receive surfactant within 60 minutes of birth. The other aspects of the resuscitation will be managed according to the NRP guidelines and follow current practice for the NICU.

NICU Management

Infants will continue to receive mechanical ventilation until it is no longer needed. These requirements will continue in effect for a minimum of 14 days for all infants.

TO EVALUATE AIM 2: (LOW VERSUS HIGH SPO2 RANGE)

In addition, once your baby is in the NICU, he/she will be randomly assigned to have the high level of oxygenation (91 to 95%) or the low level of oxygenation (85 to 89%). Your baby has an equal chance of being assigned to either study group. Both groups are within the range of usual standard of care for the NICU. Your baby will remain in the group he/she is assigned to as long as he/she requires oxygen.

The study pulse oximeter (a device used to measure your baby's oxygen level) will be applied to your baby within two hours after going to the NICU. The assigned pulse oximeter will remain on your baby and will be removed once your baby has been in room air and off ventilatory support or CPAP for 72 hours. If oxygen is required later on, the same oxygen levels will be used until 36 weeks gestational age.

The assigned oximeter will be used as long as the baby is receiving ventilator support. The oximeters will alarm if they are too low (84%) or too high (96%).

Once your baby is admitted to the NICU, he/she will receive the standard care according to the policies and procedures set by the NICU.

An ultrasound of your baby's head will be done between days 4 and 21, if one has not already been done. All babies will be seen at about 2 years of age for developmental assessment.

RISKS

The major risk of this study is that your baby may not receive surfactant as early. Early surfactant is known to reduce disease severity and the chance of death. It also reduces lung damage.

Potential risks to the use of CPAP and/or PEEP include collapsed lung, decreased heart rate, blood pressure problems, and distention of the stomach. The risks of ventilation include damage to the airways or lungs, damage from the tube moving or becoming plugged. There may also be heart rate and blood pressure problems.

Prolonged exposure to very high levels of oxygen may cause complications to your baby such as damage to the eyes which can result in blindness. Complications of too little oxygen may include problems with childhood development.

There may be risks or side effects which are unknown at this time.

Your baby's condition may not get better or may become worse while he/she is in this study.

NEW FINDINGS

You will be told about any new information that might change your decision to allow your child to be in this study.

BENEFITS

The consequences of your baby's breathing problems may be improved because of participating in this study, however, this cannot be guaranteed. It is possible that there may be no direct medical benefit to your baby for participating. The information learned in this study may benefit other babies having similar problems in the future.

COST TO YOU

There is no cost to you for participating in this study.

ALTERNATIVES

You have the alternative not to participate in this study and have your baby receive standard care after delivery. If you choose not to allow your baby to participate in the study, he or she will receive the standard care used at this institution. Ask the study doctor to discuss this with you.

CONFIDENTIALITY

Information from this study will be given to the sponsor. "Sponsor" includes any persons or companies which are contracted by the sponsor to have access to the research information during and after the study.

The information will also be given to the U.S. Food and Drug Administration (FDA). It may be given to governmental agencies in other countries where the study drug may be considered for approval. Medical records which identify your baby and the consent form signed by you will be looked at and/or copied for research or regulatory purposes by:

- the sponsor;
- Research Triangle Institute, an agent for the sponsor;

and may be looked at and/or copied for research or regulatory purposes by:

- the FDA;
- Department of Health and Human Services (DHHS) agencies;
- governmental agencies in other countries;
- the University of Miami; and
- the Western Institutional Review Board® (WIRB®).

Absolute confidentiality cannot be guaranteed because of the need to give information to these parties. The results of this research study may be presented at meetings or in publications. Your baby's identity will not be disclosed in those presentations.

Finally, if you should seek treatment at Jackson Health System, information from this study may be given to the treating physicians and other medical staff at Jackson Health System. In turn, the treating physicians and other medical staff at Jackson Health Systems may provide information about your baby's treatment and care to the study doctor and/or agents for the study doctor.

COMPENSATION FOR INJURY

Your baby may be exposed to risk of injury from participation in this study. If injury occurs, treatment will in most cases be available. If you have insurance, your insurance company may or may not pay these costs. If you do not have insurance, or if your insurance company refuses to pay, you will be billed for these costs. Funds to compensate for pain, expenses, and other damages caused by injury are not routinely available.

SOURCE OF FUNDING

Funding for this research study will be provided by National Institute of Child Health and Human Development (NICHD).

VOLUNTARY PARTICIPATION/WITHDRAW

Your agreement to have your infant participate in this study is voluntary. You have the right to withdraw your consent or take your baby out of the study at any time. If you do decide to take your baby out of the study, he/she will continue to receive the same level of care that the hospital and the doctors provide. If you decide not to allow your baby to participate, or if you decide to stop participating at any time, there will be no penalty, nor loss of benefits, nor loss of medical care to which you or your baby are otherwise entitled.

The study doctor, Dr. Shahnaz Duara, or the sponsor of this study may decide to remove your baby from the study at any time without your consent if it is felt to be in the best interest of your baby.

QUESTIONS

You may ask questions, and request information about this research study at any time during the study. Dr. Shahnaz Duara or her study assistants will be available to answer any questions you may have at 305-585-6408 between 8:00am to 5:00pm or the physician on call in the NICU at 305-585-5140 (24 Hours).

If you have any questions about your rights, or the rights of your infants, as a research subject you may contact:

Western Institutional Review Board® (WIRB®)
3535 Seventh Avenue, SW
Olympia, Washington 98502
Telephone: 1-800-562-4789.

WIRB is a group of people who perform independent review of research.

Do not sign this consent form unless you have had a chance to ask questions and have received satisfactory answers to all of your questions.

If you agree to allow your baby to participate in this study, you will receive a signed and dated copy of this consent form for your records.

CONSENT

I have read the information in this consent form (or it has been read to me). I have discussed this study and the information provided with the study doctor. All my questions about the study and my baby's participation in it have been answered. I freely consent to allow my baby to participate in this research study.

I authorize the release of my baby's medical records for research or regulatory purposes to the sponsor, Research Triangle Institute, the FDA, DHHS agencies, governmental agencies in other countries, the University of Miami, and WIRB®.

APPROVED
AS CORRECTED
Apr 14, 2005
WIRB®
Olympia, WA

By signing this consent form, I have not waived any of the legal rights which I or my child otherwise would have as a subject in a research study.

____
Printed Name of Subject

Signature of Mother	Date
Signature of Father (if available)	Date

OR

Signature of Legally Authorized Representative	Date

Authority of Subject's Legally Authorized Representative or Relationship to Subject

Signature of Witness	Date
Signature of Person Conducting Informed Consent Discussion	Date

Print name

SUBJECT IMPRINT

JACKSON MEMORIAL HOSPITAL
Miami, FLORIDA

JMHP 01-0305-1
3-1-79
C-640 CLINICAL RESEARCH CONSENT FORM

APPROVED
AS CORRECTED
Apr 14, 2005
WIRB®
Olympia, WA

---------------------------------- **Use the following only if applicable** --------------------------------

If this consent form is read to the subject because the legally authorized representative is unable to read the form, an impartial witness not affiliated with the research or investigator must be present for the consent and sign the following statement:

I confirm that the information in the consent form and any other written information was accurately explained to, and apparently understood by, the subject's legally authorized representative. The subject's legally authorized representative freely consented to participate in the research study.

______________________________________ ____________________

Signature of Impartial Witness Date

Note: This signature block cannot be used for translations into another language. A translated consent form is necessary for enrolling subjects who do not speak English.

Study Doctor: Shahnaz Duara, M.D.
Telephone: 305-585-6408 (office)
305-585-5140 (nights and weekends)

SUBJECT IMPRINT

JACKSON MEMORIAL HOSPITAL
Miami, FLORIDA

JMHP 01-0305-1
3-1-79
C-640 CLINICAL RESEARCH CONSENT FORM

The University of New Mexico Health Sciences Center
Consent to Participate in Research

The SUrfactant Positive Airway Pressure and Pulse Oximetry Trial in Extremely Low Birth Weight Infants (SUPPORT Trial)

Your child is invited to participate in a research study being done by Kristi Watterberg. M.D., who is the Principal Investigator, and her associates, from the Department of Pediatrics. This research is looking at two basic questions: which of two lung treatments used with premature babies is better for the baby's lungs; and what is the appropriate level of oxygen in the blood in premature infants. The two lung treatments are "CPAP" (positive air pressure to help keep the lungs inflated) and "intubation," which involves placement of a breathing tube in the infant's airway with early administration of a medication called surfactant through this breathing tube.

Since 1970 two basic ways to help premature babies breathe have been used in Newborn Intensive Care Units. One method, called "intubation," involves placement of a breathing tube in the infant's airway and attaching it to a machine called a "ventilator" which breathes for the infant. The other method, called "CPAP" or "Continuous Positive Airway Pressure," involves placement of short tubes in the infant's nose and providing air pressure which helps the infant breathe on his/her own. Many studies have been done to see how to make these two methods work as well as possible. Research has shown, regardless of which treatment is used, that all babies who need help with breathing are at risk of developing a type of chronic, or long lasting, lung disease called "Bronchopulmonary Dysplasia," or "BPD" for short. Since 1990 a medication, called "surfactant" has been available to help premature babies breathe easier, but a tube must be placed in the airway to give this medicine.

Oxygen is also used whenever a baby is not able to get enough oxygen into his/her blood by breathing room air. Doctors know that it is important to be sure a baby is getting enough, but not too much oxygen. One possible complication of too much oxygen is an eye disease called "Retinopathy of Prematurity," or "ROP," that may result in poor vision or even blindness.

All of these treatments have been carefully studied and all are used in Newborn ICUs. This is the first study to carefully compare the use of all of these methods starting from the first moments after birth and following the babies until at least 18 months after they would have been born, if they had not been premature.

In this study, infants who receive delivery room CPAP and who have specific guidelines for having a breathing tube placed will be compared to infants who have a breathing tube placed and surfactant given in the delivery room or very soon after birth. The study will also compare keeping a lower range (85-89%) or a higher range (91-95%) of oxygen levels in the blood (saturation). Both of these ranges are within the oxygen saturation range that is currently used for premature infants in the NICU at UNM Hospital (85 – 95%). An alarm will sound if the oxygen saturation goes above or below that range, the same as for other premature infants in our NICU. While it is known that higher oxygen

OFFICIAL USE ONLY
APPROVED 08/05/08 EXPIRES 08/14/09
The University of New Mexico Human Research Review Committee

ranges are associated with more eye disease, the safest oxygen range is still unknown. We hope to find out if a lower range results in less ROP (Retinopathy of Prematurity).

You are being asked to allow your child to be in the study because there is a possibility that (s)he will be born 12-16 weeks early (at 24 to 28 weeks of pregnancy). About 30 babies will take part in this study at the University of New Mexico. 1300 babies will participate in this study across the United States. The National Institutes of Health is funding this study.

This form will explain the research study, and will also explain the possible risks as well as the possible benefits to you. We encourage you to talk with your family and friends before you decide to take part in this research study. If you have any questions right now, please ask one of the study investigators.

What will happen if I decide to participate?

If you decide to allow your child to be in this study, a few minutes before your child is born, (s)he will be randomly assigned, like the flip of a coin, to one of two lung treatments. The treatments are as follows: 1) CPAP in the delivery room immediately after birth and continuing in the intensive care nursery (NICU), or 2) placement of a tube in the windpipe in the delivery room or right after arrival in the NICU, followed by surfactant administration and ventilation (breathing for the baby using a machine). It is not known which of these breathing treatments is better. Infants randomized to the CPAP group may, at some point in their care, require a windpipe tube and a breathing machine. Your baby's doctors will make that decision if they think it is necessary for your baby. Studies have suggested that babies who have a breathing tube placed and surfactant given very early may benefit from the surfactant (Some studies have shown that very early surfactant results in less "air leak", where air escapes from the air spaces of the lungs into the area around the lungs, and less death, but other studies have not found this difference), but they may have a higher risk for developing BPD because of the breathing tube. On the other hand, infants treated with early CPAP may not receive the early benefit of surfactant, but may have a lower risk for developing BPD because no breathing tube is inserted.

In addition to being randomly assigned to one of the two groups described above, your baby will be randomly assigned to having an oximeter (a machine that monitors oxygen in the blood) which reads slightly high or one that reads slightly low. The oximeters used in this study are FDA approved devices which have been adjusted for research purposes to show an oxygen saturation value which is either a little higher or a little lower than the true oxygen reading when the oxygen is in the normal range (between 85 and 95%). Outside the normal range, the oximeter shows the true oxygen level. This will help to protect your baby from oxygen levels that are too high or too low.

Your infant will be assigned to one of the four groups shown below. Neither you nor your baby's doctors will know which oxygen saturation range is being targeted; however, the alarm limits will remain the same as for all premature infants in our NICU. Your baby will not be exposed to lower or higher oxygen saturations than are currently accepted for all premature infants in our NICU. The assignments will be made randomly, like the flip of a coin.

OFFICIAL USE ONLY
APPROVED 08/05/08 EXPIRES 08/14/09
The University of New Mexico Human Research Review Committee

CPAP<br><br>Higher oxygen level in the blood	CPAP<br><br>Lower oxygen level in the blood
Breathing tube + breathing machine + surfactant<br><br>Higher oxygen level in the blood	Breathing tube + breathing machine + surfactant<br><br>Lower oxygen level in the blood

All the rest of your infant's care will be the standard treatments for premature babies in the UNM Children's Hospital NICU. Information about that care and certain results, such as head ultrasounds and growth measurements, will be collected from your child's medical record. At 6 months and at 12 months we will call you and ask you questions about your child's breathing (especially wheezing and coughing), medication use, and visits to a Doctor, Emergency Room, or Hospital visits for treatment of breathing problems. We will also ask questions about family history of breathing problems, and questions about your home, including things that may increase your child's risk of breathing problems. It will take about 15 minutes to answer these questions. The children enrolled in this study will need to come to Special Baby Clinic for their 18-22 month old assessments of growth, breathing problems, development, and coordinated movement skills.

How long will my child be in this study?

Study guidelines for lung treatments of infants in both groups will be followed for two weeks. Your child will be on a study oximeter until about four weeks before the original "due date". At that time, the oximeter will be changed to a standard one for the remainder of his/her hospital stay. The early part of the project will last until the end of your child's hospital stay. In order to evaluate the long term effects of the treatments in this study, information will be collected about your baby's general health, and any hospitalizations during the first two years of life. By agreeing to participate in this study, you give consent for the release of medical records from other medical facilities and providers of medical care to Dr. Kristi Watterberg and her associates. Follow up at 18-22 months is essential for this study. Families who participate in this project are agreeing to remain in contact with the investigators and to return to the UNM Children's Hospital's Special Baby Clinic with their child when (s)he is 18 months of age.

What are the risks or side effects of being in this study?

Participation in this study may involve some added risks or discomforts. Some unknown risks may be learned during this study. All of these treatments are currently clinically accepted, but haven't been

OFFICIAL USE ONLY
APPROVED 08/05/08 EXPIRES 08/14/09
The University of New Mexico Human Research Review Committee

compared with each other in this manner, so we are not able to predict which group may do better. One or both of these two treatments is the usual clinical practice for all babies who are this premature at UNMH. If your baby is assigned to the group of babies who have a breathing tube placed and surfactant given very early, he/she may benefit from the surfactant, but may have a higher risk for developing BPD because of the breathing tube. If your baby is assigned to the CPAP group, he/she may not receive the early benefit of surfactant, but may have a lower risk for developing BPD because no breathing tube is inserted. The CPAP mask can cause a low heart rate. If this happens, the mask is removed and repositioned. CPAP can cause air to collect in the stomach, so a small tube is placed from the mouth into the stomach. All babies are monitored for these problems.

For this study, there will be no change in the oxygen saturation range from the one that is currently used in the NICU at UNMH. In this study we are trying to further narrow the range that we are currently using. The specific and ideal oxygen range to reduce eye disease is unknown. We hope that this study will help to determine if a lower or higher oxygen range may be better. The higher and lower ranges that are used in this study are both in the oxygen range that is currently used for all babies admitted to the UNM NICU. The only other risk of this study is the risk to confidentiality. Every effort will be made to keep your child’s medical record confidential.

What are the benefits to being in this study?

Your child may or may not be benefit from participating in this study. The knowledge learned from this study may help us treat babies in the future.

What other choices do I have if I do not want to be in this study?

The alternative to having your child participate in this project is not to participate. You should not feel obligated to agree to participate. Your questions should be answered clearly and to your satisfaction. If you choose not to have your child participate he/she will receive the standard care for premature infants as needed. This may include oxygen and help to breathe that is similar to the treatments in this study.

How will my information be kept confidential?

We will take measures to protect your privacy and the security of all your personal information, but we cannot guarantee confidentiality of all study data.

Information contained in your study records is used by study staff and, in some cases it will be shared with the sponsor of the study. The University of New Mexico Health Sciences Center Human Research Review Committee (HRRC) that oversees human subject research, the National Institutes of Health which sponsors this study, and the Food and Drug Administration and/or other regulatory entities will be permitted to access your records. There may be times when we are required by law to share your information. However, your name will not be used in any published reports about this study. A copy of this consent form will be kept in your medical record.

What are the costs of taking part in this study?

There are no costs involved in taking part in this study. You or your insurance company will be responsible for the costs incurred in your child's care because that care will not be different from what

OFFICIAL USE ONLY
APPROVED 08/05/08 EXPIRES 08/14/09
The University of New Mexico Human Research Review Committee

is usually provided by the nursery staff. The National Institutes of Health and National Institute of Child Health and Human Development are providing financial support and/or materials for this study.

What will happen if I am injured or become sick because I took part in this study?

No commitment is made by the University of New Mexico Health Sciences Center (UNMHSC) to provide free medical care or money for injuries to participants in this study. If your child is injured or become*s* sick as a result of this study, UNMHSC will provide him or her with emergency treatment, at your cost. It is important for you to tell your study doctor immediately if your child has been injured or become*s* sick because of taking part in this study. If you have any questions about these issues, or believe that you have been treated carelessly in the study, please contact the Human Research Review Committee (HRRC) at the University of New Mexico Health Sciences Center, Albuquerque, New Mexico 87131, (505) 272-1129 for more information.

Will I be paid for taking part in this study?

No payment will be provided for this project.

How will I know if you learn something new that may change my mind about participating?

You will be informed of any significant new findings that become available during the course of the study, such as changes in the risks or benefits resulting from participating in the research or new alternatives to participation that might change your mind about participating.

Can I stop being in the study once I begin?

Your participation in this study is completely voluntary. You have the right to choose not to participate or to withdraw your participation at any point in this study without affecting your future health care or other services to which you are entitled.

At the discretion of the clinical provider, babies may be taken out of this study due to unanticipated circumstances. Examples of reasons for taking a participant out of the study include: the investigator deciding that continued participation could be harmful to your child or the study being canceled

Who can I call with questions or complaints about this study?

If you have any questions, concerns or complaints at any time about the research study, Kristi Watterberg, M.D., or her associates will be glad to answer them at (505) 272-0180 on Monday through Friday between 9 am and 5 pm. If you would like to speak with someone other than the research team, you may call the UNMHSC HRRC at (505) 272-1129.

Who can I call with questions about my rights as a research subject?

If you have questions regarding your rights as a research subject, you may call the UNMHSC HRRC at (505) 272-1129. The HRRC is a group of people from UNM and the community who provide independent oversight of safety and ethical issues related to research involving human subjects. For more information, you may also access the HRRC website at http://hsc.unm.edu/som/research/hrrc/.

Consent

You are making a decision whether to have your child participate in this study. Your signature below indicates that you read the information provided (or the information was read to you). By signing this consent form, you are not waiving any of your child's legal rights as a research subject.

I have had an opportunity to ask questions and all questions have been answered to my satisfaction. By signing this consent form, I agree to participate or let my child participate in this study. A copy of this consent form will be provided to you.

____
Name of Parent/Child's Legal Guardian

___________________________________ _______________
Signature of Parent/Child's Legal Guardian Date

I have explained the research to the subject and his/ her parent/legal representative, and answered all of his/ her questions. I believe that he/she understands the information described in this consent form and freely consents to participate.

_____________________________ _________________________/ __________
Name of Investigator/ Signature of Investigator/ Date
Research Team Member Research Team Member

OFFICIAL USE ONLY
APPROVED 08/05/08 EXPIRES 08/14/09
The University of New Mexico Human Research Review Committee

Is your child participating in any other research studies? ______ yes ______ no

Informed Consent

Your child is invited to participate in a research study to find out more about treatment with CPAP (positive air pressure to help keep the lungs inflated) and learn the appropriate levels of oxygen in the blood in premature infants. You are being asked to allow your child to be in the study because there is a possibility that (s)he will be born 12-16 weeks early (at 24 to 28 weeks of pregnancy).

The study, funded by the National Institutes of Health, is being conducted at Stanford and other medical centers across the country. The study will compare two ways of assisting premature infants to breathe. Infants who receive delivery room CPAP and who have specific guidelines for having a breathing tube placed will be compared to infants who have a breathing tube placed and surfactant (a liquid which helps babies with immature lungs breath easier by helping keep their lungs from collapsing) given in the delivery room. The study will also compare management of infants in lower range (85-89%) and higher range (91-95%) oxygen levels (saturation). We hope to determine if a lower range results in decreased ROP (Retinopathy of Prematurity, an eye disease that may result in impairment of vision or even blindness, which may be caused by excessive levels of oxygen.) Nationwide, a total of 1300 patients are expected to enroll in this study over about two years. We expect about 60 of those infants will be from Stanford. Children who are enrolled in the study will be involved for about two years.

If you decide to allow your child to be in this study, a few minutes before your child is born, (s)he will be randomly assigned, like the flip of a coin, to one of two lung treatment strategies. The treatments are as follows: 1) CPAP in the delivery room immediately after birth and continuing in the intensive care nursery (NICU), or 2) placement of a tube in the windpipe in the delivery room followed by surfactant administration and ventilation (breathing for the baby using a machine). Infants randomized to the CPAP group may, at some point in their care, require a windpipe tube and a breathing machine. If the attending physician deems this necessary, participation in the study will not affect this decision. Study guidelines for lung treatments of infants in both groups will be followed for two weeks.

In addition to being randomly assigned to one of the two groups described above, your baby will be randomly assigned to having an oximeter (blood oxygen monitor) which reads slightly high or one that reads slightly low. The oximeters used in this study are FDA approved devices which have been modified for research purposes. This modification makes the monitors show a oxygen

Participant ID

STUDY

saturation value which is either a little higher or a little lower than the true oxygen reading when values are in the normal range (between 85 and 95%). Outside those ranges, the oximeter works the same as a standard oximeter. This will allow us to keep the saturations at the high and low ends of the normal range and still protect the study infants from undesirable oxygen levels. The doctors and nurses taking care of your infant will not know if (s)he is in the high or low saturation group. This is to help assure that all patients are cared for in the same way. Your child will be on a study oximeter until about four weeks before the original "due date". At that time, the oximeter will be changed to a standard one for the remainder of his/her hospital stay.

Your infant will be assigned to one of the four groups shown below. Neither you or the doctors taking care of your infant will be able to choose which group your infant is assigned to. The assignments will be made randomly, like the flip of a coin.

CPAP<br><br>Higher oxygen saturation	CPAP<br><br>Lower oxygen saturation
Breathing tube + breathing machine<br><br>Higher oxygen saturation	Breathing tube + breathing machine<br><br>Lower oxygen saturation

Part of your child's regular care during the first few weeks after birth will include one or more head ultrasounds and, within about four weeks of your child's planned due-date, an MRI of the brain. The ultrasounds and MRI studies create pictures (images) of the brain which are used to look for brain injury. Children who participate in this study will have an ultrasound at the time the MRI is done so the doctors conducting the project can compare the findings and determine if one way of imaging gives more useful information than the other.
Other aspects of your infant's care will be the standard treatments for premature babies in the Stanford NICU. All children who participate in the project will return to the Development and

Participant ID

STUDY

Behavior Unit at regular intervals during the first two years as part of their routine care. When the children enrolled in this study return for their 18-22 month old assessments of growth, development, and coordinated movement skills, the study will collect that outcome information.

Additional Breathing Follow-Up

Many children who were born prematurely and needed help to breathe continue to have breathing problems such as wheezing and coughing in the first two years of life. The study would like to stay in touch with you by telephone beginning when your baby goes home and continuing every six months over the next 18-22 months, a total of four times. At these times, the caller will ask questions about your child's breathing, medication use, and visits to a doctor, emergency room, or hospital for treatment of breathing problems. The caller will also ask several questions about you and your family. Each call should take about 15 minutes of your time, less if your baby has had no breathing problems. The results from your baby's questionnaires will be combined with other babies from around the country. However, your baby's name will not be used.

If you decide you would like to participate in the telephone questionnaires, we will ask for your telephone contact information around the time that your baby is getting ready to go home. Your name and contact information will be given to the study calling center, based at the University of Rochester in Rochester, NY. Although they will have your name, the information you give them will be identified by your baby's study number, not your or your baby's name. Additionally, the study calling center will not disclose your name or contact information to any other person or entity.

Please indicate your decision below:

______ Yes, I agree to participate in the telephone questionnaires

______ No, I do not want to participate in the telephone questionnaires

Participation in this study may involve some added risks or discomforts. Because all of the treatments proposed in this study are within standard of care, there is no predictable increase in risk for your baby. There is no known risk or discomfort associated with the extra head ultrasound. Some unknown risks may be learned during the study. You will be told of any new

Participant ID

STUDY

information that is learned which may affect your child's condition or influence your willingness to have him/her continue participation in this study. The only other risk of this study is the risk to confidentiality. Every effort will be made to keep your child's medical record confidential.

There may be benefits to your child directly, including a possible decrease in chronic lung disease (need for extra oxygen near discharge) and/or a decrease in the need for eye surgery as a result of exposure to oxygen. Because we do not know in advance the actual strategies chosen for your child, or which of the treatment strategies is the most effective, it is also possible that your baby will receive no direct benefit. The knowledge learned from this study may help us treat babies in the future. However, each of the 4 possible combinations of treatments is considered by some NICUs to represent their desired approach.

WE CANNOT AND DO NOT GUARANTEE OR PROMISE THAT YOUR CHILD WILL RECEIVE ANY BENEFIT FROM THIS STUDY.

The alternative to having your child participate in this project is not to participate. You should not feel obligated to agree to participate. Your questions should be answered clearly and to your satisfaction. If you choose not to have your child participate, he/she will receive the standard care for premature infants including oxygen, and help to breathe as needed.

Any data that may be published in scientific journals or presented at scientific or medical meetings will not reveal the identity of your child. Patient information may be provided to Federal and regulatory agencies as required. The Food and Drug Administration, for example, may inspect research records and learn your identity if this study falls within its jurisdiction.

Your child's participation in this study is entirely voluntary.
Your decision whether or not to allow your child to participate will not prejudice your child or his/her medical care. If you wish to allow your child to participate in this study, you must sign this consent form and the authorization form. If you decide to let your child participate, you are free to withdraw your consent, including your authorization regarding the use and disclosure of your child's health information, and to discontinue participation at any time without prejudice to your child or effect on your child's medical care. If you decide to terminate your child's participation in this study, you should notify Dr Van Meurs or Dr Stevenson at (650) 723 5711.
The early part of the project will last until the end of your child's hospital stay. In order to successfully evaluate the approaches to lung treatment and oxygen used in this study, Dr. Van Meurs and her associates will want to collect information about your baby's general health, and any hospitalizations during the first two years of life. By agreeing to participate in this study, you

Participant ID

STUDY

give consent for the release of medical records from other medical facilities and providers of medical care to Dr. Krisa Van Meurs and her associates. Follow up at 18-22 months is essential for this study. Families who participate in this project are agreeing to remain in contact with the investigators and to return to the Mary L. Johnson Development and Behavior Unit at Packard Children's Hospital with their child when (s)he is 18 months of age.

While participating in this study, your child should not take part in any other research project without approval from all of the investigators. This is to protect your child from possible injury arising from such things as extra blood drawing, extra x-rays, interaction of research drugs, or similar hazards.

You and your child will not be paid to participate in this research study. You or your insurance company will be responsible for the costs incurred in your child's care because that care will not be different from what is usually provided by the nursery staff. The study will pay for the extra ultrasound obtained around the due-date. The National Institutes of Health and National Institute of Child Health and Human Development are providing financial support and/or materials for this study.

At the discretion of the protocol director subjects may be taken out of this study due to unanticipated circumstances. Examples of reasons for taking a participant out of the study include: *failure to follow the instructions of the Protocol Director and study staff, the investigator deciding that continued participation could be harmful to your child, the study being canceled, some other administrative reason.

If you have any questions, concerns or complaints about this research study, its procedures, risks and benefits, or alternative courses of treatment, you should ask the protocol director, Dr Van Meurs at 650 723 5711. You should also contact her at any time if you feel your child has been hurt by being a part of this study. If you cannot reach the protocol director, please page the research team at 415 607 4326.

If you are not satisfied with how this study is being conducted, or if you have any concerns, complaints, or general questions about the research or your child’s rights as a participant, please contact the Stanford Institutional Review Board (IRB) to speak to someone independent of the research team at 650 723 5244 or toll free at 1 866 680 2906. You can also write to the Stanford IRB, Stanford University, Stanford, CA 94305-5401.

Participant ID

STUDY

All forms of medical diagnosis and treatment, whether routine or experimental, involve some risk of injury. In spite of all precautions, your child might develop medical complications from participating in this study. If such complications arise, the protocol director will assist you in obtaining appropriate medical treatment. In the event that your child has an injury or illness that is directly caused by participation in this study, reimbursement for all related costs of care first will be sought from your insurer, managed care plan, or other benefits program. You will be responsible for any associated co-payments or deductibles as required by your insurance. If costs of care related to such an injury are not covered by your insurer, managed care plan or other benefits program, you may be responsible for these costs. If you are unable to pay for such costs, the protocol director will assist you in applying for supplemental benefits and explain how to apply for patient financial assistance from the hospital. Additionally, Stanford is not responsible for research and medical care by other institutions or personnel participating in this study. You do not waive any liability rights for personal injury by signing this form.

As a research participant your child has the following rights. These rights include but are not limited to the participant's right to:

- be informed of the nature and purpose of the experiment;
- be given an explanation of the procedures to be followed in the medical experiment, and any drug or device to be utilized;
- be given a description of any attendant discomforts and risks reasonably to be expected;
- be given an explanation of any benefits to the subject reasonably to be expected, if
- applicable;
- be given a disclosure of any appropriate alternatives, drugs, or devices that might be advantageous to the subject, their relative risks and benefits;
- be informed of the avenues of medical treatment, if any, available to the subject after the experiment if complications should arise;
- be given an opportunity to ask any questions concerning the experiment or the procedures involved;
- be instructed that consent to participate in the medical experiment may be withdrawn at any time and the subject may discontinue participation without prejudice;
- be given a copy of the signed and dated consent form, and be given the opportunity to decide to consent or not to consent to a medical experiment without the intervention of any element of force, fraud, deceit, coercion or undue influence on the subject's decision.

Participant ID

STUDY

YOUR SIGNATURE INDICATES THAT YOU HAVE READ AND UNDERSTAND THE ABOVE INFORMATION, THAT YOU HAVE DISCUSSED THIS STUDY WITH THE PERSON OBTAINING CONSENT, THAT YOU HAVE DECIDED TO PARTICIPATE BASED ON THE INFORMATION PROVIDED, AND THAT A COPY OF THIS FORM HAS BEEN GIVEN TO YOU.

Signature of Parent	Date	Authority to act for participant
(If available) Signature of Other Parent	Date	Authority to act for participant

The IRB determined that the permission of one parent is sufficient for research to be conducted under 45 CFR 46.404, in accordance with 45 CFR 46.408(b).

Person Obtaining Consent

I attest that the requirements for informed consent for the medical research project described in this form have been satisfied-that the participant has been provided with the Experimental Subject's Bill of Rights, if appropriate, that I have discussed the research project with the participant and explained to him or her in nontechnical terms all of the information contained in this informed consent form, including any risks and adverse reactions that may reasonably be expected to occur. I further certify that I encouraged the participant to ask questions and that all questions asked were answered.

Signature of Person Obtaining Consent	Date

Participant ID

STUDY

UNIVERSITY OF
ROCHESTER
MEDICAL CENTER

EASTMAN DENTAL CENTER
SCHOOL OF MEDICINE AND DENTISTRY
SCHOOL OF NURSING
STRONG MEMORIAL HOSPITAL
UNIVERSITY MEDICAL FACULTY GROUP

DEPARTMENT OF PEDIATRICS
GOLISANO CHILDREN'S HOSPITAL AT STRONG
DIVISION OF NEONATOLOGY

UNIVERSITY OF ROCHESTER RSRB
MAR 28 2007
EXPIRES

Permission Form

Study Title: The Surfactant Positive Airway Pressure and Pulse Oximetry Trial in Extremely Low Birth Weight Infants.

Principal Investigator: Nirupama Laroia, MD

Introduction

This permission form describes a research study and what you may expect if you decide to have your child participate. You are encouraged to read this form carefully and to ask the person who presents it any further questions you may have before making your decision whether or not to have your child participate.

Your child is being asked to participate in this study because your child will likely be born at 24 weeks to 27 weeks and 6 days gestational age (time from your last menstrual period) and your child may have breathing problems due to premature birth.

This form describes the known possible risks and benefits and describes what other choices for care or services are available to your child if you do not wish to have your child be in the study. You are completely free to choose whether or not to have your child participate.

Background

Many premature babies have difficulty expanding their lungs at birth and establishing breathing. The usual treatment is to give them oxygen and inflate their lungs with a ventilation bag. If breathing is not effective, then a small tube is passed into the windpipe to assist the baby's breathing using a ventilator (breathing machine or respirator). This is effective and is used for the majority of very premature babies who need assistance with their breathing. However, use of a breathing tube and mechanical ventilation can result in problems (complications) to the baby.

There is new evidence that even very premature babies might be helped to breathe on their own, without the need for a ventilator, if the lungs are gently and continuously inflated beginning soon after birth. This technique is called Nasal Continuous Positive Airway Pressure or Nasal CPAP; it means that oxygen is provided at constant pressure to the airway through a tube in the nostrils. Nasal CPAP is a treatment that is regularly used in neonatal intensive care to assist the breathing of babies with minor respiratory difficulties. It is most commonly used to help babies after they have been weaned from (taken off) the ventilator. Nasal CPAP is not usually used to help babies breathe immediately following birth, but it is used in a few hospitals. Available information suggests that this method may be successful in helping some infants born very prematurely.

601 Elmwood Avenue, Box 651
Rochester, New York 14642
(585) 275-2972
Fax: (585) 461-3614

There is currently no solid information that enables us to decide which of these techniques is best for assisting a baby's initial breathing.

In addition, premature babies sometimes develop an eye disease known as Retinopathy of Prematurity (ROP) which can result in loss of vision or even blindness. The level of oxygen in the baby's blood may have an effect on whether the baby develops ROP. All babies that are born prematurely have the amount of oxygen in their blood monitored almost continuously for several weeks using a pulse oximeter. The pulse oximeter measures the amount of oxygen by shining a light through the hand or foot. The light sensor is attached with tape like a bandage. The levels of oxygen in the blood usually range from 85% to 95%. We do not know, however, if it is better to keep the baby's oxygen level in the lower range (85% - 89%) or the higher range (91% - 95%) and what effect this may have on developing ROP or long term development.

Purpose of the Study

The purpose of this research study is to find out whether starting nasal CPAP (providing oxygen through a nasal tube) at birth is better, just the same as, or worse than using a ventilator and breathing tube for babies born very prematurely. The study will also compare low range (85%-89%) oxygen saturation levels with high range (91% - 95%) levels to determine if a lower oxygen saturation range results in decreased eye disease (ROP), and is safe.

Duration of Participation

Study participation will begin as soon as the infant is born. The baby will remain in the study for the duration of hospitalization. Your baby will be followed after discharge through telephone interviews with you when your baby is six and twelve months of age, and either by telephone or in person when your baby returns for a follow-up study visit at 18-22 months after the date your baby would have been born if full term.

Description of Study Procedures

If you agree to have your baby participate in this study, your baby will be randomly assigned (like flipping a coin) prior to delivery to receive, either:

(1) Continuous positive airway pressure (CPAP) in the delivery room immediately after birth and continuing in the NICU or (2) Assisted ventilation using a tube in the windpipe (endotracheal tube) followed by surfactant administration. Surfactant is a liquid that helps babies with immature lungs breath easier by helping to keep their lungs from collapsing. Surfactant may also be given to infants in the first group if they show they need it in the NICU.

In addition to being randomly assigned to one of these two groups, your baby will also be randomly assigned to 1 of 2 levels of blood oxygen. This will be done using special oximeters (oxygen monitors). Within the range of oxygen that we normally keep babies in (85 to 95%), your baby will either be in the high end of normal or the low end of normal. The nurse taking care of your baby or his/her physician will not know to which blood oxygen group your baby has been assigned. Your child will remain on this device until he/she reaches 36 weeks ad[illegible]

UNIVERSITY OF ROCHESTER RSRB
MAR 28 2007
EXPIRES

age (e.g., 24 weeks gestation plus 12 weeks of age = 36 weeks adjusted age).

Your baby will receive all standard care provided to any baby in the Neonatal Intensive Care Unit. No additional tests or procedures will be done on your baby just for this study. We will collect medical information regarding your baby's time in the hospital from your baby's medical chart. This will include laboratory values, any additional treatments that your baby receives and his or her progress over time.

We will continue to stay in touch with you and your infant by telephone every six months over the next 18 – 22 months. At these times, we will ask questions about your child's breathing (especially wheezing and coughing), medication use, and visits to the Doctor, Emergency Room or Hospital for treatment of breathing problems. We will also ask you several questions about your family and yourself. The telephone calls should take about 15 minutes of your time, less if your baby has had no breathing problems.

We will schedule the telephone calls at a time that is convenient for you. The telephone calls will occur when your infant is 6, 12, and 18 months after his/her expected delivery at full term delivery date.

The results of your baby's questionnaire will be combined with other infants from around the country. However, your baby's name will not be used.

We will ask you to bring your baby in for a clinic visit at 18 to 22 months. At the follow-up visit your baby will receive testing of his or her muscles, nerves and mental and motor skills. This clinic visit will take approximately 2-4 hours.

Optional part of the Study

Standard of care in our NICU includes doing head ultrasounds at 1 – 14 days of age, at approximately 6 weeks of age, and at other times as deemed necessary for your baby's care. If you agree, we would perform an additional test, a magnetic resonance imaging study (MRI) when your infant is at 35 – 42 weeks PMA, with a corresponding head ultrasound if one has not been done as standard of care. The MRI is a more advanced scan and can detect subtle differences in the brain that may not be seen on the head ultrasound. Your baby may or may not require light sedation while the MRI is being done.

Number of Subjects

Approximately 1300 babies will be enrolled in this study at 16 different centers across the country. Of those, approximately 50 will be enrolled at Golisano Children's Hospital at Strong.

Risks of participation

The procedures that are being used are standard (routine) treatments used in neonatal intensive care. Special attention will be given to prevent any damage to the mucosa (soft tissue) of the

UNIVERSITY OF ROCH[illegible]
MAR 28 200[illegible]
EXPIRES

nostrils (if your baby receives a tube in the nostril) and/or the windpipe (if your baby receives a tube in the windpipe). Premature babies receiving either CPAP or assisted ventilation are at risk for lung problems, including leakage of air outside the lungs and prolonged need for oxygen. It is not clear that one treatment has a lower risk of this than another. Early use of surfactant (like in the babies who will get assisted ventilation) protects premature babies from severe lung disease and death. Continuous use of CPAP also improves these same outcomes. It is not clear that one treatment has a lower risk of this than another. To the best of our understanding, there will be no more risks for the baby in this study than are possible for any ill premature baby needing intensive care.

The MRI does not use x-rays. If your baby needs to have sedation for the study, the risks include sleepiness, slow breathing, or cessation of breathing. The incidence of these complications is low and your infant will be carefully monitored.

It is possible that the procedures that your baby receives will be less effective or have more risks or complications of prematurity than the other treatment but this will not be known until the end of the study. The incidence of these complications will be carefully measured in this study. We will take every precaution to minimize potential complications.

Benefits of Participation

Your baby may or may not benefit from participation in this study.

New Study Findings

You will be informed of any new findings, which may affect your decision to continue your baby's participation in this research study. If you would like to know about the results of the research, please let Dr. Nirupama Laroia know; so that results can be provided on request at the completion of the study.

Alternatives

You may choose not to permit your child to participate in this study. If you choose not to enter your baby in this study, your baby will receive standard care. In that case, your baby's doctor will decide whether to administer nasal CPAP, oxygen or ventilation to your baby, and standard blood oxygen levels will be used.

Costs

You or your insurance company will be responsible for the cost of standard medical care that your baby receives. There will be no additional costs as a result of your baby's participation in this study. The follow up evaluation is provided at no cost and you and your child's physician will receive a full report.

Circumstances for Dismissal from the Study

You will be dismissed from the study if the study sponsor decides to stop or cancel the study.

UNIVERSITY OF ROCHESTER RSRB
MAR 28 2007
EXPIRES

Sponsor Support

The University of Rochester is receiving payment from the National Institute of Child Health and Human Development, a division of the National Institutes of Health, for conducting this research study.

Compensation for Injury

The University of Rochester will provide medical care for any emergency medical problem that your child may experience as a direct result from your child's participation in this research. You will not have to pay for this emergency care, but the university may seek reimbursement for this care from your health insurance carrier. Decisions regarding care and compensation for any research related injury will be made on a case-by-case basis.

Confidentiality of Records and HIPAA Authorization

While we will make every effort to keep information we learn about your baby private, this cannot be guaranteed. Other people may need to see the information. While they normally protect the privacy of the information, they may not be required to do so by law. Results of the research may be presented at meetings or in publications, but your baby's name will not be used.

The federal Health Insurance Portability and Accountability Act (HIPAA) requires us to get your permission to use health information about your baby that we either create or use as part of the research. This permission is called an Authorization. We will use your child's research record, related information from your child's medical records, results of laboratory and other diagnostic tests obtained during his/her initial hospitalization, as well as information obtained during the 18-24 month follow up visit.

We will use your child's health information to conduct the study, to monitor your child's health status and to determine outcomes related to the use of this therapy for treating respiratory disease. Health information is used to report results of research to sponsors and federal regulators. It may be audited to make sure we are following regulations, policies and study plans. If you have never received a copy of the Strong Health HIPAA Notice, please ask the investigator for one.

To meet regulations or for reasons related to this research, the study investigator may share a copy of this consent form and records that identify your baby with the following people: The Department of Health and Human Services, the University of Rochester, the National Institutes of Child Health and Human Development (NICHD) and organizations (like the Research Triangle Institute) used by the NICHD to manage studies.

If you decide to have your child take part, your Authorization for this study will not expire unless you cancel (revoke) it. You can always cancel this Authorization by writing to the study investigator. If you cancel your Authorization, your child will be removed from the study. However, standard medical care and any other benefits to which you are otherwise entitled will not be affected. Canceling your Authorization only affects uses and sharing of information after the study investigator gets your written request. Information gathered before then may need to

UNIVERSITY OF ROCHESTER RSRB
MAR 28 2007
EXPIRES

be used and given to others. For example, information gathered during your child's initial hospitalization may need to be sent to the NICHD Neonatal Research Network and to the Research Triangle Institute.

As stated in the section on Voluntary Participation below, you can also refuse to sign this permission/Authorization and not have your baby be part of the study. You can also tell us you want to withdraw your baby from the study at any time without canceling the Authorization. By signing this permission form, you give us permission to use and/or share your baby's health information.

Contact Persons

For more information concerning this research or if you believe that your child has suffered a research-related injury, please contact: Nirupama Laroia, MD (principal investigator) at (585) 275-2972.

If you have any questions about your rights as a research subject, you may contact the Human Subjects Protection Specialist at the University of Rochester Research Subjects Review Board, Box 315, 601 Elmwood Avenue, Rochester, NY 14642-8315, Telephone: (585) 276-0005; for long-distance, you may call toll-free, (877) 449-4441.

Voluntary Participation

Taking part in this study is voluntary. You are free not to participate or to withdraw at any time, for whatever reason. If you do decide to withdraw, we will keep confidential the information we have collected. If you choose not to have your child participate or you wish to withdraw your baby from the study, your baby will not risk loss of present or future care your baby would otherwise expect to receive.

Optional MRI

☐ I agree to have the MRI performed on my child.

☐ I do not agree to have the MRI performed on my child.

UNIVERSITY OF ROCHESTER RSRB
MAR 28 2007
EXPIRES

Signatures/Dates

I have read (or have had read to me) the contents of this permission form and have been encouraged to ask questions. I have received answers to my questions. I give permission for my child to participate in this study. I will receive a signed copy of this form for my records and future reference.

____

Study Subject (Print)

__________________________	_______________________	____________
Parent/Guardian Signature	Print Name	Date

Person Obtaining Consent

I have read this form to the parent/guardian of this subject and/or the parent/guardian of this subject has read this form. An explanation of the research was given and questions from the subject's family were solicited and answered to their satisfaction. In my judgment, the parent/guardian has demonstrated comprehension of the information. I will provide the parent/guardian with signed copy of this consent form.

___________________________	_______________________	_____________
Signature, person conducting Informed Consent	Print Name	Date

UNIVERSITY OF ROCHESTER RSRB
MAR 28 2007
EXPIRES

TUFTS MEDICAL CENTER
DEPARTMENT OF PEDIATRICS, DIVISION OF NEWBORN MEDICINE

INFORMED CONSENT FORM TO PARTICIPATE IN RESEARCH

The Surfactant Positive Airway Pressure and Pulse Oximetry Trial (SUPPORT) in Extremely Low Birth Weight Infants

Principal Investigator: Ivan D. Frantz III, MD
Co-investigator: John M. Fiascone, MD

INTRODUCTION

Your baby is invited to participate in a research study to compare two ways to assist premature babies to breathe and to compare two ranges of blood oxygen saturation levels by which premature babies are managed. You are being asked to allow your child to be in the study because (s)he might be born 12-16 weeks early (at 24 to 28 weeks of pregnancy). If your baby is born at or after 28 weeks of pregnancy, (s)he will not be enrolled in this study.

The study is being conducted by the National Institutes of Child Health and Human Development Neonatal Research Network, of which Tufts Medical Center is a member. Nationwide, a total of 1300 patients are expected to enroll in this study and we expect that about 30 of those infants will be from Tufts Medical Center. Children enrolled in the study will be involved for two years.

BACKGROUND

Babies born prematurely are at risk for breathing problems. A baby's lungs are made up of tiny air sacs. Each one is supposed to open and close as the baby breathes in and out. This works well in full term babies and adults; however, in premature babies the lung sacs don't always work this way. Some lung sacs open and close normally; others collapse and stick together when the baby breathes out making it harder to breathe.

Almost all babies born between 24-28 weeks of pregnancy require some treatment to help them breath. Doctors use one of two different treatment approaches. One approach is to give the baby a medication called surfactant shortly after delivery to try and keep the lung sacs expanded. Surfactant is given directly into the lungs via a breathing tube that is inserted into the airway (referred to as intubation). The baby is then maintained on a ventilator (breathing machine) until the baby may tolerate removal of the ventilator. A second treatment approach is to keep the lungs slightly inflated (or open) between breaths. This may be done by placing small tubes in the

Tufts Medical Center IRB # 7856
Approved 11 June 2008
Valid Until 12 May 2009

baby's nostrils to provide a small amount of continuous air pressure (CPAP) in the lungs to make it easier for the baby to take a breath. It is at present not clear if one of these approaches is better than the other.

Babies may also develop longer-term problems. The need for oxygen extending to four weeks prior to the baby's normal due date at full-term means your baby has a disease called Bronchopulmonary dysplasia (BPD), which is characterized by abnormal lung development. Premature babies are also at risk for an eye disease called Retinopathy of Prematurity (ROP). This problem affects the blood vessel growth in their eyes and may result in vision problems or even blindness. The amount of oxygen that a baby receives (oxygen saturation) may be a risk factor for ROP. The oxygen saturation is routinely monitored with a pulse oximeter. This oximeter uses a tiny probe, which is much like a band-aid applied to the foot or hand. There has not been consensus across the country about what is the best oxygen saturation range in which to keep premature babies, however the normal range of saturation is generally considered to fall between 85-95%.

Either treatment (CPAP or use of breathing tube, surfactant and breathing machine) is acceptable medical practice, with some hospitals favoring one or the other. One treatment has not been proven to be better than the other. Based on our interpretation of the medical literature, the standard medical treatment at Tufts Medical Center for babies born at less than 27 weeks gestational age is use of a breathing tube, breathing machine and administration of surfactant in the delivery room. Babies bom 27-28 weeks gestational age receive surfactant if they are intubated for any reason in the delivery room. At Tufts Medical Center oxygen saturation is kept between 88-94%.

PURPOSE OF STUDY

The primary purpose of this study is to evaluate the effect of two respiratory treatments and two ranges of blood oxygen saturation levels on the incidence and/or severity of BPD and ROP. We hope to determine if providing CPAP instead of ventilator support may reduce the incidence and/or severity of BPD and if a lower oxygen saturation range results in decreased ROP. Additionally, we will study two methods to detect brain injury and will follow your baby's growth and development. Specifically, this study will:

1. Compare two ways of assisting premature infants to breathe. Infants who receive CPAP in the delivery room with specific guidelines for further breathing support will be compared to infants who have a breathing tube placed, surfactant given in the delivery room, followed by breathing support.
2. Find out the oxygen level that should be used to help prevent some of the eye injury that may occur in premature babies. Specifically, infants in lower range (85-89%) and higher range (91-95%) oxygen levels (saturation) will be compared.

Tufts Medical Center IRB # 7856
Approved 11 June 2008
Valid Until 12 May 2009

3. Compare brain imaging by ultrasound or magnetic resonance imaging (MRI), done around the time when a baby would normally be born, to determine if one method gives more useful information about brain injury than the other.
4. Determine any effect of the study treatments on premature babies' growth and respiratory, neurological and developmental health during the first 18-22 months after his/her expected delivery at full term.

STUDY PROCEDURES

If you decide to allow your child to be in this study, a few minutes before your child is born, (s)he will be randomly assigned, like the flip of a coin, to one of two lung treatment strategies and one of two oxygen treatment levels. Your infant will be assigned to one of the four groups shown below. Each of the 4 possible combinations of treatments is considered by some hospitals to represent their desired standard approach. No one approach is known to be better than the others.

CPAP<br>Higher oxygen saturation	CPAP<br>Lower oxygen saturation
Surfactant + breathing machine<br>Higher oxygen saturation	Surfactant + breathing machine<br>Lower oxygen saturation

The treatments are as follows: 1) CPAP in the delivery room immediately after birth and continuing in the intensive care nursery (NICU), or 2) placement of a breathing tube in the windpipe in the delivery room followed by surfactant administration and ventilation (breathing for the baby using a machine). Infants randomized to the CPAP group may, at some point in their care, require a breathing tube and a breathing machine. If the attending physician deems this necessary, participation in the study will not affect this decision. If intubation occurs within the first 48 hours of birth, the baby will receive surfactant. Study guidelines for lung treatments of infants in both groups will be followed for two weeks.

In addition to being randomly assigned to one of the two groups described above, your baby will be randomly assigned to having an oximeter (blood oxygen monitor) which reads slightly high or one that reads slightly low. The oximeters used in this study are devices approved by the U.S. Government's Food and Drug Administration which have been modified for research purposes. This modification makes the monitors show an oxygen saturation value that is either a little higher or a little lower than the true oxygen reading when values are in the normal range

Tufts Medical Center IRB # 7856
Approved 11 June 2008
Valid Until 12 May 2009

(between 85 and 95%). Outside those ranges, the oximeter works the same as a standard oximeter. This will allow us to keep the saturations at the high or low end of the normal range and still protect the study infants from undesirable oxygen levels. The doctors and nurses taking care of your infant will not know if (s)he is in the high or low saturation group. This is to help assure that all patients are cared for in the same way. Your child will be on a study oximeter until about four weeks before the original "due date" or until transfer to a hospital not participating in this study. At that time, the oximeter will be changed to a standard one for the remainder of his/her hospital stay.

Other aspects of your infant's care will be the standard treatments for premature babies in the Tufts Medical Center NICU. Information from your baby's medical record about medical care your baby received and results of tests and data that pertain to your baby will be documented in your baby's research study records. This information will include respiratory support; feeding and weekly growth measurements of body weight, length, and head circumference; and results of eye exams.

Your baby will have routine ultrasounds of his/her head during his/her stay in the NICU. A copy of the head ultrasounds conducted between 4-14 days of life and at the time of the original expected due date will be collected for this study. If your baby's head ultrasound at the time of the expected due date occurs before 35 weeks post-menstrual age, your baby will receive another head ultrasound for the purpose of this study between 35-42 weeks post-menstrual age. In addition, your baby will have a MRI (Magnetic Resonance Imaging) for the purpose of this research study between 35-42 weeks post-menstrual age._Neither the head ultrasound nor the MRI is experimental and both are currently used on infants at Tufts Medical Center. The ultrasound shows the structure of your baby's brain by bouncing sound waves off of it. You may have seen an ultrasound of your baby during your pregnancy. The MRI is a specialized brain scan that takes detailed pictures of the brain structure and can detect normal and abnormal brain tissue. If conducted for the purpose of this study, the head ultrasound and/or MRI will be paid for by The National Institutes of Child Health and Human Development at no cost to you. If your baby is still in the NICU, your baby will need to be transported to the MRI suite in order to have the procedure. Only those patients considered stable for transport will undergo an MRI. If your baby is discharged home prior to 35-42 weeks PMA, you will be asked to return to Tufts Medical Center for the head ultrasound and MRI procedures. If your baby is transferred to another hospital participating in this study, the head ultrasound and/or MRI may take place at that hospital. The MRI will be conducted after your baby is swaddled while sleeping following a feeding and with the use of a jacket-like device that allows the baby to lie still without using sedation. A physician at the hospital in which you receive the head ultrasound and MRI will provide you with an interpretation of the results of the tests. Neither the ultrasound nor the MRI involves exposure to radiation.

Tufts Medical Center IRB # 7856
Approved 11 June 2008
Valid Until 12 May 2009

At the time your baby is discharged from the NICU, you will be asked questions about your socioeconomic status such as how much money your family earns, the level of education you have completed, and information about your household. You will also be asked questions about any family history of breathing problems or respiratory illness.

All children in the Tufts Medical Center NICU return to the NICU Follow-up Clinic at regular intervals during the first two years as part of their routine medical care. We will continue to stay in touch with you by telephone or in person at these visits over the next 18-23 months. At these times, we will ask questions about your child's breathing (especially wheezing and coughing), medication use, and visits to a doctor, emergency room, or hospital for treatment of breathing problems. We will also ask you several questions about your family and yourself. Each interview should take 15 minutes or less of your time. When your baby returns to the NICU follow-up clinic for his/her 18-month-old assessment of growth, development, and coordinated movement skills, the study will collect that outcome information. In order to successfully evaluate the approaches to lung treatment and oxygen used in this study, follow up at 18-22 months is essential for this study. Families who participate in this project are agreeing to remain in contact with the investigators and to return to the NICU Follow-up clinic with their child when (s)he is 18 months of age.

Research procedures for this study occur while your baby is in the hospital and if discharged home prior to 35 weeks post-menstrual age, when your baby returns to the hospital for a head ultrasound and/or MRI. Data will be collected for this study at the 18-month follow-up visit that your child attends as part of standard medical care. Subjects in this study must participate in all aspects of the study including the MRI and head ultrasounds and 18-month follow-up visit.

Doctors working on the SUPPORT study hope that it will be possible to follow the children who participated in SUPPORT for more than 18-22 months. If money for longer follow-up is obtained, we would like to follow your child for up to 6-7 years. If money for longer follow up is obtained we ask your permission for the study doctor and/or his designee to contact you about possible additional years of follow-up. Your child could only participate in SUPPORT follow-up beyond 18-22 months with your written consent, but you would not be under any obligation to continue.

RISKS AND DISCOMFORTS

This study does not pose significant risks beyond those inherent in a sick premature baby. For example, premature infants with respiratory distress syndrome are at increased risk of pneumothorax (collapse of lung) however there is no evidence supporting a difference in this risk between the respiratory treatments being studied (breathing tube and breathing machine v. CPAP

Tufts Medical Center IRB # 7856
Approved 11 June 2008
Valid Until 12 May 2009

in the delivery room). It is not known if the known reduction of death and disease severity as a result of surfactant in the delivery room and the reduction of BPD in babies that receive surfactant will be less than, offset, or increased by the early use of CPAP. Infants treated with CPAP often swallow air and have a tube placed into the stomach to remove it None of the aspects of the study are believed to be uncomfortable beyond the discomfort associated with routine medical care. All treatments are standard of care at some NICUs across the country.

There are no known effects from exposure to magnetic fields (MRI). Temporary minor skin irritation from tape used to apply MRI-compatible monitoring electrodes may occur, but this risk is unlikely.

Some unknown risks may be learned during the study. You will be told of any new information that is learned which may affect your child's condition or influence your willingness to have him/her continue participation in this study.

Another risk of this study is the risk to confidentiality. Every effort will be made to keep your child's medical record confidential. There will be no name or other patient identification in any study report that may be published after the study is completed. Measures taken to protect you and your baby's identity are described in the confidentiality section of this document.

BENEFITS

There may be benefits to your child directly, including a decrease in chronic lung disease and/or a decrease in the need for eye surgery as a result of exposure to oxygen. Because we do not know in advance the actual strategies chosen for your child, or which of study groups is the most effective, it is also possible that your baby will not receive these possible benefits. However, all babies in the study may benefit from the MRI that is conducted for purposes of this research. Babies may benefit from this procedures if they identify brain injury, which may allow for earlier treatment than would normally occur. If the doctor sees anything on the MRI that would help treat your infant, s/he will use that information.

ALTERNATIVES TO PARTICIPATION

The alternative to having your child participate in this project is not to participate. If you choose not to have your child participate, he/she will receive the standard care for premature infants.

CONTACT INFORMATION

If you have any questions about the study or concerns during the study, you may contact the investigator (Dr. Frantz) or his designees via the Tufts Medical Center Paging Operator: (617)

Tufts Medical Center IRB # 7856
Approved 11 June 2008
Valid Until 12 May 2009

636-5114 or Tufts Medical Center (617) 636-5008. If you have question about your rights as a research study subject, call the Tufts Medical Center Institutional Review Board (IRB) at (617) 636-7512. The Institutional Review Board is a group of doctors, nurses, and non-medical people who review human research studies for safety and protection of people who take part in the studies. Federal law requires the Institutional Review Board to review and approve any research study involving humans. This must be done before the study can begin. The study is also reviewed on a regular basis while it is in progress. This research study has been reviewed and approved by the IRB of Tufts Medical Center.

RESEARCH RELATED INJURY

All forms of medical diagnosis and treatment, whether routine or experimental, involve some risk of injury. In spite of all precautions, your child might develop medical complications from participating in this study. If such complications arise, immediate necessary medical care is available at Tufts Medical Center. Either you or your health insurance will be responsible for the costs of medical care that is medically necessary or indicated for your infant.

COSTS

There is no extra cost to participate in this study beyond the costs of medical care that is medically necessary or indicated for your infant's care and which is the responsibility of you or your health insurance. The head ultrasound and MRI conducted for the sole purpose of research is paid for by the National Institutes of Child Health and Human Development, which is the sponsor of this study.

PAYMENT

If your baby is discharged home prior to the MRI and head ultrasound procedures at 35-42 weeks post-menstrual age, you will be reimbursed reasonable travel expenses to get to these appointments. A gift certificate to a local department store for $20.00 will be provided per child at the 18-month follow-up visit as an appreciation payment for your child's participation.

VOLUNTARY PARTICIPATION/WITHDRAWAL

Taking part in this research study is completely your choice and you are free to change your mind. You can decide to stop taking part in this study at any time for any reason and your child will receive standard medical care. If you want to stop participation in this study, we ask that you contact Dr. Ivan D. Frantz III by writing, telephone or in person and let him know that you are withdrawing your child from the study. Dr. Frantz can be reached via telephone through the Tufts Medical Center Paging Operator: (617) 636-5114 or Tufts Medical Center NICU (617)

Tufts Medical Center IRB # 7856
Approved 11 June 2008
Valid Until 12 May 2009

636-5008. His mailing address is 800 Washington Street, Tufts Medical Center #44, Boston, MA 02111. At that time we will ask your permission to continue using data that has already been collected as part of the study prior to your withdrawal. At the discretion of the Principal Investigator, subjects may be taken out of this study due to unanticipated circumstances. Examples of reasons for taking a participant out of the study include: the investigator deciding that continued participation could be harmful to your child, the study being canceled, or some other administrative reason.

CONFIDENTIALITY

Extensive efforts are made to protect all research subjects from the use of information that will adversely affect them. Specifically, access to information about you and your child is restricted to the Tufts Medical Center Division of Newborn Medicine clinical research staff that is involved in this study. Clinical and research information with respect to this study is maintained in a research file separate from hospital medical records and will not be placed in the official Tufts Medical Center medical record by research staff. Clinical information collected from your baby's chart for this study will be labeled with a coded study ID number. Coded information will be sent to the NICHD Neonatal Research Network's Data Coordinating Center at Research Triangle Institute (RTI) in Research Triangle Park, North Carolina. The key linking the code number with your baby's identity will be kept secure at the Tufts Medical Center Division of Newborn Medicine clinical research office. Your baby's head ultrasound and MRI images may be identified with a header listing you baby's name, hospital number and birth date since it is not always possible to remove your baby's identification from these scans. These scans will be sent to RTI so that study radiologists may read the image and ensure consistent interpretation across all of the babies around the country that are participating in this study. The study radiologists will not have any other information about your child. In no other instance, will information directly identifying your baby, such as name or medical record number, to study data collected leave Tufts Medical Center. Research data from which your child may be identified will not be disclosed to third parties except with your written permission or as required by law. If research results from this study are reported in a professional setting, such as in a medical journal or at a scientific meeting, the identity of research subjects taking part in the study is withheld.

Tufts Medical Center IRB # 7856
Approved 11 June 2008
Valid Until 12 May 2009

PARTICIPANT'S STATEMENT

I have read this consent form and have discussed with Dr. Frantz or his/her representative the procedures described above. I have been given the opportunity to ask questions, which have been answered to my satisfaction. I understand that any questions that I might have will be answered verbally or, if I prefer, with a written statement.

I understand that I will be informed of any new findings developed during the course of this research study that may affect my willingness to stay in this research study.

I understand that my participation is voluntary. I understand that I may refuse to participate in this study. I also understand that if, for any reason, I wish to discontinue participation in this study at any time, I will be free to do so, and this will have no effect on my future care or treatment by my physicians or this hospital.

I understand that in the event I become ill or am injured as a result of participating in this research study, medical care will be provided to me. However, such medical care will not be provided free of charge, even if the injury or illness is a direct result of this research study. I understand that no funds to provide financial compensation for research-related injury or illness are available.

If I have any questions concerning my rights as a research subject in this study, I may contact the Institutional Review Board at (617) 636-7512.

I have been fully informed of the above-described study with its risks and benefits, and I hereby consent to the procedures set forth above.

I understand that as a participant in this study my identity and my medical records and data relating to this research study will be kept confidential, except as required by law, and except for inspections by the U.S. Food and Drug Administration which regulates investigational drug studies, and the study sponsor.

____________ ____

Date One Parent/Legal Authorized Representative Signature

I have fully explained to ______________________________ the nature and purpose of the above-described study and the risks that are involved in its performance. I have answered all questions to the best of my ability.

____________ ____

Date Principal Investigator or Representative's Signature

Tufts Medical Center IRB # 7856
Approved 11 June 2008
Valid Until 12 May 2009

SHARP Mary Birch Hospital for Women

INFORMED CONSENT

The Surfactant Positive Airway Pressure and Pulse Oximetry Trial in Extremely Low Birth Weight Infants

The SUPPORT Trial of the NICHD Neonatal Research Network

IRB # 041093

Revised August 1, 2008

APPROVED

SEP 1 9 2008

[illegible] Review Board
[illegible]

PRINCIPAL INVESTIGATOR:

Maynard Rasmussen, MD

SPONSOR:

National Institute of Child Health and Human Development (NICHD)
Neonatal Research Network

(858) 939-3400 ■ 3003 Health Center Drive ■ San Diego, California 92123

APPROVED

SEP [illegible] 2008

Institutional Review Board
Sharp HealthCare

The Support Trial of the NICHD

Participation in a Research Study

The Neonatologists at Sharp Mary Birch Hospital for Women (SMBHW) and the National Institute of Child Health and Human Development (NICHD) Neonatal Research Network are conducting a research study in extremely premature infants to find out more about:

1) Treatment with Continuous Positive Airway Pressure (CPAP) - pressure applied with a face mask or nasal prongs to help babies breathe easier and keep their lungs from collapsing
2) The ideal range of Oxygen Saturation - the amount of oxygen in the blood

You are being asked to allow your baby to be in this study because there is a possibility he/she will be born 12 to 16 weeks early (24-28 weeks gestation). We need to tell you about the study so you can decide if you want your baby to participate. You need to know why we are doing the study, if there might be any risks for your baby, and what we will expect from you and your baby.

Why is this Study Being Done?

1) To compare the treatment of extremely premature infants in the delivery room who receive CPAP with infants who are treated with a breathing tube in the windpipe (trachea) and surfactant (a medication which keeps the lungs from collapsing) to see if either treatment results in healthier lungs.
2) To compare treatment with lower oxygen saturation range (85-89%) with higher range (91-95%) to determine if either range results in healthier eyes.

Both of these approaches may be beneficial. This study will help determine which of these treatments work the best and are the safest for premature infants.

A number of studies have suggested that the early use of CPAP and the avoidance of mechanical ventilation (assisting the baby's breathing with a breathing machine) are associated with:

1) A decreased need for treatment with surfactant (a natural substance extremely premature infants lack and which helps keep the lungs from collapsing)
2) A decreased risk of death or need for oxygen at 36 weeks post-conceptional age (for a baby born at 24 weeks gestation and who is 12 weeks old, his/her post-conceptional age is 36 weeks)

Currently the most extremely premature infants are treated with mechanical ventilation and surfactant. Many studies have shown that surfactant therapy is associated with:

1) A decreased risk of the lungs collapsing
2) A decreased risk of developing leaks in the lungs
3) A decreased risk of death or needing oxygen at 36 weeks post-conceptional age

Retinopathy of Prematurity (ROP) is a common eye problem in extremely premature infants, because the blood vessels that nourish their eyes have not fully developed. ROP may result in

APPROVED

SEP 1 0 2008

The Human Research Review Board
Sharp HealthCare

impairment of vision or even blindness, which may be caused by excessive levels of oxygen. Blood oxygen saturation levels in premature infants are commonly maintained between 80-95%. The ideal oxygen saturation in these extremely low birth weight infants (ELBW) is unknown. This study will compare the outcomes of infants whose oxygen saturation target is 85-89% vs. those whose target is 91-95% to see if either target decreases the risk of ROP.

How Many Infants will be Enrolled?

We plan to enroll approximately 1300 babies at NICHD Neonatal Research Network hospitals over a two-year period. Approximately 50 infants will be enrolled here at SMBHW.

What Does Participation in This Study Involve?

If you agree for your baby to be in this study, the following will happen to him/her: Prior to delivery, your baby will be randomly assigned (chosen by chance like a flip of a coin) to one of two treatment strategies. The treatments are as follows:

1) CPAP in the delivery room immediately after birth and continuing in the NICU

Or

2) Placement of a breathing tube in his/her trachea (windpipe) in the delivery room followed by surfactant administration and mechanical ventilation (breathing for the baby using a machine).

Your baby will also be randomly assigned to a study oximeter (an oxygen saturation monitor that displays how much oxygen is in the blood). These oximeters will assist us in maintaining your baby's oxygen saturation target. It will also record your baby's oxygen saturations until he/she reaches 36 weeks post-conceptional age or no longer needs extra oxygen.

Routine neonatal intensive care will be provided during your baby's participation in the study. Your baby will be followed in our Infant Follow-up clinic at 6 and 12 months as standard of care for small babies. At 18-22 months corrected age your baby will receive a complete exam of his/her muscles, nerves, and mental and coordinated movement skills.

How Long Will Your Baby be in the Study?

Your baby will be in the study until 18-22 months post-conceptional age.

What are the Risks of the Study?

Each of the study treatments is already being used by many doctors across the country, there is no predictable increase in risk for your baby. Infants randomized to the CPAP group may, at some point in their care, require intubation and assisted ventilation (methods to help them breathe). If the attending physician deems this necessary, participation in the study will not affect this decision.

APPROVED

[illegible]

[illegible]

Some unknown risks may be learned during the study. If these occur, study personnel will inform you.

The only other risk of the study is confidentiality. Every effort will be made to keep your baby's medical record information confidential. Your baby will not be identified in any report or publication about this study. Measures taken to protect you and your baby's identity are described in the confidentiality section of this document.

Are There Benefits to Taking Part in the Study?

Your baby may or may not receive any direct benefit from taking part in this study. However, your participation in this study may help us learn about better ways to treat babies requiring assistance with breathing.

What about Confidentiality?

Study personnel at SMBHW will collect clinical information from your baby's chart. Information will be labeled with a code number. Coded information will be sent to the NICHD Neonatal Network's Data Collection Center at Research Triangle Institute at Research Triangle Park, North Carolina. The study log linking the code number information with your baby's identity will be kept under lock and key at SMBHW. Research records will be kept confidential to the extent provided by law.

Organizations that may inspect and/or copy your baby's research records for quality assurance and data analysis include the following groups: the United States Food and Drug Administration (FDA), the Sharp HealthCare Institutional Review Board (IRB), the study sponsor NICHD Neonatal Research Network, and the Office for Human Research Protections (OHRP).

Protected Health Information (PHI)

As a part of this research study you will be asked to sign a separate document giving your permission to use and disclose your baby's medical records. This document will tell you who will view your baby's records, how they will be used and how long they will be needed. It will also tell you what you can do if you no longer agree to have your baby's medical records used. Your signature will give us permission to use your baby's records. You will receive a copy of the signed document.

What are the Costs?

It is anticipated that taking part in this study will <u>not</u> lead to added costs to you and your insurance company. The costs of diagnostic tests and the treatment of your baby will be your responsibility and/or that of your insurance carrier. Sharp HealthCare and the investigators will be partially reimbursed by the sponsor for time, effort and oversight by the professional staff to perform procedures, tasks, and accurately collect and submit data.

APPROVED
[illegible]
[illegible]

Research Related Injury?

In the case of injury or illness resulting from this study, emergency medical treatment is available but will be provided at the usual charge. No funds are available to compensate you in the event of injury. Sharp HealthCare will not provide any compensation to you in the event your baby sustains a research related injury while participating in this study.

What are My Rights as a Participant?

Taking part in this study is voluntary. You may choose for your baby not to take part or may leave the study at any time. Leaving the study will not result in any penalty or loss of benefits to which you or your baby are entitled. A Data Safety and Monitoring Board, an independent group of experts, will be reviewing the data from this research throughout the study. We will tell you about the new information from this or other studies that may affect your baby's health, welfare, or your willingness to keep your baby in this study.

What Alternatives to the Study do I Have?

As an alternative to participation in this study you may have the baby's doctor decide which treatment your baby will receive. If you decide not to include your baby in this study, none of his/her medical information will be included in the study. Participation in research is entirely voluntary. You may refuse for your baby to participate or withdraw at any time without jeopardy to the medical care your baby will receive at Sharp Mary Birch Hospital for Women or loss of benefits to which your baby is entitled. If you withdraw your baby from the study, the attending physician will decide whether to maintain the current treatment or to change it, based on your baby's needs at the time of the decision. Data collection for research purposes will stop at that time.

Your baby's participation in this study may be stopped if the doctor feels that it is in your baby's best interest medically, or if the FDA or the IRB stops the study.

Who do I Call If I Have Questions or Problems?

For questions about the study or a research related injury, contact the principal investigator, Maynard Rasmussen, MD, at (858) 939-4176.

For questions about your rights as a research participant or to address complaints about the research, contact David J. Bodkin, M.D., Chair of the Sharp HealthCare Institutional Review Board (a group of people who review the research to protect your rights) via the:

Sharp's Office for the Protection of Research Participants (IRB)
8695 Spectrum Center Boulevard
San Diego, California 92123
Phone: (858) 499-4836

APPROVED

Signature

Your signature below indicates that you have read the above about the SUPPORT Trial and have had a chance to ask questions to help you understand what your baby's participation will involve. You agree for your baby to participate in the study until you decide otherwise. You are not waiving your legal rights by signing this consent form.

____

Signature of Parent **Printed Name** **Date**
(or Legally Authorized Representative)

____

Signature of Witness **Printed Name** **Date**

I ______________________________ attest that the requirements for informed consent for the medical research project described in this form have been satisfied – that the participant's parent has been provided with a copy of the California Experimental Subject's Bill of Rights, that I have discussed the research project with the participant's parent and explained to him or her in non-technical terms all of the information contained in this informed consent form, including any risks and adverse reactions that may reasonably be expected to occur. I further certify that I encouraged the participant's parent to ask questions and that all questions asked were answered.

____

Signature of Investigator **Printed Name** **Date**

DTF1200

PATIENT INFORMATION

University of California, San Diego

Parent Informed Consent

The Surfactant Positive Airway Pressure and Pulse Oximetry Trial in Extremely Low Birth Weight Infants

The SUPPORT Trial of the NICHD Neonatal Research Network

This is a research study. Research studies include only subjects who choose to take part. You are being asked to to allow your child to be in the study because there is a possibility he/she will be born between 16 and 12 weeks early (24-28 weeks gestational age). Please take your time to make your decision. Discuss it with your family. Be sure to ask any questions that you may have.

STUDY INVESTIGATOR AND SPONSOR

Investigator(s): Neil Finer, MD
Sponsor: **Eunice Shriver National Institute of Child Health and Human Development**

WHY IS THIS STUDY BEING DONE?

This reasons this study is being done are:

1) To compare infants who receive delivery room CPAP (Continuous Positive Airway Pressure – a **commonly used method of keeping babies lungs expanded with increased airway pressure)** and who have strict guidelines for having a breathing tube placed with infants who have the tube placed and surfactant (a liquid which helps babies with immature lungs breath easier by helping keep their lungs from collapsing) given in the delivery room.
2) To compare low range (85-89%) oxygen saturation levels with high range (91-95%) levels to determine if a lower range results in decreased ROP (Retinopathy of Prematurity, an eye disease that may result in impairment of vision or even blindness, which may be

5-1-08
5-1-08
4-30-09
7pgs

caused by excessive levels of oxygen.)

WHAT MAKES THIS DIFFERENT FROM THE USUAL TREATMENT?

The use of CPAP and Intubation/Surfactant are both treatments currently used in the delivery room at UCSD. The decision as to which to use is currently made by the physician attending the delivery.

The oxygen level currently used in the NICU at UCSD is between 85% and 95%. Both treatment groups (85-89% and 91-95%) fall within that range. The study will attempt to keep babies in one of these two smaller ranges.

HOW MANY PEOPLE WILL TAKE PART IN THE STUDY?

50 subjects will be in this study at UCSD, and a total of 1310 nationwide at 16 other academic medical centers.

HOW LONG WILL YOUR CHILD BE IN THE STUDY?

Your child will be in the study for 18-22 months. The part of the study which uses either CPAP or Intubation and Surfactant lasts for 14 days. The part which keeps your baby in one of two levels of oxygen continues until your baby no longer need oxygen, or reach 36 weeks adjusted age. (A baby who was born at 24 weeks gestation will reach 36 weeks adjusted age 12 weeks after birth.) The follow-up part of the study that will see how your baby is doing developmentally will take place at 18-22 months. The NICHD is currently considering following infants in the SUPPORT trial until school age (6-7 years). This consent will include your permission to contact you at that time to re-evaluate you child's development. Because this will occur for some children after 7 years of age, we would also ask your child for their assent at that time.

You can stop your child's participating at any time. However, if you decide to stop your child from participating in the study, we encourage you to talk to the research doctor.

WHAT IS INVOLVED IN THE STUDY?

This is what will happen if your child participates in this study:

Prior to delivery, and after your permission, your baby will be randomized (chosen by chance like the flip of a coin) to one of two lung treatment strategies. The treatments are as follows:
1) CPAP in the delivery room immediately after birth and continuing in the NICU, or
2) The placement of a tube in his/her trachea (windpipe) in the delivery room followed by

surfactant administration and ventilation (breathing for the baby using a machine)
And,
In addition to being randomly assigned to one of the two groups described above, your baby will be randomized to a High reading or Low reading oximeter [a monitor that displays how much oxygen is in the blood.] The oximeters (oxygen monitors) used in this trial are FDA approved oximeters which have been modified for research purposes. This modification makes the monitors show a value which is either slightly higher or slightly lower than the true oxygen level when values are between 85 and 95%. Outside those ranges, the oximeter works the same as the standard of care device.
Which group your baby is randomized to will not be known to the nurse taking care of your baby, or his/her physician. Only the study coordinator will know which group your baby is in. Within the range of oxygen which we normally keep babies in, your baby will either be on the high end of normal or the low end of normal. He/she will remain on this device until he/she reaches 36 weeks adjusted age. (e.g. 24 wks gestation plus 12 weeks of age = 36 weeks adjusted age.) Other care will be conducted as normal during his/her participation in the study.

Your baby will be followed in our Infant Follow-up clinic at 6 and 12 months as standard of care for small babies. At 18-22 months corrected age your baby will receive, at no charge to you, a complete exam of their muscles, nerves, and mental and coordinated movement skills.

We will continue to stay in touch with you and your infant by telephone or in person at one of your visits every 6 months over the next 18-22 months, a total of three times.

WHAT ARE THE RISKS OF THE STUDY?

Participation in this study may involve some added risks or discomforts. Because all of the treatments proposed in this study are standard of care, there is no predictable increase in risk for your baby. Infants randomized to the CPAP group may, at some point in their care, require intubation and assisted ventilation (methods to help them breathe). If the attending physician thinks this is necessary, participation in the study will not affect this decision. Some unknown risks may be learned during the study. If these occur, you will be informed by the study personnel. The only other risk of this study is the risk to confidentiality. Every effort will be made to keep your child's medical record confidential. There will be no name or other patient identification in any study report that may be published after the study is completed. Measures taken to protect you and your baby's identity are described in the confidentiality section of this document.

ARE THERE BENEFITS TO TAKING PART IN THE STUDY?
There may be benefits to your child directly, including a possible decrease in chronic lung disease (need for extra oxygen near discharge) and/or a decrease in the need for eye surgery as a result of exposure to oxygen. Because we do not know in advance the actual

strategies chosen for your child, or which of the treatment strategies is the most effective, it is also possible that your baby will receive no direct benefit. The knowledge learned from this study may help us treat babies in the future. However, as noted above, each of the 4 possible combinations of treatments is considered by some units to represent their desired approach.

WHAT OTHER OPTIONS ARE THERE?

As an alternative to participation in this study you may decide to have your baby's doctor decide which treatment your baby will receive. If you decide not to include your child in this study, none of his/her medical information will be included in the study data. Participation in research is entirely voluntary. You may refuse to participate or withdraw at any time without jeopardy to the medical care your child will receive at this institution of other loss of benefits to which your child is entitled. If you withdraw your child from the study, the attending physician will decide whether to maintain current treatment or change it, based on your child's needs at the time of the decision. Data collection for research purposes will stop at that time.

CAN YOUR CHILD BE REMOVED FROM THE STUDY WITHOUT YOUR CONSENT?

Your child's participation in this study may be ended without your consent by the investigator or your baby's doctor if it is in your child's best medical interest, there is a lack of effect, or for other reasons. If your newborn leaves the study early, he/she will continue with whichever treatment the doctor feels is best.

WHAT ABOUT CONFIDENTIALITY?

Every reasonable effort will be made to keep your child's records confidential. Clinical information will be collected from your baby's chart by study personnel at UCSD. Information will be labeled with a code number. Coded information will be sent to the NICHD Neonatal Network's Data Collection Center at Research Triangle Institute (RTI) in Research Triangle Park, North Carolina. The study log linking the code number with your baby's identity will be kept under lock and key at UCSD. Information directly identifying your baby will not leave UCSD. Research records will be kept confidential to the extent provided by law.Your child's records and information will not be released without your consent to the extent the law allows. If the study results are published or presented, your child will not be identified.

WHAT ARE THE COSTS?

There are no costs to participate in this trial.

WHAT IF YOUR CHILD IS INJURED IN THE STUDY?

If your child is injured as a direct result of participation in this research, the University of California will provide any medical care your child needs to treat those injuries. The University will not provide any other form of compensation to you if your child is injured. You may call the UCSD Human Research Protections Program office at (858) 455-5050 for more information about this, or to inquire about your rights as a research subject, or to report research-related problems.

_______________ has explained this study to you and answered your questions. If you have other questions or research-related problems, you may reach Wade Rich, the Study Coordinator, or Renee Bridge, the Research Nurse, at 619-543-3759. You may contact the principal investigator Dr. Neil Finer at 619-543-3794

WILL YOU OR YOUR CHILD BE COMPENSATED?

Neither you nor your child will receive compensation for participation in this trial.

WHO DO YOU CALL IF YOU OR YOUR CHILD HAVE QUESTIONS OR PROBLEMS?

For questions about the study or a research-related injury, contact the researcher:

Neil Finer, MD
619-543-3794

WHAT ARE YOUR CHILD'S RIGHTS AS A RESEARCH SUBJECT?

Taking part in this study is voluntary. You may choose not to let your child take part or you or your child may choose to leave the study at any time. Your decision will not result in any penalty or loss of benefits to which your child is entitled. If you have questions about your child's rights you may call:

University of California, San Diego
Human Research Protections Program
(858) 455-5050

You will be told about any new information that may affect your child's health, welfare, or willingness to stay in this study.

SIGNATURE AND CONSENT TO BE IN THE STUDY:

Your signature below means that you have read the above information about the ____________ study and have had a chance to ask questions to help you understand

what your child will do in this study and how your child's information will be used.

You or your child can change your minds later if you want to. You will be given a copy of this consent form and a copy of the Subject's Bill of Rights. By signing this consent form you are not giving up any of your or your child's legal rights.

NAME OF PARTICIPANT	AGE
SIGNATURE OF PARENT OR GUARDIAN	DATE
SIGNATURE OF 2nd PARENT OR GUARDIAN	DATE
SIGNATURE OF WITNESS (person explaining this form)	DATE

INTERMOUNTAIN HEALTH CARE
IHC

PARENTAL PERMISSION and
AUTHORIZATION DOCUMENT
IHC INSITUTIONAL REVIEW BOARD

INTERMOUNTAIN HEALTH CARE
URBAN CENTRAL REGION
IRB
FEB 3 2009 APPROVED
DEC 11 2009 EXPIRATION DATE

TITLE: The Surfactant Positive Airway Pressure and Pulse Oximetry Trial in Extremely Low Birth Weigh Infants (SUPPORT Trial)

PRINCIPAL INVESTIGATOR: Bradley A. Yoder, MD (801) 581-7052

CO-INVESTIGATOR(S): Roger Faix, MD (801) 581-7052
Susan Wiedmeier, MD (801) 408-3435

LOCATION: Intermountain Medical Center and Primary Children's Medical Center

BACKGROUND:

You are being asked to allow your baby to be in this research study because there is a possibility he/she will be born between 24 and 27 weeks gestation. Before you decide it is important for you to understand why the research is being done and what it will involve. Please take time to read the following information carefully and discuss it with friends, and relatives if you wish. Ask us if there is anything that is not clear or if you would like more information. Please take time to decide whether or not you volunteer your baby to take part in this research study.

Our hospital is conducting this study in cooperation with the National Institutes for Child Health and Human Development (NICHD) Neonatal Research Network to try to determine the best way to manage early breathing support in the extremely premature infant and to determine the most appropriate level of oxygen in the blood of extremely premature babies.

STUDY PROCEDURES:

Prior to delivery, and after your permission, your baby will be randomized (chosen by chance, like a flip of a coin) to one of four treatment strategies as shown below. There are two breathing support strategies: 1) early CPAP (giving pressurized gas by small plastic tubes in the nose) and 2) Intubation/Surfactant (placing a breathing tube and giving a medicine through the tube to try to help the lungs work better). Both treatments are currently used immediately after delivery at this hospital. The decision as which to use is currently made by the physician attending the delivery. There are also two oxygen support strategies: 1) a low normal range (85-89%) and 2) a high normal range (91-95%). The box on the following page indicates the four possible treatment combinations your infant can be randomized to. There is an equal chance (1 in 4) for randomization to each treatment group.

CPAP<br>and<br>Higher oxygen saturation	CPAP<br>and<br>Lower oxygen saturation
Breathing tube + Surfactant<br>and<br>Higher oxygen saturation	Breathing tube + Surfactant<br>and<br>Lower oxygen saturation

Your baby will remain on a specially modified oxygen saturation monitor (measures oxygen levels in the blood through the skin without needle sticks) until he/she reaches 36 weeks corrected age (example: 24 weeks gestation plus 12 weeks of age = 36 weeks corrected age) or until the monitor is no longer needed because your baby is in room air. Because of the design of these monitors none of the nurses, doctors, or study personnel taking care of your baby will know if he/she is in the lower or higher oxygen saturation group.

Other care will continue as normal during his/her participation in the study.

A secondary purpose to this study is to determine the longer term effect of different approaches to breathing and oxygen support in the very premature baby. In that regard, this study includes three planned follow-up evaluations of all study baby's during the first two years of life including:

a. follow-up for subsequent lung problems

b. follow-up of neurodevelopmental function (a complete exam of their muscles, nerves, mental and coordinated movement skills) at 18-22 months age

c. a comparison of currently applied radiology studies (MRI and Ultrasound) obtained at 36-42 weeks corrected gestation for predicting later neurodevelopmental function

Some of these follow-ups may be by phone and some may be in our special follow-up clinic for very premature infants routinely provided at 6, 12 and 18-22 months age.

Our study coordinator will keep track of how your baby is doing up to 40 weeks gestational age (the babies original due date) or until discharge. Once your baby reaches 36 weeks gestational age we will record if he/she is still being treated with oxygen and/or a ventilator. If your baby is still on nasal cannula oxygen we will try to wean him/her off the oxygen using a standard protocol. If he/she successfully weans off oxygen, your baby's medical team may decide to take him/her completely off oxygen.

INTERMOUNTAIN HEALTH CARE
URBAN CENTRAL REGION
IRB

FEB 3 2009 APPROVED

DEC 1 1 2009 EXPIRATION DATE

RISKS:
Because all treatments proposed in this study are currently accepted standard of care, there is no predictable increase in risk to your baby. Infants randomized to the CPAP group may, at some point in their care, require intubation and assisted breathing. This will be determined by your baby's attending physician and participating in this study will not effect this decision.

BENEFITS:
We cannot promise any direct benefits to your baby from being in this study. However, possible benefits to your child may include decrease in chronic lung disease (need for extra oxygen at discharge) and decrease in the need for eye surgery as a result of exposure to oxygen. The information we learn from this study may help us treat premature babies in the future.

ALTERNATIVE PROCEDURES:

As an alternative to participation in this study you may decide to have your baby's doctor decide which treatment your baby will receive. If you decide not to include your baby in this study, none of his/her medical information will be included in the study data. Participation in research is entirely voluntary. You may refuse to participate or withdraw at any time without jeopardy to the medical care your baby will receive at this institution. Data collection for this study will stop at that time.

INTERMOUNTAIN HEALTH CARE
URBAN CENTRAL REGION
IRB

FEB 3 2009 APPROVED
DEC 11 2009 EXPIRATION DATE

PERSON TO CONTACT:

If you have concerns or questions about this research or any related matter, you can contact the primary investigator, Dr. Bradley Yoder @ 801-581-7052 (pager 801-339-0092) or co-investigator, Dr. Roger Faix@ 801-587-7500 (pager 338-2228), in the Department of Pediatrics/Neonatology, University of Utah School of Medicine.

INSTITUTIONAL REVIEW BOARD:

If you have questions regarding your child's rights as a research subject, or if problems arise which you do not feel you can discuss with the Investigator, please contact the Institutional Review Board Office at the LDS Hospital @ 801-408-6781.

INJURY NON-COMPENSATION STATEMENT:

In the event your child sustains an injury resulting from your participation in the research project, Intermountain Medical Center can provide to him/her, emergency and temporary medical treatment and will bill your insurance company. Since this is a research study, payment for any injury resulting from participation in this research study may not be covered by some health insurance plans. If you believe that your child sustained an injury as a result of your participation in this research program, please contact the Institutional Review Board Office at the LDS Hospital @ 801-408-6781.

VOLUNTARY PARTICIPATION:

It is up to you to decide whether or not your baby will take part in this study. If you do decide to have him/her take part you will be asked to sign this consent form. You are free at any time to withdraw from this study without giving a reason. Whether your baby joins this study or not, your baby will receive the same medical and nursing care as needed and it will not affect your relationship with the investigator or other medical staff.

UNFORESEEABLE RISKS:

A particular treatment may involve risks to the baby that are currently unforeseeable. However, because all of the treatments proposed in this study are currently accepted as standard of care, there is no unpredictable increase expected. Unknown risks may be learned during the study, and is so you will be informed by the study personnel. The only other risk of this study is a risk to confidentiality. Every effort will be made to keep your baby's medical information confidential.

RIGHT OF INVESTIGATOR TO WITHDRAW:

You may withdraw your baby from this study at any time without penalty. Dr. Bradley Yoder or his associate investigators can withdraw your baby without your approval. Possible reasons for withdrawal include a need to transfer care to a different hospital not involved in this study or early termination of this study for safety considerations.

COSTS TO SUBJECTS AND COMPENSATION:

There is no cost to parents nor is there any compensation for participating in this study.

NEW INFORMATION:

INTERMOUNTAIN HEALTH CARE
URBAN CENTRAL REGION
IRB

FEB 3 2009	DEC 11 2009
APPROVED	EXPIRATION DATE

Sometimes during the course of a research project, new information becomes available about the treatment that is being studied. If this happens, your research doctor will tell you about it and discuss with you whether you want your baby to continue in the study. If you decide to continue your baby in the study, you will be asked to sign an updated permission form. Also, on receiving new information your research doctor might consider it to be in the best interest of your baby to withdraw him/her from the study. They will explain the reasons and arrange your baby's care to continue.

NUMBER OF SUBJECTS:

We expect to include about 1300 babies in the study from the sixteen NICHD Neonatal Research Network hospitals over a two-year period. Intermountain Medical Center, Primary Children's Medical Center, and the University of Utah Hospital will enroll around 80 babies over the two year period.

CONFIDENTIALITY/ APPROVAL TO USE YOUR CHILD'S PROTECTED HEALTH INFORMATION

IHC has a commitment to protect your child's confidentiality. Federal regulations require that you understand how your child's protected health information (PHI) is used for this study.
Signing this document means you allow us, the researchers in this study, and others working with us to use information about your baby's health for this research study. You can choose whether or not to participate in this research study. However, in order to participate you have to sign this consent and authorization form.

This is the information we will use:
Name
Address
Telephone number
Current and past medications or therapies
Information from a physical examination, such as blood pressure, heart rate, breathing rate, and temperature
Information related to the use of any device for support of lung function such as ventilator pressures, oxygen concentration and blood oxygen levels; any pertinent x-ray studies including head ultrasounds and MRI's; and information related to other neonatal diagnoses.

In records and information disclosed outside of IHC to the NICHD Neonatal Network Data Collection center at Research Triangle Park, North Carolina, your child's information will be assigned a unique code number. We will keep the key to the code in a secure file maintained in the Division of Neonatology, University of Utah School of Medicine.

Others who will have access to your child's protected health information for this research project include IHC's Institutional Review Board (the committee that oversees research studying people) and authorized members of the IHC's workforce who need the information to perform their duties (for example: provide treatment, to ensure integrity of the research, and for accounting or billing matters), the Food and Drug Administration, and others as required by law.

You may revoke this authorization at any time. **This must be done in writing.** You must either give your revocation in person to the Principal Investigator or the Principal Investigator's staff, or mail it to Dr. Bradley A. Yoder, Department of Pediatrics/Neonatology, University of Utah School of Medicine, and PO Box 581289, Salt lake City, UT 84158-1289. If you revoke this authorization, we will not be able to collect new information about your baby, and will be withdrawn from the research study. However, we can continue to use information we have already started to use in our research, as needed to maintain the integrity of the research.
You have a right to information used to make decisions about your baby's health care. However, your baby's information from this study will not be available during the study; it will be available after the study is finished. This authorization lasts until this study is finished.

For more information about rights to your child's protected health information, how to revoke this authorization, and how IHC uses your child's health information, you may ask to see or obtain a copy of the IHC Notice of Privacy Practices.

I hereby acknowledge that I have received or been offered a copy of IHC's Notice of Privacy Practices.

INTERMOUNTAIN HEALTH CARE
URBAN CENTRAL REGION
IRB

FEB 3 2009 DEC 11 2009
APPROVED EXPIRATION DATE

CONSENT:

I confirm that I have read and understand this consent and authorization document and have had the opportunity to ask questions. I understand that my child's participation is voluntary and that I am free to withdraw my child at any time, without giving any reason, without his/her medical care or legal rights being affected. I will be given a signed copy of the consent and authorization form to keep.

INTERMOUNTAIN HEALTH CARE
URBAN CENTRAL REGION
IRB

FEB 3 2009 — APPROVED

DEC 11 2009 — EXPIRATION DATE

I agree to allow my child to participate in this research study and permit you to use and disclose health information about my child for this study, as you have explained in this document.

____
Child's Name

(Please Note: Both parents must give their permission unless one parent is deceased, unknown, incompetent, not reasonably available, or when only one parent has legal responsibility for the care and custody of the child. If both parents are not able to sign, please list the name of the parent and the reason why they are not able to sign in the signature line.

Parent/ Guardian Name	Parent/ Guardian Signature	Title	Date

____
Name of Person Obtaining Authorization and Consent

________________________ ____________
Signature of Person Obtaining Authorization and Consent Date

____
Name of Witness

________________________ ____________
Signature of Witness Date

The Surfactant Positive Airway Pressure and Pulse Oximetry Trial in Extremely Low Birth Weight Infants

Background

You are being asked to allow your baby to be in the study because there is a possibility he/she will be born between 24 and 27 weeks gestation. Before you decide it is important for you to understand why the research is being done and what it will involve. Please take time to read the following information carefully and discuss it with friends, and relatives if you wish. Ask us if there is anything that is not clear or if you would like more information. Please take time to decide whether or not you volunteer your baby to take part in this research study.

Our hospital is conducting this study in cooperation with the National Institutes for Child Health and Human Development (NICHD) Neonatal Research Network to try to determine the optimal means for managing early breathing support in the extremely premature infant (CPAP or a breathing tube) and to determine the appropriate level of oxygen saturation (oxygen levels in the blood) in extremely premature babies. CPAP stands for Continuous Positive Airway Pressure. The pressure is provided by the flow of oxygen and air through prongs or a tube in the nose, and the purpose of the pressure is help keep the lungs inflated making it easier for the baby to breathe and to get adequate oxygen into the blood.

Study Procedures

Prior to delivery**,** and after your permission**,** your baby will be randomized (chosen by chance, like a flip of a coin) to one of two lung treatment strategies. The use of early CPAP and Intubation/Surfactant are both treatments currently used immediately after delivery at this hospital. The decision as which to use is currently made by the physician attending the delivery. The randomized treatments are as follows:

1) CPAP immediately after delivery continuing in the neonatal intensive care unit (NICU), or
2) The placement of a breathing tube (ETT) in the airway immediately after delivery followed by surfactant administration and assisted ventilation (breathing for the baby using a machine)
And,
3) Oxygen saturation levels (O2-SAT's) maintained between 85-89% (low range), or
4) Oxygen saturation levels (O2-SAT's) maintained between 91-95% (high range)
The box below indicates the 4 possible treatment combinations your infant can be randomized to.

CPAP<br>and<br>Higher range oxygen saturation	CPAP<br>and<br>Lower range oxygen saturation
Breathing tube + Surfactant<br>and<br>Higher range oxygen saturation	Breathing tube + Surfactant<br>and<br>Lower range oxygen saturation

FOOTER FOR IRB USE ONLY
Version: F0409

University of Utah
Institutional Review Board
Approved 6/21/2011
Expires 2/22/2012

The physician or nurse caring for your baby will not know which oxygen saturation group the baby is randomized to.

Your baby will remain on the specially modified O2-SAT monitor until he/she reaches 36 weeks corrected age (example: 24 weeks gestation plus 12 weeks of age = 36 weeks adjusted age) or until the monitor is no longer needed because your baby is in room air.

Other care will continue as normal during his/her participation in the study.

A secondary purpose to this study is to determine the longer term effect of different approaches to breathing and oxygen support in the very premature baby. In that regard, this study includes several other planned follow-up evaluations of all study baby's during the first two years of life including:

a. follow up at 6 and 12 months in our high-risk infant follow-up clinic
b. follow-up for subsequent lung problems
c. follow-up at 18-22 months of age for neurodevelopmental function (a complete exam of their muscles, nerves, mental and coordinated movement skills)
d. a comparison of currently applied radiology studies (MRI and Ultrasound) obtained at 36-42 weeks corrected gestation for predicting later neurodevelopmental function

Some of these follow-ups may be by phone and some may be in our special follow-up clinic for very premature infants. The costs of all follow-up evaluations and the MRI examination are covered by the NIH Neonatal Research Network.

Our study coordinator will keep track of how your baby is doing up to 40 weeks gestational age (the babies original due date) or until discharge. Once your baby reaches 36 weeks gestational age we will record if he/she is still being treated with oxygen and/or a ventilator. If your baby is still on nasal cannula oxygen we will try to wean him/her off the oxygen using a standard protocol. If he/she successfully weans off oxygen, your baby's medical team may decide to take him/her completely off oxygen. All information will have a code number and, after discharge, this information will be sent to the NICHD Neonatal Network's Data Collection center at Research Triangle Park, North Carolina.

A further planned follow-up will occur when your baby is about 6 – 7 years old. This will involve neurological, developmental and intelligence testing at a Follow-Up clinic appointment. With your permission we will maintain contact with you in order for your baby to attend the Follow Up appointment when he/she is in grade school.

Risks

All treatments proposed in this study are currently accepted standard of care. All of these treatment options may have risks but there is no known predictable increase in risk to your baby from any one approach. We don't know which approach to treatment is better or safer – that is why we are doing this study. Infants randomized to the CPAP group may, at some point in their care, require intubation and assisted ventilation. If the attending physician deems necessary, participating in this study will not effect this decision. Some unknown risks may be learned during the study. If this occurs, you will be informed by the study personnel. The only other risk in this study is the risk to confidentiality. Every effort will be made to keep your child's medical

University of Utah
Institutional Review Board
Approved 6/21/2011
Expires 2/22/2012

record confidential. There will be no names or other patient identification in any study report that may be published after the study is complete. Measures taken to protect you and your baby's identity are described in the confidentiality.

Benefits
There may be benefits to your child directly, including a possible decrease in chronic lung disease (need for extra oxygen at discharge) and decrease in the need for eye surgery as a result of exposure to oxygen. However, we cannot promise any benefits to your baby from being in this study. The knowledge learned from this study may help us treat babies in the future.

Confidentiality
As an alternative to participation in this study you may decide to have your baby's doctor decide which treatment your baby will receive. If you decide not to include your baby in this study, none of his/her medical information will be included in the study data. Participation in research is entirely voluntary. You may refuse to participate or withdraw at any time without jeopardy to the medical care your baby will receive at this institution. Data collection for this study will stop at that time.

Person to Contact
If you have concerns or questions about this research or any related matter, you can contact the primary investigator, Dr. Bradley Yoder @ 801-581-7052 or co-investigator, Dr. Roger Faix@ 801-587-7500, in the Department of Pediatrics/Neonatology, University of Utah School of Medicine. After hours, please call the NICU directly (University Hospital: 801-581-2775; or Primary Children's Medical Center: 801-588-3800) and ask for the doctor on call. They will be able to answer your questions or contact the above investigators. If you believe your child has been harmed as a result of participation, or if you have any complaints or concerns, please contact the study team.

Institutional Review Board
Contact the Institutional Review Board (IRB) if you have questions regarding your child's rights as a research participant. Also, contact the IRB if you have questions, complaints or concerns which you do not feel you can discuss with the investigator. The University of Utah IRB may be reached by phone at (801) 581-3655 or by e-mail at irb@hsc.utah.edu.

Research Participant Advocate: You may also contact the Research Participant Advocate (RPA) by phone at (801) 581-3803 or by email at participant.advocate@hsc.utah.edu.

Research-Related Injury
If your infant is injured from being in this study, medical care is available at either the University of Utah Hospital or Primary Children's Medical Center, as it is to all sick or injured people. The University of Utah Hospital and Primary Children's Medical Center do not have a program to pay you if your infant is hurt or has other bad results from being in the study. The costs for any treatment or hospital care would be charged to you or your insurance company (if you have insurance), to the study sponsor or other third party (if applicable), to the extent those parties are responsible for paying for medical care you receive. Since this is a research study, some health insurance plans may not pay for the costs.

The University of Utah is a part of the government. If your child is injured in this study, and you want to sue the University or the doctors, nurses, students, or other people who work for the University, special laws may apply. The Utah Governmental Immunity Act is a law that controls when a person needs to bring a claim against the government, and limits the amount of money a person may recover. See Section 63G-7-101 to -904 of the Utah Code. .

Voluntary Participation

It is up to you to decide whether or not your baby will take part in this study. If you do decide to have him/her take part you will be asked to sign this consent form. You are free at any time to withdraw from this study without giving a reason. Whether your baby joins this study or not, your baby will receive the same medical and nursing care as needed and it will not affect the relationship you have with the investigator or other medical staff.

Unforeseeable Risks

The particular treatment or procedure may involve risks to the baby that are currently unforeseeable but because all of the treatments proposed in this study are standard of care, there is no unpredictable increase. If unknown risks are learned during the study, you will be informed by the study personnel. The only other risk of this study is a risk to confidentiality. Every effort will be made to keep your baby's medical record confidential.

Right of Investigator to Withdraw

Your baby may withdraw from the study at any time without penalty. Dr. Bradley Yoder or his associate investigators can withdraw your baby without your approval. Possible reasons for withdrawal include a need to transfer care to a different hospital not involved in this study or early termination of this study for safety considerations.

Costs to Subjects and Compensation

There is no cost to parents nor is there any compensation for participating in this study. Standard costs for care will be billed to you and your insurance company in the usual manner. The exception to this is that the cost of the MRI scan to be obtained at 36 weeks corrected age will be covered by study funds obtained from the NIH Neonatal Research Network and will not be billed to you.

New Information

Sometimes during the course of a research project, new information becomes available about the treatment that is being studied. If this happens, your research doctor will tell you about it and discuss with you whether you want to continue in the study. If you decide to continue your baby in the study, you will be asked to sign an updated permission form. Also, on receiving new information your research doctor might consider it to be in your baby's best interest to withdraw him/her from the study. They will explain the reasons and arrange your baby's care to continue.

Number of Subjects

We expect to include about 1310 babies in the study from the fifteen NICHD Neonatal Research Network hospitals over a two- year period. The University of Utah and LDS Hospital will enroll around 60 babies over the two year period.

Approval to Use Your Child's Protected Health Information

Signing this document means you allow us, the researchers in this study, and others working with us to use information about your baby's health for this research study. You can choose whether or not to will participate in this research study. However, in order to participate you have to sign this consent and authorization form.

This is the information we will use:

- Name
- Address
- Telephone number
- Current and past medications or therapies
- Information from a physical examination, such as blood pressure reading, heart rate, breathing rate, and temperature
- Information related to the use of any device for support of lung function such as ventilator pressures, oxygen concentration and blood oxygen levels; any pertinent x-ray studies including head ultrasounds; information related to other neonatal diagnoses.

Others who will have access to your child's information for this research project are the University's Institutional Review Board (the committee that oversees research studying people) and authorized members of the University's and/or Primary Children's Medical Center workforce who need the information to perform their duties (for example: to provide treatment, to ensure integrity of the research, and for accounting or billing matters). The sponsor of the study does not have the right to inspect patient records.

If we share your child's information with anyone outside the University of Utah Health Sciences Center and/or Primary Children's Medical Center, your child will not be identified by name, social security number, address, telephone number, or any other information that would directly identify him/her, unless required by law. In records and information disclosed outside of the University of Utah Health Sciences Center and/or Primary Children's Medical Center, your child's information will be assigned a unique code number. We will keep the key to the code in a password protected computer. We will destroy the key to the code at the end of the research study.

You may revoke this authorization at any time. **This must be done in writing.** You must either give your revocation in person to the Principal Investigator or the Principal Investigator's staff, or mail it to Dr. Bradley A. Yoder, Department of Pediatrics/Neonatology, University of Utah School of Medicine, and PO Box 581289, Salt lake City, UT 84158-1289. If you revoke this authorization, we will not be able to collect new information about your child and they will be withdrawn from the research study. However, we can continue to use information we have already started to use in our research, as needed to maintain the integrity of the research.
You have a right to information used to make decisions about your baby's health care. However, your baby's information from this study will not be available during the study; it will be available after the study is finished. This authorization lasts until this study is finished.

Consent

I confirm that I have read and understand this consent and authorization document and have had the opportunity to ask questions. I understand that my child's participation is voluntary and that I am free to withdraw my child at any time, without giving any reason, without my medical care or legal rights being affected. I will be given a signed copy of the consent and authorization form to keep.

I agree to allow my child to participate in this research study and permit you to use and disclose health information about my child for this study, as you have explained in this document.

____
Child's Name

____
1st Parent/Guardian's Name

________________________ ____________
1st Parent/Guardian's Signature Date

____
Relationship to Child for 1st Parent/Guardian

____
2nd Parent/Guardian's Name

________________________ ____________
2nd Parent/Guardian's Signature Date

____
Relationship to Child for 2nd Parent/Guardian

Permission cannot be obtained from 2nd parent/guardian because *(please check which one applies to the situation. 45 CFR 46.408):*

____ The parent/guardian is deceased.
____ The parent/guardian is unknown.
____ The parent/guardian is incompetent.
____ The parent/guardian is not reasonably available.
____ Only one parent/guardian has legal responsibility for the care and custody of the child.

____
Name of Person Obtaining Authorization and Consent

________________________ ____________
Name of Person Obtaining Authorization and Consent Date

University of Utah
Institutional Review Board
Approved 6/21/2011
Expires 2/22/2012

WAKE FOREST UNIVERSITY SCHOOL OF MEDICINE AND FORSYTH MEDICAL CENTER

The SUrfactant Positive Airway Pressure and Pulse Oximetry Trial in Extremely Low Birth Weight Infants
(The SUPPORT Trial)

Michael O'Shea, MD, MPH, Principal Investigator

Introduction

You, as a parent or legal guardian of ___________________________, are invited to enter your child in a research study conducted at Forsyth Medical Center and Wake Forest University Baptist Medical Center and sponsored by the National Institutes of Child Health and Human Development (NICHD) Neonatal Research Network. You are being asked to allow your child to be in the study because there is a possibility he/she will be born between 24 and 28 weeks of gestation. Babies born this early frequently develop chronic lung disease (prolonged difficulty with breathing) and retinopathy of prematurity (a condition in which there is overgrowth of the blood vessels in the back of the eye). This study is being carried out to test the benefit of two treatments which may decrease the risk of developing chronic lung disease and retinopathy of prematurity. Approximately 1310 infants, cared for at one of sixteen medical centers in the United States will participate in this study, and about 74 of these babies will be born at Forsyth Medical Center.

Research studies are designed to gain scientific knowledge that may help other babies in the future. Your child may or may not receive any benefit from being part of this study. Participation is voluntary. Please take your time to make your decision, and ask your doctor or the study staff to explain any words or information that you do not understand.

Why Is This Study Being Done?

One of the primary purposes of this study is to find out if the risk of chronic lung disease can be reduced by treatment with continuous positive airway pressure (CPAP) applied immediately after birth while the baby is in the delivery room and continued in the neonatal intensive care unit (NICU). Also, strict guidelines will be used for having a breathing tube placed and mechanical ventilation started. There is no standard way to use CPAP for resuscitation in the delivery room for premature infants. This pressure is given using a mask placed on the baby's face. The pressure may also be given using prongs placed in the infant's nostrils. The pressure is produced using current breathing machines (ventilators). There are also special devices that are designed to deliver such pressures. At the current time very few infants who are born at Forsyth Medical Center and 24 to 27 completed weeks gestation are resuscitated using only CPAP, but this approach has been used for hundreds of babies in other hospitals.

The other purpose of this study is to find out if the risk of retinopathy of prematurity can be reduced by using a lower range for oxygen saturation levels (85-89%) instead of a higher range

WFU School of Medicine
Institutional Review Board
IRB Number: BG04-653
Meeting Date Approved 11/11/2008
Version Valid Until: 12/7/2009

(91-95%). Retinopathy of prematurity (ROP) is a common eye problem in tiny premature infants. Blood vessels that nourish the preterm infant's eyes are not fully developed. Small vessels in the retina (part of the eye) may have periods of increased or rapid growth. Over time ROP can get better or get worse. Usually ROP will heal without any problems. If the ROP is worse than usual, there is a chance that the blood vessels will grow out of control. If this happens, surgery may be needed to prevent scars inside the eye. These scars can cause severe vision loss. The oxygen saturation level currently used in the neonatal intensive care units at Forsyth Medical Center and Brenner Children's Hospital is between 85% and 94%, so both treatment groups (the group for whom the target for oxygen saturation levels will be 85-89% and the group for whom the target for oxygen saturation levels will be 91-95%) will be treated with oxygen in a manner that is very similar to that currently used at both hospitals.

What Is Involved in the Study?

If you give permission for your child to be in this research study, the following will happen:

1) Prior to delivery your baby will be assigned to one of two treatments for babies with respiratory distress. Assignment will be random, like the flipping of a coin. In the first treatment group, your baby will be placed on CPAP in the delivery room immediately after birth and CPAP will continue after transfer of your baby to the NICU. If your baby is in the second treatment group, a tube will be placed in his/her trachea (windpipe) and your baby will be receive help with his/her breathing with a ventilator (a breathing machine). After the tube is placed, a dose of surfactant will be given in the tube. Surfactant is produced by the normal full-term baby lung. It is lacking in very preterm baby lungs and its use has been connected with a decrease in respiratory problems and death.

Both of these treatment groups are current standards of care for preterm babies in the delivery room. The other aspects of the resuscitation will be managed according to the Neonatal Resuscitation Program (NRP) guidelines and follow current hospital practice. Infants randomized to the CPAP group may, at some point in their care, require intubation, assisted ventilation (methods to help them breathe), and surfactant. If the attending physician deems this necessary, participation in the study will not affect this decision. After your infant is admitted to the NICU the study guidelines for intubation, extubation and re-intubation will be in effect for the first 14 days of your baby's life. After 14 days, the ventilation care will be the same as the standard practices in your baby's nursery.

2) Your baby will also be randomized to a high reading or low reading pulse oximeter (a monitor that displays how much oxygen is in the blood). We will start this monitoring of oxygen saturation by 2 hours of age. These oximeters have been modified for use in our research study so that they show a value that is either slightly higher or slightly lower than the true oxygen level when the values are between 85 and 95%. The ranges used in this study are in common use in NICU's across the country. When the true oxygen level is outside those ranges, the actual number will be displayed. This type of oximeter will be used the entire time your baby is on oxygen while he/she is in the NICU until he/she reaches 36 weeks corrected age (age adjusted for prematurity). None of the professionals caring for your baby will know which type of pulse oximeter (high or low reading) your baby is using. This information will be known only by the research coordinator.

WFU School of Medicine
Institutional Review Board
IRB Number: BG04-653
Meeting Date Approved 11/11/2008
Version Valid Until: 12/7/2009

The oximeter group your baby is randomized to will not be known to the nurse, respiratory therapist or physician/s taking care of your baby. Only the study coordinator will know which group your baby is in.

3) If your baby is still receiving oxygen therapy at around 36 weeks corrected age and qualifies for testing, he/she will have an Oxygen Reduction Test. Your baby will stay in his/her bed and a neonatal research nurse will always be present at the bedside. The level of oxygen in the blood will be measured using a standard pulse oximeter. The test will begin with a measurement of the pulse oximeter value at the amount of oxygen your child is receiving. The oxygen level will be lowered in small steps. If the oxygen saturation (or level of oxygen in the blood) remains high, your baby will be placed in room air and monitored for 30 minutes. If your baby's oxygen level goes below the acceptable level (less than 90% for five minutes or 80% or less for 15 seconds), the test will be ended. At the end of the test, your baby will be returned to the oxygen level that he or she was in before the test.

4) As part of the study we will collect information about eye exams. If your baby is transferred to another hospital before discharge home, or requires follow-up eye examinations after discharge we will request a copy of your child's exam.

5) Prior to the time when your infant is discharged from neonatal intensive care, we will contact you to ask about your family, yourself, and your home. We will contact you three more times, either at the time of a visit to our outpatient clinic or by telephone, every 6 months over the next 18-22 months. At these times, we will ask questions about your child's breathing (especially wheezing and coughing), medication use, and visits to a doctor, emergency room, or hospital visits for treatment of breathing problems. We will also ask you several questions about your family and yourself. We expect that answering these questions will take no more than 15 minutes at each contact.

How Long Will My Baby Be in the Study?

The study begins with your agreement to allow your baby to participate in this study. During the initial hospitalization there is no time commitment required from the parent/legal guardian. The treatments that are being tested in this research study will end when your child is discharged. The collection of data will end when your child comes for his/her 18-22 months corrected age visit at the Infant Follow-Up Program at Amos Cottage in Winston-Salem for an evaluation of motor skills, mental development, health and physical growth. These assessments are a part of routine follow-up care for infants born very prematurely. That visit takes approximately 2-3 hours.

In the future we may wish our study participants to return for additional follow-up assessments. Your signature on the line below indicates that you give permission for us to contact you about any further opportunities for follow-up evaluations, after the 18-22 months of age visit.

___________________________________ ___________________________
Signature of parent or legal guardian Date of signature

WFU School of Medicine
Institutional Review Board
IRB Number: BG04-653
Meeting Date Approved 11/11/2008
Version Valid Until: 12/7/2009

What Are the Risks of the Study?

Participation in this study may involve some added risks or discomforts. Because all of the treatments proposed in this study are standard of care, there is no predictable increase in risk for your baby. Some unknown risks may be learned during the study. If these occur, you will be informed by the study personnel.

Are There Benefits to Taking Part in the Study?

There may be benefits to your child directly, including a possible decrease in chronic lung disease and/or a decrease in the need for eye surgery as a result of exposure to oxygen. Because we do not know in advance the actual strategies chosen for your child, or which of the treatment strategies is the most effective, it is also possible that your baby will receive no direct benefit. The knowledge learned from this study may help us treat babies in the future.

What Other Choices Are There?

Your decision to permit your child to participate in this study is voluntary. Your alternative is to not participate in this study.

What about the Use, Disclosure and Confidentiality of Health Information?

Taking part in this research study will involve collecting health information that you consider confidential or private and that directly identifies your baby. Information that identifies your baby includes, but is not limited to, such things as name, address, telephone number, and date of birth. Personal health information includes all information about your baby which is collected or created during the study for research purposes. It also includes your baby's health information that is related to this study and that is maintained in medical records at Forsyth Medical Center, Brenner Children's Hospital and at other such places as additional hospitals or clinics where your baby may have received medical care. Examples of personal health information include health history, the family health history, how your baby responds to study procedures, laboratory and test results, and medical images and information from study-related visits, interactions, and questionnaires. The information gathered from this study will be kept strictly confidential but will be submitted to the National Institute of Child Health and Human Development (NICHD) Neonatal Research Network **without any identification of you or your baby**.

Personal health information and information that identifies you and your baby ("your health information") may occasionally be given to others during and after the study. This is only for reasons such as to carry out the study, to make sure the study is being done correctly, to and to provide required reports.

The health information of your baby **without any identification of you or your baby** may be used by the research team, other researchers and their staff involved with this study or by data management centers; the sponsor of this study, the NICHD, the Federal Office of Human Research Protection, the Food and Drug Administration (FDA), the Wake Forest University School of Medicine Institutional Review Board, The Forsyth Medical Center Institutional Board, or the Office of Clinical Trials, Novant Health Triad Region. The health information of your baby may be disclosed if required by state or federal laws. The results of this study may be published in medical journals, presented at medical meetings, or exchanged between medical investigators**; however your name or your child's name will not be used.** If you have never received a copy of the Wake Forest University Baptist Medical Center Health Insurance Portability and Accountability Act (HIPAA) notice, please ask for one.

When you sign this consent and authorization you give permission for the use of the health information of your baby as described in this consent form. This authorization does not have an expiration date**.** You may decide to withdraw your consent at any time by providing written notification of your decision to Dr. Michael O'Shea at the following address*: Department of Pediatrics, Wake Forest University School of Medicine, Medical Center Boulevard, Winston-Salem, NC 27157*. If you withdraw your authorization your baby will not be able to be in this study. If you withdraw your authorization, no new health information will be gathered after that date, however the information already collected will continue to be used to the extent that it has already been relied on for the study, as necessary to maintain the integrity of the research study or as required by law. Refusal to participate in this study or withdrawal at a later date will not affect the care given to your child by your physician or any other health professionals. This study has been approved by the Wake Forest University Baptist Medical Center Institutional Review Board and Forsyth Medical Center Institutional Review Board, which are committees to help assure that research studies are safe and properly performed.

Although every effort will be made to keep your baby's research-related information private, absolute confidentiality and protection of the information cannot be guaranteed. If information is disclosed to a person or entity that is not covered by the federal privacy regulations it may be re-disclosed. Your baby's research-related information **without any identification of you or your baby** may be used or disclosed until the end of the research study. If the information is included in a research database there is no scheduled date at which it will be destroyed or no longer used. This is because research information continues to be analyzed for many years and it is not possible to determine when this will be complete.

What Are the Costs?

There are no costs to you for taking part in this study. All of the costs related directly to the study, will be paid for by the study. Costs for your regular medical care, which are not related to this study, will be your own responsibility.

Will You Be Paid for Participating?

WFU School of Medicine
Institutional Review Board
IRB Number: BG04-653
Meeting Date Approved 11/11/2008
Version Valid Until: 12/7/2009

You will receive no payment or other compensation for taking part in this study. However, you will receive $100 after completion of the follow-up visit at 18-22 months corrected age to compensate you for time and expenses related to that visit. If your child's birth weight is between 401 (about 14 ounces) and 1000 grams (about two pounds and 3 ounces), this money will be paid as a part of the Follow Up Study. If your child's birth weight is less than 401 grams or more than 1000 grams, this money will be paid as a part of the SUPPORT Trial. In addition, we will assist with travel expenses. To receive payment, you must provide your social security number, name, and address, so that we can comply with Internal Revenue Service (IRS) reporting requirements. When payments are reported to the IRS we do not let them know what the payment is for, only that you have been paid. If you do not wish to provide this information, you can still take part in the study but you will not be paid.

What Happens If My Child Experiences an Injury or Illness As a Result of Participating in This Study?

Should your child experience a physical injury or illness as a direct result of his or her participation in this study, Wake Forest University School of Medicine maintains limited research insurance coverage for the usual and customary medical fees for reasonable and necessary treatment of such injuries or illnesses. To the extent research insurance coverage is available under this policy the reasonable costs of these necessary medical services will be paid, up to a maximum of $25,000. Wake Forest University Baptist Medical Center holds the insurance policy for this coverage. It provides a maximum of $25,000 coverage for each claim, and is limited to a total of $250,000 for all claims in any one year. The Wake Forest University School of Medicine, and The North Carolina Baptist Hospitals, Incorporated do not assume responsibility to pay for these medical services or to provide any other compensation for such injury or illness. Additional information may be obtained from the Medical Center's Director of Risk Management, at (336) 716-3467.

You do not give up any legal rights as a research participant by signing this consent form. For more information on medical treatment for research related injuries or to report a study related illness, adverse event, or injury you should call Dr. Michael O'Shea at (336) 716-2529. After hours you may call (336) 716-7654 or 1-800-277-7654 and ask for Dr. O'Shea to be paged.

What Are My Child's Rights as a Research Study Participant?

Taking part in this study is voluntary. If you choose not to have your baby participate in this study, he/she will receive routine care. You may choose not to take part or you may leave the study at any time. The investigators also have the right to stop participation in the study at any time. You will be given any new information we become aware of that would affect your willingness to continue to have your baby participate in the study.

Whom Do I Call if I Have Questions or Problems?

WFU School of Medicine
Institutional Review Board
IRB Number: BG04-653
Meeting Date Approved 11/11/2008
Version Valid Until: 12/7/2009

Questions about your baby's participation in this research study can be directed to Dr. Michael O'Shea at (336) 716-2529) during business hours and (336) 716-7654 or 1-800 277-7654 before or after business hours. The Institutional Review Board (IRB) is a group of people who review the research to protect your rights. If you have a question about your rights as a research participant, you may contact the Chairman of the Wake Forest University Health Sciences IRB at (336) 716-4542 and/or the Chairman of the Forsyth Medical Center IRB at (336) 718-5960.

Consent

I agree to let my child take part in The SUPPORT Trial. (The Surfactant Positive Airway Pressure and Pulse Oximetry Trial in Extremely Low Birth Weight Infants. I authorize the use and disclosure of my child's health information as described in this consent and authorization form. I have had a chance to ask questions about being in this study and have those questions answered. By signing this consent and authorization form, I am not releasing or agreeing to release the investigator, the sponsor, the institution or its agents from liability for negligence.

(You will receive a copy of this consent form after all signatures have been obtained.)

________________________________	__________________________
Signature of parent or legal guardian	Date of signature
________________________________	
Printed name of parent or legal guardian	
________________________________	__________________________
Signature of person administering consent	Date of signature
________________________________	
Printed name of person administering consent	

WFU School of Medicine
Institutional Review Board
IRB Number: BG04-653
Meeting Date Approved 11/11/2008
Version Valid Until: 12/7/2009

Informed Consent

The Surfactant Positive Airway Pressure and Pulse Oximetry Trial in Extremely Low Birthweight Infants (SUPPORT Trial)

Seetha Shankaran, M.D. Principal Investigator, Wayne State University

INTRODUCTION AND PURPOSE

I am being asked to allow my baby to participate in a clinical research study. Clinical research is the study of human diseases in an attempt to improve diagnosis and treatment. In order to decide whether or not I should agree for my baby to take part in this research study, I should understand enough about its risks and benefits to make a judgment. This process is called informed consent.

This consent form gives information about the research study that will be discussed with me. Once I understand the study, I will be asked to sign this form if I wish for my child to participate. I will have a copy for my child's records.

PROCEDURE:

My baby may be born prematurely. Babies that are born this early are at a higher risk for many more problems than a baby born at full term. I understand that my baby will be at high risk for developing breathing problems requiring oxygen and additional treatment to keep the lungs inflated (open and expanded instead of collapsed).

Seetha Shankaran, MD, her associates, and the National Institutes for Child Health and Human Development (NICHD) Neonatal Research Network are conducting a research study to find out more about treatment with CPAP (positive pressure applied with a face mask to help keep the lungs inflated) and learn the appropriate levels of oxygen saturation (oxygen levels in the blood) in premature babies. Additionally, to learn more about how very premature babies grow in relationship to levels of oxygen saturation. I am being asked to allow my child to be in the study because there is a possibility he/she will be born between 16 and 12 weeks early (24-28 weeks gestational age).

The purposes of this trial are the following:
1) To compare infants who receive delivery room CPAP and who have strict guidelines for having a breathing tube placed (intubated) with infants who have the tube placed and surfactant (a liquid which helps babies with immature lungs breath easier by helping keep their lungs from collapsing) given in the delivery room.
2) To compare low range (85-89%) oxygen saturation levels with high range (91-95%) levels to determine if a lower range results in decreased ROP (Retinopathy of Prematurity), an eye disease that may result in impairment of vision or even blindness, which may be caused by excessive levels of oxygen.
3) To compare babies' growth in relationship to the two levels of oxygen saturation to see if a lower range level results in better growth.

Duration of the Study: To include about 1300 babies in the study from all the NICHD Neonatal Research Network hospitals over a two-three year period.

The Surfactant Positive Airway Pressure and Pulse Oximetry Trial in Extremely Low Birthweight Infants (SUPPORT Trial)

The use of CPAP and Intubation/Surfactant are both treatments currently used in the delivery room at Hutzel Women's Hospital. The decision as to which to use is currently made by the physician attending the delivery.

The oxygen level currently used in the NICU at Hutzel Women's Hospital is between 85% and 95%. Both treatment groups (85-89% and 91-95%) fall within that range. The study will attempt to keep babies in one of these two smaller ranges.

If I agree to allow my child to be in this study, I understand the following will happen to my child: Prior to delivery, and after my permission, my baby will be randomized (chosen by chance like the flip of a coin) to one of two lung treatment strategies. The treatments are as follows:
1) CPAP by mask in the delivery room immediately after birth and continuing in the NICU with prongs in the nose. If my child meets the guidelines for having a breathing tube placed (intubated), then surfactant will be administered at that time or
2) The placement of a tube in his/her trachea (windpipe) in the delivery room followed by surfactant administration and ventilation (breathing for the baby using a machine).

In addition to being randomly assigned to one of the two groups described above, my baby will be randomized to a High reading or Low reading oximeter (a monitor that displays how much oxygen is in the blood). The oximeters (oxygen monitors) used in this trial are FDA approved oximeters which have been modified for research purposes. This modification makes the monitors show a value which is either slightly higher or slightly lower than the true oxygen level when values are between 85 and 95%. Outside those ranges, the oximeter works the same as the standard of care device.

Which group my baby is randomized to will not be known to the nurse taking care of my baby, or his/her physician. Only the study coordinator will know which group my baby is in. Within the range of oxygen which the doctors normally keep babies in, my baby will either be on the high end of normal or the low end of normal. He/she will remain on this device until he/she reaches 36 weeks adjusted age. (e. g. 24 wks gestation plus 12 weeks of age = 36 weeks adjusted age) or until he/she does not need oxygen anymore. Other care will be conducted as normal during his/her participation in the study. My baby will be followed in the Infant Follow-up clinic at 6 and 12 months as standard of care for small babies. At 18-22 months corrected age my baby will receive, at no charge to me, a complete exam of their muscles, nerves, and mental and coordinated movement skills.

To monitor growth, measurements of my baby's weight will be collected from the medical record on the following dates: the day of birth, once weekly for the next four weeks, about eight weeks before the date my baby would have been born at full term, and at discharge to home. On those same dates, measurements of my baby's length using a length board and head circumference (measurement around the head) using a measuring tape will be done.

BENEFITS:

There may be benefits to my child directly, including a possible decrease in chronic lung disease (need for extra oxygen near discharge) and/or a decrease in the need for eye surgery as a result of exposure to oxygen. Because the doctors do not know in advance the actual strategies chosen for my child, or which of the treatment strategies is the most effective, it is also possible that my baby will receive no direct benefit. The knowledge learned from this study may help doctors treat babies in the future.

RISKS:

Participation in this study may involve some added risks or discomforts. Because all of the treatments proposed in this study are standard of care, there is no predictable increase in risk for my baby. Infants randomized to the CPAP group may, at some point in their care, require intubation and assisted ventilation (a breathing machine). If the attending physician deems this necessary, participation in the study will not affect this decision. There may be other risks identified during the study. If there are additional risks, I will be informed about these risks by the study personnel.

ALTERNATIVES:

If my baby does not participate in this study, my infant will receive surfactant therapy and nasal CPAP (CPAP is given by prongs placed in his/her nose) and if needed, be intubated (placed on the breathing machine) with oxygen saturation between 85-95%.

VOLUNTARY PARTICIPATION/WITHDRAWAL:

Participation is voluntary. I can also take by baby out of the study at any time. If I do take my baby out of the study, the hospital and the doctor's will continue to give my baby the best care that they can while my baby is treated in this institution.

COSTS:

There is no cost to me for participation in the study. I will receive no financial compensation for participation in this study except for reimbursement for travel costs. Costs that are part of standard of care are the responsibility of me or my insurance company. I will be provided transportation to the clinic for my follow-up visit.

COMPENSATION:

I understand that in the event of injury resulting from participation in this study, no compensation and no free medical treatment or reimbursement is offered by The Detroit Medical Center, Children's Hospital or Hutzel Women's Hospital, Wayne State University, or any other party involved in this study.

CONFIDENTIALITY:

Information about what the doctors learn from this study may be published or given to other people doing research, but neither my name nor my child's name will be used. The Food and Drug Administration (FDA) may review my child's medical record. The data collected in this study will be submitted to the National Institute of Health that is sponsoring this study. All the

information gathered on my baby as part of this study will be part of his/her medical record and will otherwise be kept confidential to the extent permitted by law.

QUESTIONS:

Any questions I have asked about this study have been answered to my satisfaction. If I have any other questions later on or if I believe my baby has suffered injury as a result of participation in this study, I may call Dr. Seetha Shankaran at 313-745-1436. If I have questions regarding my rights or my child's rights as they relate to my child's participation in this study, I may contact the Chairman of the Human Investigation Committee at 313-577-1628.

If new information develops during the course of the study that will affect my child continuing to be part of the study, the doctors will provide it to me.

By signing this paper, I am saying that I have read it, understand it, and that I agree for my baby to participate in this study.

The Surfactant Positive Airway Pressure and Pulse Oximetry Trial in Extremely Low Birthweight Infants
(SUPPORT Trial)

Printed name of Study Subject ______ Date ______

Signature of Legally Authorized Representative ______ Date ______

Relationship to Subject ______ Date ______

Signature of Witness ______ Date ______

Signature of Investigator/Designee ______ Date ______

APPROVAL PERIOD
MAR 27 '08 MAR 26 '09
HUMAN INVESTIGATION COMMITTEE

NAME:

HOSPITAL UNIT NUMBER:

ADDRESSOGRAPH:

PERMISSION FOR PARTICIPATING IN A RESEARCH PROJECT
YALE UNIVERSITY SCHOOL OF MEDICINE-YALE-NEW HAVEN HOSPITAL

Study Title: The SUrfactant Positive Pressure and Pulse Oximetry Trial in Extremely Low Birth Weight Infants (The SUPPORT Trial)

Principal Investigator: Richard A. Ehrenkranz, MD
Co-Investigator: Vineet Bhandari, MD, DM

Funding Source: The National Institute of Child Health and Human Development (NICHD) Neonatal Research Network

Invitation to Participate and Description of Project

You are invited to participate in a research study designed to determine if using continuous positive airway pressure during resuscitation after birth helps decrease the severity of lung disease in premature babies. We will also be looking at the ranges of oxygen saturation that are currently being used with these same babies. You and your baby have been asked to participate because you are less than 28 weeks pregnant and your baby may be born prematurely. The doctors at the Newborn Special Care Unit (NBSCU) at Yale-New Haven Children's Hospital (Y-NHCH); along with 15 other centers across the country, are participating in this project sponsored by the National Institute of Child Health and Human Development.

In order to decide whether or not you wish to be a part of this research study you should know enough about its risks and benefits to make an informed judgment. This consent form gives you detailed information about the research study, which a member of the research team will discuss with you. This discussion should go over all aspects of this research: its purpose, the procedures that will be performed, any risks of the procedures, possible benefits and possible alternative treatments. Once you understand the study, you will be asked if you wish to participate; if so, you will be asked to sign this form.

Description of Procedures

Your baby may be born prematurely and is at risk for a breathing problem called **Respiratory Distress Syndrome (RDS)**. A baby's lungs are made up of tiny lung sacs; each one is suppose to open and close as the baby breathes in and out. Oxygen is supposed to go in and carbon dioxide is supposed to come out. This works well in full term babies and adults; however, in premature babies the lung sacs don't always work this way. Some lung sacs open and close normally; others collapse and stick together when the baby breathes out making it harder for the baby to breathe. Doctors treat this problem by keeping the lungs slightly inflated (or open) between breaths. This is done by maintaining a little air pressure in the lungs after the baby

breathes out (resting pressure), making it easier for the baby to take the next breath. Sometimes a medication called **surfactant** is given to try to help keep the lung sacs expanded. Surfactant is given directly into the lungs via a tube that is inserted into the airway (referred to as intubation).

After your baby is born, if he/she needs help breathing, the doctor or nurse will place a resuscitation mask over the baby's nose and mouth and provide oxygen and manual breaths with a resuscitation bag. The bag is squeezed to force air into the baby's lungs. The bag and mask may be used to give breaths or give just pressure to keep the lungs inflated (open) between breaths. This resting pressure is called continuous positive airway pressure (CPAP) or positive end-expiratory pressure (PEEP). At the present time, there is no recommendation regarding the early use of CPAP/PEEP in the delivery room and then continuing it in the nursery for premature infants. However, some studies have suggested that the use of early CPAP/PEEP may be associated with improved outcomes such as; fewer babies needing to be placed on a breathing machine, less oxygen use in babies at one month of age and longer, and less need for surfactant to be given. This study will begin in the delivery room and will continue into the nursery. It will compare the use of early CPAP/PEEP with early intubation, surfactant treatment, and initiation of mechanical breathing assistance. The purpose of the study is to see if one of these treatment plans reduces the severity of, and perhaps prevents, the development of long-term lung problems in premature infants. Those lung problems are referred to as bronchopulmonary dysplasia (BPD) or chronic lung disease (CLD).

Another part of the study will be looking at the ranges of oxygen saturations that are currently being used with premature infants. Doctors, nurses, and others taking care of your baby use, in routine daily care, a monitor called a **pulse oximeter** that uses a small probe placed on a hand or a foot to non-invasively monitor oxygen provided to help meet the baby's needs. In this part of the study we would like to pinpoint the exact range that should be used to help prevent some of the problems that occur with premature babies such as Retinopathy of Prematurity (ROP). This is when there is abnormal blood vessel growth in the eye. It causes scar tissue to build up around the retina and if it pulls on the retina hard enough it can cause blindness. It is known that the risk of ROP is increased by the prolonged use of supplemental oxygen from observations published in the 1950's, but the benefit of higher versus lower levels of oxygenation in infants, especially for premature infants, is not known. After reviewing how babies in the past were managed, it has been suggested that the use of lower saturation ranges may result in a lower incidence of severe ROP.

If your baby is born before a gestational age of 28 weeks, he/she will randomly (like the flip of a coin) be placed into the Early CPAP/PEEP group **or** the Early Surfactant/Ventilation group. **Both approaches are currently used at Y-NHCH.**

If your baby is randomized to the Early CPAP/PEEP group, he/she will be treated with CPAP/PEEP in the delivery room and will continue to be supported by CPAP/PEEP after admission to the nursery. However, if at any time, your baby's respiratory distress worsens, he/she will be intubated and started on a breathing machine. If this occurs within the first 48 hours of life, he/she will also be given surfactant.

If your baby is randomized to the Early Surfactant/Ventilation Group, he/she will be intubated and started on the breathing machine in the delivery room and will also be given surfactant within the first hour of birth.

For the first 14 days of life, there will be guidelines for the doctors in the nursery to follow. These guidelines help them decide when to place babies on the breathing machines and when to try and take them off the breathing machines. These guidelines also will help decide when to put them on and take them off of CPAP/PEEP. After 14 days of life, your baby's respiratory care will be managed in accordance with standard NBSCU practice.

The babies in this study will also be placed randomly (again, like the flip of a coin) into a group in which lower oxygen saturation (SpO2) values are targeted or into a group in which higher SpO2 values are targeted. As described above, oxygen saturation is measured on a baby with a monitor called a pulse oximeter. It uses a tiny sensor or probe on the hand or foot of the baby to non-invasively measure how saturated the baby's blood is with oxygen. Oximetry is not painful and provides SpO2 measurements 24 hours a day. The babies in the lower range group will have a target SpO2 of 85-89%, while the babies in the higher range group will have a target SpO2 of 91-95%. All of these saturations are considered within the normal range for premature infants. If the SpO2 falls below 85% or goes higher than 95% then the pulse oximeter will alarm so that the doctors and nurses know when to turn your baby's oxygen up or down.

Therefore, if your baby participates in this study, he/she will be assigned to one of 4 groups.

**Randomized Intervention**	**Low SpO2 85% to 89%**	**High SpO2 91 to 95%**
**Early CPAP/PEEP Group**	Early CPAP + Low SpO2	Early CPAP + High SpO2
**Early Surfactant - Ventilation Group**	Surfactant + Low SpO2	Surfactant + High SpO2

Finally, after discharge home your baby will be invited to return to Yale's Newborn Follow-Up Program located at the Yale Child Study Center. The purpose of this program is to perform standardized developmental testing on all infants cared for in the NBSCU with birth weights less than or equal to 1000 grams. Infants who participate in research projects, such as this one, are also invited to be evaluated in this program. Therefore, at 18-22 months corrected age, your baby will receive, as part of this study, a complete exam of his or her muscles, nerves, and mental and motor skills. In addition, before that visit, brief telephone interviews about your baby's health will be done by our Follow-Up staff.

Risks and Inconveniences

The possible risks of using CPAP/PEEP include stomach fullness and a temporary slowing of the heart rate. Another possible risk is collapsing of one or both lungs. Use of CPAP/PEEP at the level used in this study **does not** increase the risk of a collapsed lung (pneumothorax). As with the use of CPAP/PEEP, a possible risk of being intubated for initiation of mechanical ventilation may include a temporary slowing of the heart rate or possibly the collapse of one or both lungs. Another unlikely risk is the possibility of the airway being punctured during insertion of the breathing tube. **Note** that the risks associated with intubation and initiation of mechanical ventilation **are not increased** by participation in this study.

Pulse oximeters are used routinely in thousands of premature infants in NICU's across the country. There is no known risk to your baby from monitoring with the pulse oximeters. There is a small risk of skin breakdown at the site of the pulse oximeter's probe, but that will be minimized by your baby's nurse routinely changing the probe site.

Benefits

Each of the 4 possible treatment combinations is considered to represent the best approach by some units. However, your baby may not receive any direct benefits from participating in this trial.

If this trial identifies treatment strategies that lower the risk of BPD or ROP, clinical management of extremely preterm infants will be influenced and, hopefully, lead to reductions in the incidence of those problems.

Economic Considerations

All of the therapies and procedures used as part of this study, including early CPAP/PEEP, intubation, mechanical ventilation, surfactant therapy, and pulse oximetry, are commonly used to treat infants in the NBSCU with respiratory problems. Since this study will compare standard therapies, you or your insurer will be responsible for the cost of medical care provided by the staff of the NBSCU to your infant.

A $50.00 gift plus expenses (for example for parking or childcare) will be given to you as compensation for your time after your infant completes the Follow-Up visit at 18 to 22 months corrected age.

Treatment Alternatives/Alternatives

If you choose not to enroll your infant in this study your infant will be treated with respiratory support in accordance with treatment commonly followed in the NBSCU. Currently early CPAP/PEEP, intubation, mechanical ventilation, and surfactant therapy are common practices in the NBSCU for extremely low birth weight infants with breathing problems. As described above, participation in this study will compare standard treatment strategies.

Confidentiality

The medical information gathered from this study and your infant's medical record might be reviewed by representatives of the National Institute of Child and Human Development (NICHD), and the Yale University School of Medicine Human Investigation Committee (the committee that reviews, approves and monitors research on humans). The collection and submission of medical information from this study to the NICHD will be done with professional standards of confidentiality. The results of this study may eventually be published and information may be exchanged between medical investigators. However, your infant's name will not be used in any publication which may result from this research.

In Case of Injury

Although it is highly unlikely that your infant will be injured as a result of his/her participation in this study, treatment will be provided for an injury that is a result of this study. You or your insurance carrier will be expected to pay for the costs of this treatment. No additional financial compensation for injury is available. You do not give up any of your legal rights by signing this form.

Volunteer Participation and Withdrawal

Your infant's participation in this study is purely voluntary. You are free not to give your permission for participation, and refusal to do so will in no way affect the care your baby receives in this unit. Whether or not your infant participates, he/she will be cared for according to standards of newborn care. You are free to withdraw your infant from this study at any time and withdrawal from this study will in no way hurt your relationship with the physicians in the NBSCU or with Yale-New Haven Children's Hospital.

Questions

We have used some technical terms in this form. Please feel free to ask about anything you don't understand and to consider this research and the consent form carefully for as long as you feel is necessary before you make a decision.

Authorization

I have read (or someone has read to me) this form and have decided to participate in the project described above. Its general purposes, the particulars of involvement and possible hazards and inconveniences have been explained to my satisfaction. My signature also indicates that I have received a copy of this consent form.

Name of Subject:______________________________

Signature:____________________________________

Relationship:_________________________________

Date:____

____ ______________________
Signature of Principal Investigator Date

Or

____ ______________________
Signature of Person Obtaining Consent Date

If you have further questions about this project or if you have a research-related problem, you may contact the Principal Investigator [Richard A. Ehrenkranz, MD at (203)-688-2320]. If you have any questions concerning your rights as a research subject, you may contact the Human Investigation Committee at (203) 785-4688.

THIS FORM IS NOT VALID UNLESS THE FOLLOWING BOX
HAS BEEN COMPLETED IN THE HIC OFFICE

THIS FORM IS VALID ONLY UNTIL:
____

HIC PROTOCOL #: 0410027163

INITIALED:
____

CINCINNATI CHILDREN'S HOSPITAL MEDICAL CENTER
CONSENT TO PARTICIPATE IN A RESEARCH STUDY

STUDY TITLE: THE SURFACTANT POSITIVE AIRWAY PRESSURE AND PULSE OXIMETRY TRIAL IN EXTREMELY LOW BIRTH WEIGHT INFANTS (THE SUPPORT TRIAL)

SPONSOR NAME:
National Institutes of Health (NIH) /National Institute of Child Health and Human Development (NICHD)

INVESTIGATOR INFORMATION:

Vivek Narendran, MD	(513)-803-0961	(513) 820-3879
**Principal Investigator Name**	**Telephone Number**	**24 hr Emergency Contact**

Subject Name: ______________________________**Date of Birth**: _____/_____/____

Throughout this document, references to "You" may stand for either the research study subject or for the parents or legal guardians of the research study subject if the subject is under 18 years of age or otherwise unable to legally give informed consent to participate in the research study. The signature(s) at the end will clarify whether the research study subject is signing this consent form on their own behalf or via a legal guardian or legal personal representative.

INTRODUCTION:

You have been asked to participate in a research study. Before agreeing to participate in this study, it is important that you read and understand the following explanation. It describes, in words that can be understood by a lay person, the purpose, procedures, benefits, risks and discomforts of the study and the precautions that will be taken. It also describes the alternatives available and the right to withdraw from the study at any time. No guarantee or assurance can be made as to the results of the study. Also, participation in the research study is completely voluntary. Refusal to participate will

involve no penalty or loss of benefits to which you are otherwise entitled. You may withdraw from the study at any time without penalty.

WHY IS THIS RESEARCH BEING DONE?

The purpose of this research study is to determine the best way to care for very premature infants to reduce the risk for lung disease and eye disease, and to see whether the treatment your infant receives, as part of this study, will improve breathing during the 6-22 months after his/her expected due date.

Very premature infants less than 28 weeks gestational age often develop lung disease and eye disease which may lead to long term disability or death. This lung disease and eye disease may be caused by the kind of treatment that is used normally in the delivery room and in the nursery. This current treatment can cause either collapse of the lungs or too much expansion of the lungs. This may cause injury to the lung leading to long term lung disease or possibly death.

Too much oxygen in the beginning days of life can cause the blood vessels in the eye to grow abnormally. At the same time, not enough oxygen can adversely affect an infant's growth and brain development. Your infant's oxygen is monitored by a machine called a pulse oximeter. This machine tells ~~us~~ how much oxygen is in the blood stream. The study doctors are trying to find the best oxygen level to prevent these lung and eye diseases.

WHY HAVE YOU BEEN ASKED TO TAKE PART IN THIS RESEARCH STUDY?

You are being asked to take part in this research study because you may deliver your infant early. Infants that are delivered early, or premature, are at a higher risk of developing lung disease and eye disease.

WHO SHOULD NOT BE IN THE RESEARCH STUDY?

- Anyone who delivers outside the hospital center
- Anyone who delivers before 24 weeks gestation or after 28 weeks gestation
- If your infant has a known birth defect

HOW LONG WILL YOUR INFANT BE IN THE RESEARCH STUDY?

Your infant's participation in this research study will last until the age of 22 months. The doctor who is caring for your infant may decide to take your infant off this research study at any time if he/she feels it is necessary.

During that time, we will gather information from your infant's medical record, including results of routine NICU assessments, such as head ultrasounds and ophthalmologic exams.

All infants with a birth weight of $\leq$ 1500 grams have an eye exam at 32 weeks post menstrual age or 5-6 weeks of age, whichever comes last. For purposes of the SUPPORT Trial, the results of these exams will be recorded until the infant's eyes reach final status. This may include exams after discharge home or transfer to another hospital.

The majority of this study will take place while your infant is in the hospital. Once he/she has been discharged, there will be one more assessment done at a routine 18 - 22 month follow-up visit at the High Risk clinic.

At the follow-up visit, your child will have a basic health check, as well as a neurological examination. After being weighed and measured, your child will meet with the developmental psychometrist, and the Bayley Scales of Infant Development will be administered. All tests are standard tests frequently used in the assessment of child development, child behavior, and neurological function.

The following questionnaires will be completed by the psychometrist through an interview with the caregiver:

1) Socio-Economic Status (SES) at nursery discharge.
2) Socio-Economic Status (SES) at 18 + 4 months of age.
3) Medical History
4) Family Resource Scale - measures the adequacy of different resources in the household.
5) The Brief Infant-Toddler Social and Emotional Assessment (BITSEA).
6) SUPPORT Trial Breathing Outcomes Study: 6 - 12 Month Interview (SUPF02 Rel 1.0)
7) SUPPORT Trial Breathing Outcomes Study: 8 - 22 Month Interview (SUPF03 Rel 1.0)

A more complete description of all these tests is available as an appendix to this consent form if requested. All these tests will be administered by a single developmental psychometrist. All children will be examined in the study program.

WHO IS CONDUCTING THE RESEARCH STUDY?

This study is sponsored by National Institutes of Health (NIH)/National Institutes of Child Health & Human Development (NICHD).

The study is directed by Vivek Narendran, MD, the researcher at Cincinnati Children's Hospital Medical Center.

HOW MANY PEOPLE WILL TAKE PART IN THE RESEARCH STUDY?

Approximately 1345 infants will be asked to take part in this study.

The NICHD Neonatal Research Network has 16 sites taking part in this study. The University Hospital, the Good Samaritan Hospital, and the Cincinnati Children's Hospital comprise the Cincinnati site within the Neonatal Research Network.

WHAT IS INVOLVED IN THE RESEARCH STUDY?

Neither you, nor the researcher conducting this study will know what group your infant will be assigned. Your infant will have a one in four chance of being placed in any group. However, in the event of an emergency, your study doctor will be able to find out which treatment you are receiving.

In the hospital:

If you agree to have your infant participate in this study, he/she will be assigned to one of the four study groups described below. A flip-of-the-coin method will be used to assign infants to a group.

The four study groups are as follow:

- Strong efforts will be made in this group to keep infants off the ventilator (breathing machine) and the infants will be assigned to a lower blood oxygen range of 85% to 89%.
- Strong efforts will be made in this group to keep infants off the ventilator (breathing machine) and the infants will be assigned to a slightly higher blood oxygen range of 91% to 95%.
- Routine standard care will be practiced in this group and infants will be assigned to a lower blood oxygen level of 85%-89%.
- Routine standard care will be practiced in this group and infants will be assigned to a higher blood oxygen level of 91%-95%.

Strong efforts to keep infants off the ventilator include the use of continuous positive airway pressure or CPAP. This provides some pressure to keep the lungs expanded, but allows the infant to breathe on his/her own. Routine standard care will include the use of a ventilator (breathing machine) and a medicine called surfactant. This medicine is put directly in the lungs through the breathing tube in your infant's throat and helps your infant to breathe easier.

Because oxygen affects how well infants grow, measurements of your infant's weight, length and head circumference will be taken from birth until hospital discharge. Nutrition information will also be collected.

Optional:

It is not known whether breathing extra oxygen as treatment for lung problems causes a depletion of anti-oxidants or a build up of oxidants. These substances may alter lung growth and development and make premature babies more likely to wheeze in early childhood. Anti-oxidant levels can be detected in the blood. One milliliter of blood from the placenta will be collected and analyzed for anti-oxidant levels. Two thirds of one milliliter of blood (0.67cc or 13 drops) will also be collected from your infant when he or she is 14 days and 28 days old. The total amount of blood is 1.3 milliliters (1.3 milliliters is equal to 24 drops or approximately one - fifth of a teaspoon) over a 1 month time period. This amount of blood is considered safe for purposes of research by the Federal Government's National Institute of Health. Every attempt will be made to draw these blood samples at the same time as blood testing that is done as part of routine care in the NICU. You may accept or decline this extra blood collection.

☐ Accept ☐ Decline

After discharge:

Once your infant is discharged, we will continue to stay in touch with you and your infant, by telephone, or in person, at one of your routine visits at the High Risk Infant Follow-Up Clinic at Children's Hospital, every six months over the next 6-22 months.

During this routine medical check-up, trained staff will meet with you and your child to ask you some questions, check your child's growth measurements, and check your child's level of development by interacting with him/ her.

There will be a total of 3 study visits or calls during this time. During these clinic visits and/ or telephone calls, we will ask questions about your child's breathing (especially wheezing and coughing), medication use, and visits to a doctor, emergency room, or hospital for treatment of breathing problems. We will also ask you several questions about your family and yourself. Answering the study questions should take about 15 minutes of your time, less if your baby has had no breathing problems.

The telephone calls will be scheduled when your infant is 6, 12, and 18 months after his/her expected delivery at full term. We will schedule the calls at a time that is convenient to you.

The results from your infant's questionnaire will be combined with the results of other infants from around the country; however, your infant's name will not be used.

WHAT ARE THE RISKS AND DISCOMFORTS OF THE RESEARCH STUDY?

Trying to keep infants off the breathing machine, and on CPAP, may lead to: a) an increased respiratory effort, b) brief periods of a pause in their breathing (apnea), ~~and~~ c) higher carbon dioxide in their blood, and d) nasal septum breakdown. Safeguards are in place to monitor these side effects. This may include the use of a ventilator (breathing machine) when appropriate.

Trying to leave infants on the ventilator (breathing machine) may lead to: a) worsening lung injury due to the pressures used by the machine, b) mechanical complications such as obstruction or loss of airway, and c) prolonged hospital stay. Safeguards are in place to monitor these side effects. The doctor will make changes as needed.

For the optional portion of this study, every attempt will be made to draw the blood samples at the same time as blood testing for routine care. In the event that it is necessary to draw blood by venipuncture -- clinical personnel will take the small amount of blood from a vein in your baby's arm or leg by needle stick, or by heelstick. Risks associated with drawing blood from your baby's arm or leg include momentary discomfort and/or bruising. Infection, excess bleeding, clotting, and/or fainting are also possible, although unlikely.

There may be unknown or unforeseen risks associated with study participation.

One of the risks of participating in research is the loss of confidentiality. Please see confidentiality section.

ARE THERE DIRECT BENEFITS TO TAKING PART IN THE RESEARCH STUDY?

There may be a direct benefit to your child participating in this study, in that information about his/ her development may be learned during the 6 -22 month follow-up study period, following discharge from the hospital. This information will be gathered from the assessments and your child's interaction with study personnel during this period.

WHAT OTHER CHOICES ARE THERE?

You may decide to **not** allow your infant to take part in this research. You may choose to have your infant treated with the usual standard medical care as determined by the doctor. If you refuse participation in this study, it will not change your infant's care in the nursery.

If you decide to allow your infant to take part in this research, you will receive any new information during the course of the study concerning significant treatment findings. This may affect your willingness to continue your infant's participation and you may withdraw your infant from the study at any time.

HOW WILL INFORMATION ABOUT YOU BE KEPT PRIVATE AND CONFIDENTIAL?

Every effort will be made to maintain the confidentiality of your medical information and your infant's medical or research information. Medical record information and potentially identifying information such as your infant's name and birth date are classified as "Protected Health Information" or "PHI".

Protected Health Information is defined as health information, whether verbal or recorded in any form (such as on a piece of paper or entered into a computer), that identifies you, or your infant, as an individual, or offers a reasonable basis to believe that the information could be used to identify you or your infant.

Study records that identify your child will be kept confidential as required by law. Federal Privacy Regulations provide safeguards for privacy, security and authorized access. Except when required by law, your child will not be identified by name, social security number, address, telephone number, or any other direct personal identifier in study records disclosed outside of Cincinnati Children's Hospital Medical Center.

By signing this consent form you are giving permission for representatives of the Cincinnati Children's Hospital Medical Center ("CCHMC"), the investigator and CCHMC employees involved with the research study, including the Institutional Review Board and the Office for Research Compliance, and/ or their appointed agent, as well as the National Institutes of Health, to inspect sections of your medical records and your infant's medical and research records related to this study.

When a study is submitted to the FDA, the clinical investigator agrees to allow the FDA access to the study records. The FDA will treat the information as confidential, but on rare occasions disclosure to third parties may be required by law. Therefore, absolute protection of confidentiality cannot be promised or implied.

A Data and Safety Monitoring Board, an independent group of experts, will be reviewing the data from this research throughout the study. The investigator will tell you about new information from this or other studies that may affect your health, welfare, or willingness to stay in this study.

The information from the research study may be published; however, you will not be identified in such a publication. The publication will not contain information about you that would enable someone to determine your identity as a research participant without

your authorization.

Cincinnati Children's Hospital Medical Center and/or the Investigator will take the following precautionary measures to protect your privacy and confidentiality of your research and/or medical records: and those of your infant.

No information such as name, address, telephone number, etc. that could be used to easily identify your infant will be kept as part of the research records.

A copy of this consent form will be included in your infant's medical research record.

Your infant will be registered in the Cincinnati Children's Hospital Medical Center's computer system as a research subject which may be beneficial for future clinical care**.**

USE AND DISCLOSURE OF YOUR PROTECTED HEALTH INFORMATION

The Protected Health Information described in the section above will be used /disclosed for the purpose of research by CCHMC to the other persons or entities identified above.

"Use" of an individual's health information is defined as the sharing, examination or analysis (break down) of the information that is collected and maintained for the length of the research study.

"Disclosure" of an individual's health information is defined as the release, transfer, providing access to, or to reveal in any other manner, the information outside the persons or entity holding the information as described in the section "How Will Information About You Be Kept Private And Confidential" in this consent form.

Once your Protected Health Information is disclosed, the information may be subject to re-disclosure and may no longer be protected by the federal privacy regulations.

AVAILABILITY OF INFORMATION?

As indicated above, you will receive any new information during the course of the study concerning significant treatment findings.

WHAT ARE YOUR COSTS TO BE IN THIS STUDY?

The procedures of this study are standard methods of caring for premature infants and will be billed to your infant's hospital account. There will be no additional studies or assessments, including head ultrasounds, performed for study purposes only; all studies are clinically indicated.

Funds are not available to cover the costs of any ongoing medical care and you remain responsible for the cost of non-research care.

If you have questions about your medical bill relative to research participation, you may contact Dr. Vivek Narendran by calling (513) 558-0557.

WILL YOU BE PAID TO PARTICIPATE IN THIS RESEARCH STUDY?

There is no payment or reimbursement for participation in this research study.

WHAT COMPENSATION IS AVAILABLE IN CASE OF INJURY?

If you believe that you have been injured as a result of participation in biomedical or behavioral research you are to contact Dr. Vivek Narendran by calling (513) 558-0557 or the Director of Social Services (513-636-4711) to discuss your concerns. Cincinnati Children's Hospital Medical Center follows a policy of making all decisions concerning compensation and/or medical treatment for physical injuries occurring during or caused by participation in biomedical or behavioral research on an individual basis.

WHAT ARE YOUR RIGHTS AS A PARTICIPANT?

Your participation in this study is completely **voluntary**. You may choose either to take part or not to take part in this research study. Your decision whether or not to participate will not result in any penalty or loss of benefits to you and the standard medical care for your condition will remain available to you.

If you decide to take part in the research study, you are **free to withdraw** your consent and discontinue participation in this research study at any time. Leaving the study will not result in any penalty or loss of benefits to you.

You may revoke (choose to withdraw) this Authorization as provided under the Health Insurance Portability and Accountability Act of 1996 (HIPAA") at any time after you have signed it by providing Dr. Vivek Narendran with a written statement that you wish to withdraw this Authorization. Your withdrawal of this Authorization will be effective immediately and your Protected Health Information can no longer be used/disclosed for research purposes by CCHMC and the other persons or entities that are identified in the "Use or Disclosure of Your Protected Health Information" section of this consent, except to the extent that CCHMC and/or the other persons or entities identified above have already taken action in reliance upon your consent. In addition, your Protected Health Information may continue to be used / disclosed to preserve the integrity of this research study.

The investigators will tell you about significant new findings developed during the course of the research and new information that may affect your health, welfare, or willingness to stay in this study.

If you have questions about the study, you will have a chance to talk to one of the study staff or your regular doctor. Do not sign this form unless you have had the chance to ask questions and have received satisfactory answers.

Nothing in this consent form waives any legal rights you may have nor does it release the investigator, the sponsor, the institution, or its agents from liability for negligence.

For further information about your rights, please see CCHMC Notice of Privacy Practices. A copy of the CCHMC Notice of Privacy Practices may be obtained from any patient registration area or online at www.cincinnatichildrens.org (From the internet page select in the following order: About Us, Corporate Information, HIPAA). You may also contact our Privacy Officer at 513-636-4707 to obtain a copy.

ABILITY TO CONDITION TREATMENT ON PARTICIPATION IN THIS STUDY

You have a right to refuse to sign this consent to use/disclose your Protected Health Information for research purposes.

If you refuse to sign this consent, you may not be able to receive research-related treatment.
If you refuse to sign this consent, your rights concerning treatment, payment for services, and enrollment in a health plan or eligibility for benefits will not be affected.

WHOM DO YOU CALL IF YOU HAVE QUESTIONS OR PROBLEMS?

For questions about this research study or to report a research-related injury, you can contact the researcher Dr. Vivek Narendran at (513) 558-0557***.*** Researchers are available to answer any questions you may have about the research at any time.

If you have general questions about your rights as a research participant in this research study, you may call the Cincinnati Children's Hospital Medical Center Institutional Review Board at (513) 636-8039.

CINCINNATI CHILDREN'S HOSPITAL MEDICAL CENTER
CONSENT TO PARTICIPATE IN A RESEARCH STUDY

STUDY TITLE: THE SURFACTANT POSITIVE AIRWAY PRESSURE AND PULSE OXIMETRY TRIAL IN EXTREMELY LOW BIRTH WEIGHT INFANTS (THE SUPPORT TRIAL)

INVESTIGATOR INFORMATION:

Principal Investigator Name	Telephone Number	24 hr Emergency Contact
Vivek Narendran, MD	(513) 558-0557	(513) 820-3879

SIGNATURES:

I have read the information given above. The investigator or his/her designee have personally discussed with me the research study and have answered my questions. I am aware that, like in any research, the investigators cannot always predict what may happen or possibly go wrong. I have been given sufficient time to consider if I (or my child) should participate in this study. I hereby consent for myself (or my child) to take part in this study as a research study subject.

_______________________________ Date:____________
Parent/Legal Guardian (Signature)

_______________________________ Date:____________
Parent/Legal Guardian (Signature)

_______________________________ Date:____________
Investigator or specific individual who
has been designated to obtain consent (Signature)

_______________________________ Date:____________
Investigator (Signature)

This research study and consent form have been reviewed and approved by the Cincinnati Children's Hospital Medical Center Institutional Review Board (telephone number 513-636-8039).
